# A general strategy towards activatable nanophotosensitizer for phototoxicity-free photodynamic therapy

**DOI:** 10.7150/thno.100597

**Published:** 2025-01-01

**Authors:** Guozhu Tan, Qinjie Zhong, Jibin Zhang, Peiyi He, Xiaoxi Zhao, Guifeng Miao, Yafei Xu, Xiaorui Wang

**Affiliations:** 1 Department of Orthopaedics and Traumatology, The Seventh Affiliated Hospital, Southern Medical University 528000, Foshan, Guangdong Province, China.; 2 Biomaterials Research Center, School of Biomedical Engineering, Southern Medical University 510515, Guangzhou, Guangdong Province, China.; 3 Department of Cardiovascular Surgery, Zhujiang Hospital, Southern Medical University, 510280, Guangzhou, Guangdong Province, China.

**Keywords:** nanophotosensitizer, cation-π interactions, phototoxicity, tumor-targeted therapy

## Abstract

**Background:** Photodynamic therapy (PDT) has gained widespread attention in cancer treatment, but it still faces clinical problems such as skin phototoxicity. Activatable photosensitizers offer a promising approach to addressing this issue. However, several significant hurdles need to be overcome, including developing effective activation strategies and achieving the optimal balance between photodynamic effects and related side effects. Herein, we present a novel and general strategy for the construction of tumor-targeted activatable nanophotosensitizers (TNP1/PSs).

**Methods:** TNP1/PSs were constructed through simple nanoprecipitation method, leveraging the strong cation-π interaction between cationic polymers and aromatic photosensitizers. We conducted a comprehensive characterization and investigation of the photoactivity, as well as the mechanisms underlying both OFF state and switched-on properties of TNP1/PSs. Additionally, we thoroughly evaluated the cytotoxicity, tumor-targeted ability, and anti-tumor efficacy of TNP1/PSs in the 4T1 cell line.

**Results:** TNP1/PSs exhibit a markedly fully OFF state of photoactivity, subsequent to self-assembly through cation-π interactions in aqueous media. The mechanism study reveals a multi-pathway process induced by cation-π complexes, which includes reduced absorption and radiative decay, as well as enhanced thermal decay and intermolecular charge transfer. Upon targeting tumor cells, TNP1/PSs were effectively endocytosed and predominantly traversed the lysosomes, where degradation of the cationic polymer occurs, resulting in the spontaneous switch-on of PDT activity. *In vivo* studies employing small animal models demonstrated that the as-synthesized nanophotosensitizer possesses remarkable anti-tumor activity while completely avoiding skin phototoxicity.

**Conclusion:** This work provides a powerful platform for efficiently constructing tumor-targeted activatable nanophotosensitizers, paving the way for safe and effective photodynamic therapy in cancer treatment.

## Introduction

Photodynamic therapy (PDT) has garnered significant attention in the treatment of cancers and other diseases due to its high spatial and temporal selectivity, as well as non-invasive properties 1-3. To date, an increasing number of photosensitizers (PSs) have been commercialized or utilized in clinical trials, such as Photofrins (porfimer sodium), Visudyne (Verteporfin or BPDMA), and 5-aminolevulinic acid (ALA) 2,4. Although significant progress has been made in enhancing the efficacy of PDT, the non-specific distribution of photosensitizers during treatment frequently results in unavoidable side effects 4,5.

The limited tumor specificity and the so-called "Always-On" photoactivity of traditional photosensitizers often give rise to unintended stimulation, superfluous activation, and significant skin phototoxicity 6-8. Moreover, the sluggish metabolic rate of photosensitizers necessitates that patients avoid sunlight exposure for several weeks post-treatment. This limitation not only impedes the feasibility of photodynamic therapy but also adversely affects patients' quality of life 9-11. Evidently, the limited tumor specificity and the "Always-On" characteristic of current photosensitizers significantly hinder their clinical application 12-14.

Enhancing delivery to the target sites and ensuring the specific activation of photosensitizers within tumor tissues are two promising approaches for addressing the aforementioned issues 15. Developing nanophotosensitizers with tumor-targeting capabilities can greatly enhance their accumulation in tumor sites 16-20. Despite the rapid advancements in nanomedicine, the existing drug delivery systems remain impeded by a low delivery efficiency (approximately 0.7%), which falls short in mitigating skin phototoxicity 21-23. Hence, the development of photosensitizers specifically designed for exclusive activation at tumor sites is highly promising for facilitating targeted and on-demand photodynamic therapy 24-26. In this regard, photosensitizers that are activated by stimuli from the tumor microenvironment, termed activatable photosensitizers (APSs), have been developed 27-29. Typically, the design of APSs is based on the principle of suppressing the fluorescence emission of PSs and blocking the energy transfer pathways from PSs to oxygen following light irradiation 30,31. Integrating photosensitizers with energy/electron transfer processes, such as FRET (Förster Resonance Energy Transfer) and PET (Photoinduced Electron Transfer) has been widely recognized as an effective strategy 16,21,29,32. These APS systems typically employ a strategy of covalently linking quenchers to PSs *via* cleavable linkers 33. This linker can be cleaved under pathophysiological stimuli such as enzymes 34,35, redox 36-38 and pH 39, leading to the release of PSs and the recovery of photoactivity. Theoretically, suppressing photoactivity in blood circulation and non-tumor tissues helps to mitigate skin phototoxicity, while its activation within tumor lesions reinstates the anti-tumor effect 40,41.

Recently developed activatable photosensitizer systems, based on the strategies mentioned above, have demonstrated a reduction in skin phototoxicity during cancer treatment. However, there are still some challenges, such as intricate molecular structure design, time-consuming and costly synthesis processes, and potential toxicity issues arising from the quencher moieties on APSs 30,42,43. Moreover, incomplete inhibition of photoactivity posed non-negligible side effects on normal tissues. Most importantly, only a limited number of photosensitizers with specific structural characteristics can be developed into activatable photosensitizers, which restricts the versatility of constructing APSs.

On the other hand, leveraging the versatile nature of supramolecular interactions, platforms based on supramolecular assembly are currently emerging as an alternative strategy for the construction of activatable nanophotosensitizers 7. In general, activatable nanophotosensitizers can be synthesized by conjugating PSs onto a polymeric backbone or encapsulating them within amphiphilic block copolymers. This process may result in the self-quenching of PSs, coinciding with the formation of supramolecular nanostructures 44-46. Indeed, the suppression of photoactivity in nanosystems via conventional supramolecular interactions (including hydrophobic interaction, π-π stacking, and electrostatic forces) has been proven to be limited to some extent, such as the switch-on photoactivity in blood circulation, which results in inadequate mitigation of skin phototoxicity 43,47,48. Consequently, there is a critical demand for the development of simpler, more efficient, and universal strategies for construction of activatable photosensitizers. By exploring novel materials and mechanisms, stronger and more reliable control over photoactivity can be achieved.

**Figure 1:**
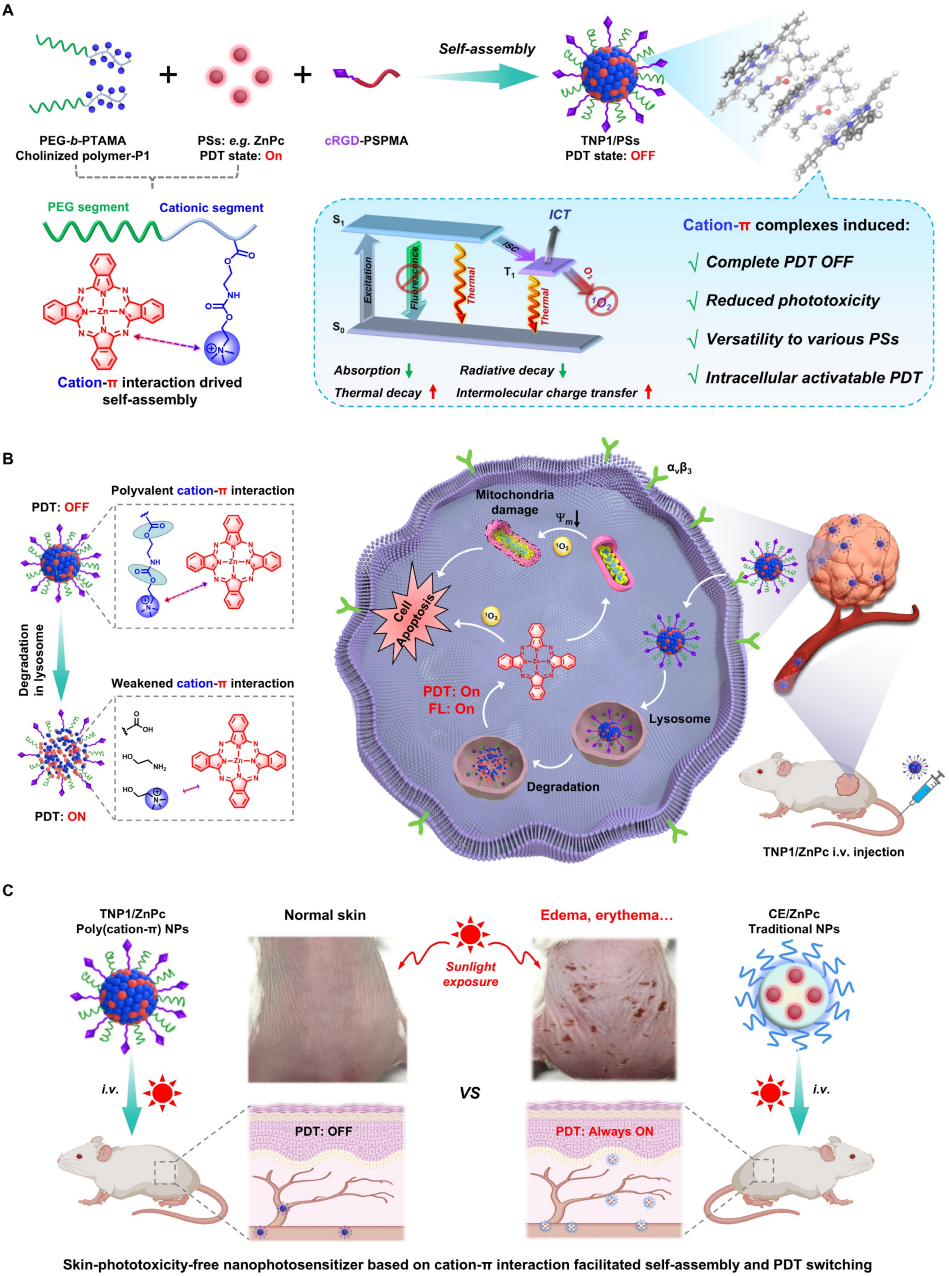
Schematic illustration of the general strategy towards the construction of activity-switchable nanophotosensitizer for skin-phototoxicity-free and tumor-targeted photodynamic therapy (PDT)**.** (A) The self-assembly of tumor-targeting photosensitizer (PS) called TNP1/PS was driven by cation-π interactions. This interaction occurs between cationic species within cholinized polymer (P1) and aromatic rings within PS. Unexpected, the formation of cation-π complexes was found to induce a complete OFF of the PDT activity of TNP1/PS. This deactivation was discovered to occur through a multi-pathway mechanism, including reduced absorption and radiative decay, while enhanced thermal decay and intermolecular charge transfer. (B) Schematic illustration of the spontaneous recovery of PDT activity of TNP1/PS (e.g. TNP1/ZnPc) in tumor cells *via* lysosome-mediated degradation, enabling tumor-targeted photodynamic therapy following intravenous administration (i.v.). (C) The skin-phototoxicity-free of nanophotosensitizer in the form of poly(cation-π) NPs (e.g. TNP1/ZnPc), compared to the significant skin-phototoxicity of traditional NPs (e.g. CE/ZnPc formulated by Cremphor EL).

Cation-π interactions, as a distinct category of supramolecular interactions, are highly regarded for their crucial role in biological systems and materials science 49-53. Recently, our group developed an innovative nanosystem, named poly(cation-π) micelles, which harnesses cation-π interactions to facilitate supramolecular assembly 54. In light of the pervasive presence of π structures in chemical drugs, we have developed a universal drug delivery platform based on cation-π interactions that demonstrates effective performance in tumor therapy. Herein, building upon our previous research, we aimed to construct novel poly(cation-π) nanophotosensitizers by utilizing the strong cation-π interaction between cationic polymers and aromatic photosensitizers (Figure 1A). In brief, we designed and synthesized hydrophilic diblock copolymers featuring choline moieties as cationic segment, namely cholinized polymer (P1). In contrast to the conventional self-assembly process of amphiphilic block polymers that depends on hydrophobic interactions and π-π stacking, the TNP1/PSs structure is formed through the self-assembly of P1 and PSs, driven by robust cation-π interactions.

It is surprising and interesting that when self-assembling in aqueous medium, TNP1/PSs exhibits a complete OFF state of both photoactivity and fluorescence (FL). Mechanistic studies revealed that the multi-pathway process induced by cation-π complexes, including reduced absorption and radiative decay, alongside enhanced thermal decay and intermolecular charge transfer (ICT), collectively contribute to the complete PDT OFF state of TNP1/PSs. By leveraging the unique mechanism that completely inhibits photoactivity, and taking into account the extensive π-structural traits of photosensitizers, we investigated the universality of the PDT OFF function across various photosensitizers within the excitation wavelength range of 400 nm to 800 nm, employing TAPP, ZnPc, and IR780 as representative examples.

Upon targeting tumor cells, TNP1/ZnPc (employing a near-infrared excitable photosensitizer (Zinc phthalocyanine, ZnPc) as proof of concept and incorporating the cRGD peptide *via* a multi-ion complex interaction between P1 and sulfated polymers (cRGD-PSPMA)), were effectively endocytosed and predominantly transited through lysosomes (Figure 1B). Within tumor lysosomes, the hydrolysis of the ester bonds and carbamates in TNP1/ZnPc results in the degradation of the cationic polymers. This process facilitates the transition from strong multivalent cation-π interactions to weaker cation-π interactions. Concurrently, the photoactivity of ZnPc can be spontaneously activated. Mitochondria are considered as the important “powerhouses” of the cell and exhibit a higher susceptibility than other subcellular organelles. Thus, photosensitizers with mitochondria-targeted properties will cause much higher cytotoxicity and achieve excellent PDT outcome 55. Interestingly, in our study, activated ZnPc was observed to further localize within the cytoplasm and mitochondria, demonstrating substantial anti-tumor efficacy, which was fully confirmed by both *in vitro* and *in vivo* studies after mild near-infrared irradiation. Crucially, benefitting from the full OFF of the nanophotosensitizer in the bloodstream prior to its entry into tumor cells, the intravenous administration of TNP1/ZnPc does not cause noticeable skin phototoxicity or any other adverse side effects even when exposed to simulated sunlight (Figure 1C). This contrasts markedly with the significant skin-phototoxicity of the traditional nanophotosensitizers formulated with amphiphilic molecules (e.g. CE/ZnPc using Cremphor EL). Overall, we have successfully developed a novel nanophotosensitizer with non-skin-phototoxic, leveraging the cation-π interaction-driven self-assembly and switchable photoactivity.

## Results and Discussion

### Preparation and characterization of poly(cation-π) nanophotosensitizers

**Figure 2:**
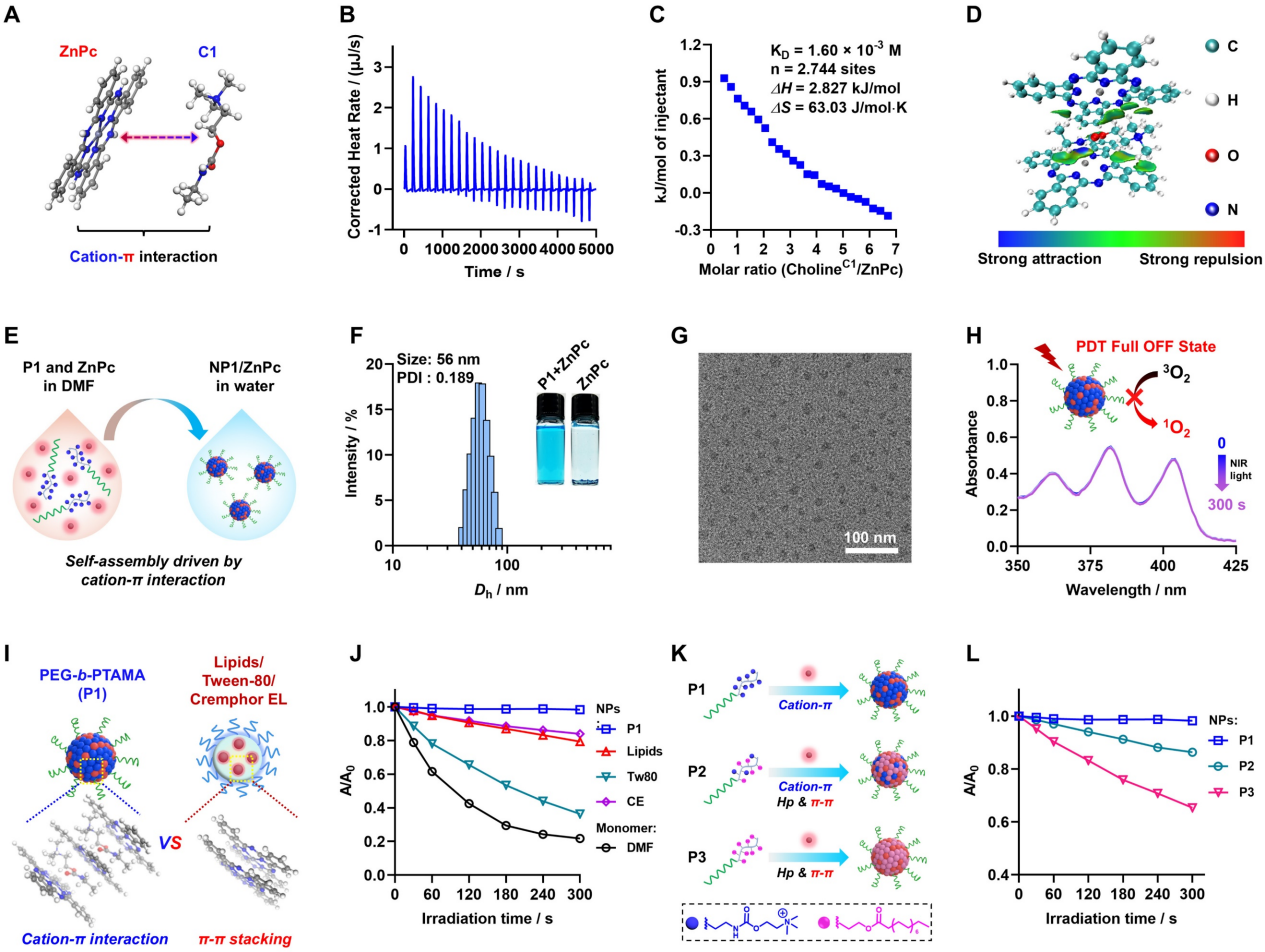
Characterization of cation-π interactions, self-assembly and PDT full OFF of NP1/ZnPc.** (**A) Illustration of the chemical structures of model cationic compound (C1) and π-structure compound (ZnPc). (B, C) ITC titration of the intermolecular interactions between C1 and ZnPc. (D) Independent gradient model (IGM) analysis shows strong cation-π interactions area between C1 and ZnPc moieties. (E) Schematic illustration of the self-assembly of NP1/ZnPc driven by cation-π interaction via nanoprecipitation method. (F) Size distribution of NP1/ZnPc. The insets clearly demonstrate the well-dispersed formation of nanoparticles by P1 + ZnPc, whereas ZnPc precipitation occurs in water. (G) TEM image of NP1/ZnPc. (H) Absorbance changes of ABDA (a probe for ^1^O_2_ detection) in the presence of NP1/ZnPc upon light irradiation (LED, 660 nm, 20 mW/cm^2^, 0-300 s) in aqueous solution. The results shown a PDT full OFF state of NP1/ZnPc. (I) Schematic illustration of different aggregation states of ZnPc inside nanostructures formulated by P1 in this work (cation-π interaction), and clinically used pharmaceutical drug carrier excipients (π-π stacking), including Lipids, Tween-80 (Tw80), and Cremophor EL (CE), respectively. (J) Plots of A/A_0_ ratio of ABDA vs irradiation time in the presence of ZnPc monomer (dissolved in DMF), and ZnPc assembles (NPs formulated by P1 or pharmaceutical excipients in water), respectively. (K) Schematic illustration of the manipulation of driven forces for self-assembly and regulation of the aggregation state of PS by modifying the side chains of the polymers. Specifically, P1 of cation-π interaction; P2 of cation-π interaction mixed with hydrophobic interaction (Hp) and π-π stacking; P3 of Hp and π-π stacking. (L) Plots of A/A_0_ ratio of ABDA vs light irradiation time in the presence of different ZnPc assembles (NPs formulated by P1 or control polymer P2, and P3, respectively). All the ZnPc formulas used for^ 1^O_2_ detection with ZnPc concentration of 20 μM. All the light irradiation using 660 nm LED, 20 mW/cm^2^.

To achieve the aforementioned design objectives, diblock copolymers comprising a hydrophilic polyethylene glycol (PEG) segment and a cationic choline-containing segment (PTAMA), termed PEG_45_-*b*-PTAMA_30_ (P1), were synthesized via reversible addition-fragmentation chain transfer (RAFT) polymerizations employing a PEG_45_-based macroRAFT agent (Figure S1-S2). Inspired by our previous work on poly(cation-π) micelles drug delivery platform 54, we first investigated the cation-π interactions between photosensitizers containing aromatic structure and cationic choline moieties (Figure 2A). As a demonstration, we employed a commercial near-infrared-excitable photosensitizer (ZnPc) to elucidate the molecular interactions between ZnPc and a C1 compound (mimics the choline moieties found in P1). As shown in the results of isothermal titration calorimetry (ITC) (Figure 2B-C and Figure S3), the favorable entropy (*ΔS*) indicates significant intermolecular interaction between C1 compound and ZnPc during the titration process. Specifically, the binding affinity (*K*_D_) was ascertained to be 1.60 × 10^-3^ M, with a binding number (n) of 2.744, which indicates that the stoichiometry of the C1/ZnPc complex is approximately 3:1. To further elucidate the molecular interactions between C1 and ZnPc, an independent gradient model (IGM) analysis was conducted. As shown in Figure 2D and Figure S4, interaction regions between the macrocycle π system of ZnPc and quaternary ammonium group of C1 can be clearly observed, indicating the formation of strong cation-π interactions within the ZnPc and C1 complex.

Encouraged by the above results, we aimed to harness cation-π interactions as a driving force for the construction of nanophotosensitizers. By employing an efficient nanoprecipitation method, the poly(cation-π) nanophotosensitizer (NP1/ZnPc) can be easily fabricated through the self-assembly of P1 polymer and ZnPc (Figure 1A and Figure 2E). As presented in Figure 2F and Figure S5-S6, the preparation conditions have been optimized to obtain the ideal NP1/ZnPc component, with a hydrodynamic diameter (*D*_h_) of approximately 56 nm and a uniform size distribution. The inset illustrates the well-dispersion of NP1/ZnPc (left) in aqueous medium, whereas a significant precipitation is observed in the absence of P1 during the same preparation process (right). Furthermore, the nanostructure of NP1/ZnPc was also evidenced by a transmission electron microscope (TEM) and Cryo-TEM (Figure 2G and Figure S7), which exhibited a typical spherical micellar morphology with highly uniform size. The encapsulation efficiency and loading capacity of ZnPc in NP1/ZnPc are 85.2% and 2.49%, respectively. Additionally, the elemental mapping images of NP1/ZnPc clearly revealed the presence of Zn, O, and S elements (Figure S8), thereby confirming the successful encapsulation of ZnPc by P1. Moreover, the results obtained from X-ray photoelectron spectroscopy (XPS) analysis (Figure S9) are highly consistent with those from element mapping images. Thus, the above findings provide robust evidence for the successful preparation of NP1/ZnPc nanoparticles. Importantly, given the high hydrophilicity of P1, we propose that strong cation-π interactions between the choline moieties in P1 and ZnPc facilitate the formation of the NP1/ZnPc (cation-π complex) core, while the hydrophilic PEG segments serve as a shell layer to stabilize the nanostructure (Figure 1A).

### Cation-π complexes induced complete OFF of PDT activity of poly(cation-π) nanophotosensitizers

Following the successful synthesis of the poly(cation-π) nanophotosensitizers (NP1/ZnPc), we proceeded to assess the PDT efficacy of NP1/ZnPc by utilizing 9,10-anthracenediyl-bis(methylene)dimalonic acid (ABDA) as a probe for ^1^O_2_ detection. Surprisingly, a complete OFF state of photoactivity was observed in NP1/ZnPc after 5 min irradiation with LED light (660 nm, 20 mW/cm^2^) (Figure 2H). Indeed, the complete OFF state of photosensitizers in non-tumor regions is highly advantageous, as it effectively mitigates side effects associated with undesired excitation and phototoxicity. To investigate the unique function of PDT OFF of poly(cation-π) nanophotosensitizers, various conventional nanophotosensitizers with diverse nanoformulations comprised of clinically used pharmaceutical adjuvants were fabricated. These included lipid (LNPs/ZnPc), Tween-80 (Tw80/ZnPc), and Cremophor EL (CE/ZnPc) formulations (Figure 2I and Figure S10). Subsequently, the ^1^O_2_ generation capability of the aforementioned conventional nanophotosensitizers was assessed. As shown in Figure 2J and Figure S11, all three nanophotosensitizers exhibit noticeable photoactivity, although their activity is somewhat reduced compared to that of free photosensitizers dissolved in solvent (ZnPc monomer in DMF). Specifically, the attenuation of photoactivity among these photosensitizers was ranked as follows: NP1/ZnPc > CE/ZnPc > LNPs/ZnPc > Tw80/ZnPc > ZnPc monomer. The assembly of ZnPc with these three conventional materials resulted in the aggregation of ZnPc, thereby enhancing the likelihood of π-π stacking, which is considered the primary factor contributing to the quenching of ZnPc's photoactivity 56. However, in contrast to the complete suppression of photoactivity in NP1/ZnPc, all the above conventional nanophotosensitizers exhibit an inability to achieve such suppression. Taking into account the notable difference in the driving force during the assembly process of these nanophotosensitizers (cation-π interactions for NP1/ZnPc, and hydrophobic interactions for CE/ZnPc, LNPs/ZnPc, and Tw80/ZnPc), preliminary analysis suggests that the cation-π complexes result in the complete suppression of the photoactivity within NP1/ZnPc.

Furthermore, to comprehensively investigate whether the complete OFF state of photosensitizers was induced by cation-π interactions, we manipulated the aggregation microenvironment of ZnPc by utilizing a combination of mixed cation-π interactions and hydrophobic & π-π stacking interactions at a molecular level. To this end, we designed and synthesized two additional control diblock copolymers, PEG_45_-*b*-P(PAMA_0.83_-*co*-TAMA_0.17_)_36_ (P2) and PEG_45_-*b*-PPAMA_33_ (P3), comprising long alkyl moieties (palmitic acid, PAMA) in different proportions (Figure S12-S15). In Figure 2K, P2 primarily comprised of a copolymerized segment comprising approximately 83% PAMA and 17% TAMA, which facilitates self-assembly into NP2/ZnPc through cation-π, hydrophobic, and π-π stacking interactions (Figure S16). On the other hand, P3 comprised a pure PA segment (100%) and self-assembled into NP3/ZnPc driven by hydrophobic and π-π stacking interactions. Interestingly, as the percentage of PAMA component increased, a significant decrease in the suppression of the photoactivity of nanophotosensitizers was observed in NP2/ZnPc and NP3/ZnPc compared to NP1/ZnPc, with the order of photoactivity being NP3/ZnPc > NP2/ZnPc > NP1/ZnPc (Figure 2L and Figure S17). The results neatly demonstrated that the cation-π interactions play a predominant role in the suppression of photoactivity, surpassing the contributions of hydrophobic and π-π stacking interactions typically employed in conventional nanophotosensitizers.

### Mechanism study and photosensitizer universality exploring for the PDT OFF in poly(cation-π) NPs

**Figure 3:**
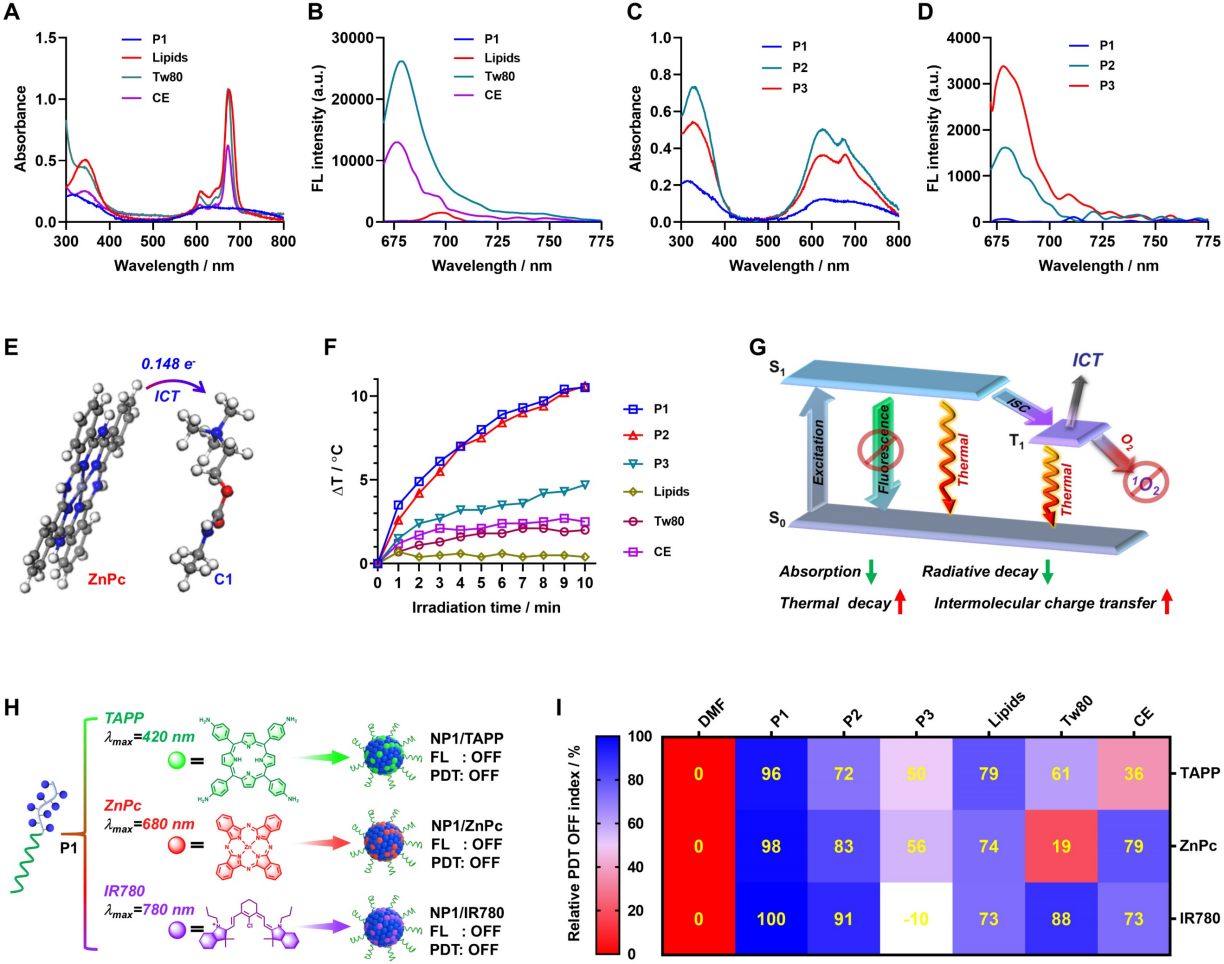
Mechanism study and photosensitizer universality exploring for the PDT OFF in poly(cation-π) NPs. (A) Absorption and (B) fluorescence spectra recorded for ZnPc assembles formulated by P1 or different pharmaceutical excipients, including Lipids, Tween-80 (Tw80), and Cremophor EL (CE), respectively. (C) Absorption and (D) fluorescence spectra recorded for ZnPc assembles formulated by P1 or control polymer (P2 and P3), respectively. (E) Schematic illustration of the intermolecular charge transfer (ICT) between ZnPc and C1. (F) Photothermal effect of various ZnPc assembles under 660 nm laser irradiation (1.0 W/cm^2^). All the ZnPc equivalent concentrations was 25 μM. (G) Jablonski diagram depicting the proposed mechanism of the full OFF PDT in NP1/ZnPc caused by cation-π complexes. (H) Schematic illustration of the self-assembly of three clinically used photosensitizers and P1. ZnPc, TAPP, and IR780 with the maximum excitation wavelength (λ_max_) of 420 nm, 680 nm, and 780 nm, respectively. (I) The relative PDT OFF index of ZnPc, TAPP, and IR780 assembles with different formulations, compared to the monomer PS dissolved in DMF. The calculation formulas of relative PDT OFF index were shown in supporting information Figure S33.

Theoretically, according to the Jablonski diagram, once PSs were excited, three energy dissipation pathways may be involved, including fluorescence emission, intersystem crossing (ISC) and thermal deactivation 57,58. Guided by the Jablonski diagram, we are dedicated to thoroughly exploring the underlying mechanism for full OFF state of NP1/ZnPc caused by cation-π complexes (Figure 1A). Firstly, the absorption and fluorescence spectra of all the aforementioned ZnPc nanoformulations were analyzed. In a comparative evaluation of various clinically used pharmaceutical adjuvants, NP1/ZnPc exhibited significantly lower absorption capability compared to LNPs/ZnPc, Tw80/ZnPc, and CE/ZnPc (Figure 3A). The order of absorption strength was observed as P1 < CE < Lipids ≈ Tw80, indicating the least favorable excitation state of ZnPc in NP1/ZnPc under the same light irradiation. Additionally, the fluorescence emission of NP1/ZnPc was completely OFF, while the other pharmaceutic adjuvant groups exhibited obvious fluorescence at 660 nm excitation wavelength (P1 < CE < Lipids ≈ Tw80) (Figure 3B). On the other hand, the ZnPc formulated with the two control polymers (P2 and P3) showed significantly increased absorption and fluorescence intensity (Figure 3C-D), which was consistent with the previously described photoactivity results.

Moreover, the intermolecular charge transfer (ICT) effect between the photosensitizer and cationic moiety was assessed through density functional theory (DFT) calculation. The results clearly indicated that 0.148 e^-^ were transferred from ZnPc to the cationic moiety through intermolecular charge transfer (ICT) (Figure 3E). Thus, it is reasonable to speculate that the formation of cation-π complex facilitated the intermolecular charge transfer from electron-rich ZnPc to the cationic moiety, which synergistically contributed to the OFF state of photoactivity in NP1/ZnPc. Furthermore, the complete OFF state of both photodynamic therapy (PDT) and fluorescence of NP1/ZnPc indicates that leveraging vibrational relaxation for heat generation may serve as a crucial dissipation pathway following the excitation of ZnPc. As expected, NP1/ZnPc in water exhibited a substantial increase in heat after exposure to 660 nm light (1 W/cm^2^) for 10 min (Figure 3F and Figure S18). In comparison, the temperature change (ΔT) of the other control ZnPc nanoformulations was significantly lower than that of NP1/ZnPc. The only exception was NP2/ZnPc, which is composed of mixed cation-π, hydrophobic and π-π stacking interactions, showed a similar ΔT mainly because of its much higher absorption capability compared to NP1/ZnPc. Overall, the full OFF state of NP1/ZnPc can be attributed to a multi-pathway process induced by cation-π complexes, including reduced absorption and radiative decay, as well as enhanced thermal decay and intermolecular charge transfer (Figure 3G).

Encouraged by the innovative mechanism of the PDT OFF of NP1/ZnPc, and considering that most photosensitizers possess aromatic π-structures, we envisioned developing a universal nanophotosensitizer based on cation-π interactions utilizing P1 polymer, which could be compatible with a variety of photosensitizers. As proof-of-concept, two commercially available and widely used photosensitizers with different maximum excitation wavelengths (λ_max_) were examined, including 5,10,15,20-tetrakis(4-aminophenyl)-21H,23H-porphine (TAPP, λ_max_ = 420 nm) and 2-[2-[2-chloro-3-[(1,3-dihydro-3,3-dimethyl-1-propyl-2H-indol-2-ylidene) ethylidene]-1-cyclohexen-1-yl]ethenyl]-3,3-dimethyl-1-propylindolium iodide (IR780, λ_max_ = 780 nm). As shown in Figure 3H and Figure S19-S22, the cation-π interactions played a key role in driving the self-assembly of P1 and PSs (TAPP and IR780) to form nanophotosensitizers, which were confirmed by ITC titration. DLS and TEM measurements validated the successful fabrication of NP1/TAPP and NP1/IR780. For comparison, a series of TAPP and IR780 nanoformulations were prepared as controls using the same method as described in ZnPc nanoformulations (Figure S23-S24). Expectedly, a decrease in absorption capacity was observed in both NP1/TAPP and NP1/IR780, which is consistent with the NP1/ZnPc (Figure S25-S28). Additionally, fluorescence measurements indicated the complete OFF state within both NP1/TAPP and NP1/IR780. Furthermore, the photoactivity of TAPP and IR780 nanoformulations was further measured using ABDA and 2,7-dichlorodihydrofluorescein (DCFH) probes, respectively.

Importantly, NP1/TAPP and NP1/IR780 effectively demonstrated a completely OFF state of PDT activity, compared to the control materials that exhibited significant ROS generation under light irradiation (Figure S29-S32). For a more visual and quantitative analysis, the relative PDT OFF index (RPOI) of each nanophotosensitizers was calculated (calculation formulas see Figure S33). As summarized in Figure 3I, all the poly(cation-π) NPs including NP1/ZnPc, NP1/TAPP, and NP1/IR780, possessed the highest RPOI values (close to 100%), while the other control groups exhibited comparatively lower PROI values. This finding highlights the remarkable universality and accessibility of the construction platform for activatable nanophotosensitizers (NP1/PSs), which demonstrate superior PDT OFF functions.

### Switch-ON the photoactivity of tumor-targeted TNP1/ZnPc in tumor cells

**Figure 4:**
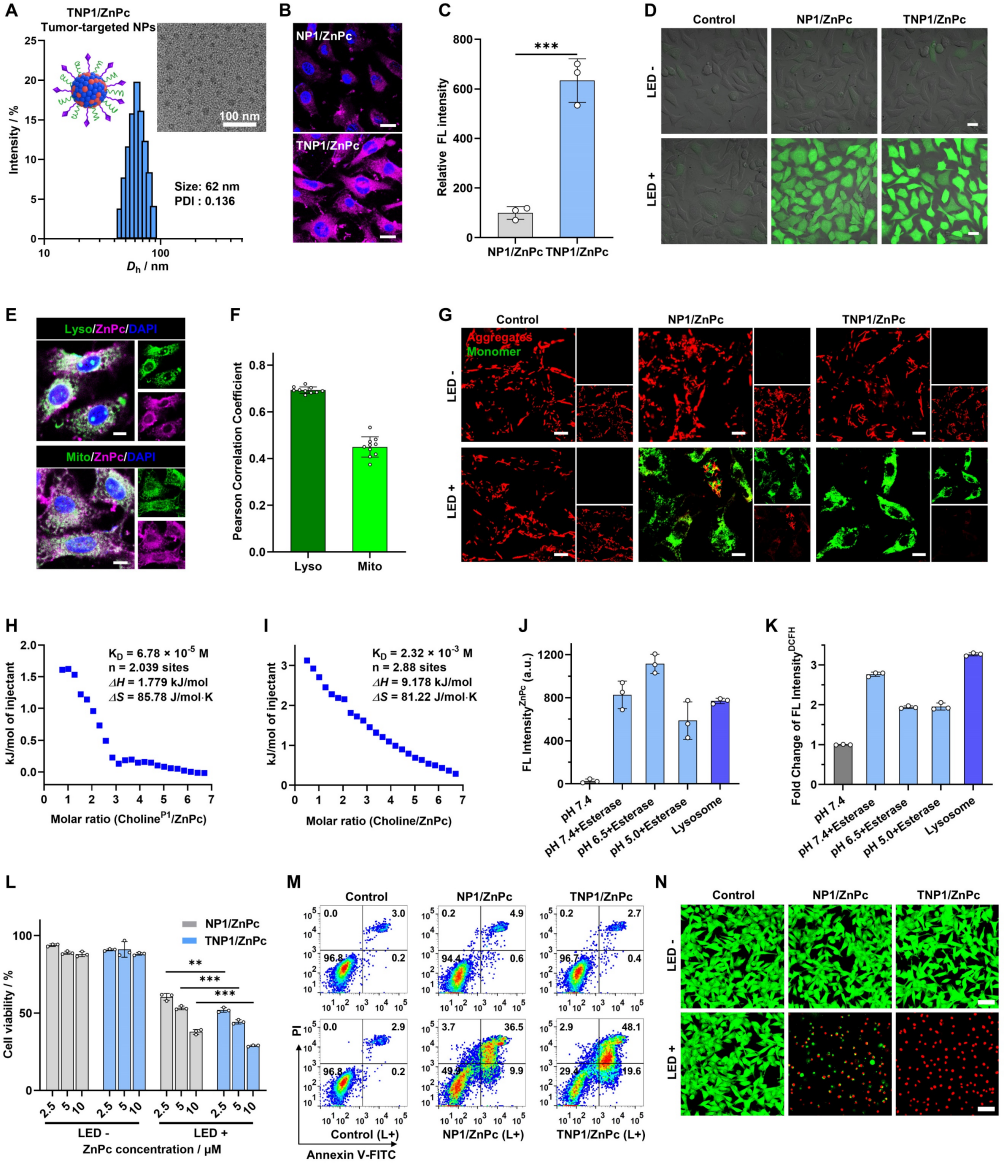
PDT activity recovery of tumor targeted TNP1/ZnPc in tumor cells. (A) Size distribution and the TEM image (inset) of TNP1/ZnPc. (B) CLSM images and (C) statistical analysis of fluorescence intensity of 4T1 cells incubated with NP1/ZnPc or TNP1/ZnPc for 2 h, respectively. [ZnPc] = 5 μM. Scale bar, 20 μm. Mean ± SD, n = 3, ***P < 0.001. (D) CLSM images of DCFH-DA incubated 4T1 cells after treatments with NP1/ZnPc or TNP1/ZnPc for 2 h in dark or upon LED light irradiation (660 nm, 20 mW/cm^2^, 5 min). Scale bar, 20 μm. (E) CLSM images and (F) statistical analysis of the colocalization of TNP1/ZnPc with lysosome and mitochondria in 4T1 cells. Scale bars, 10 μm. (G) JC-1 assay for 4T1 cells after various treatments. Scale bar = 10 μm. (H, I) ITC titration of the intermolecular interactions recorded for (H) choline moieties in P1 (Choline^P1^) with ZnPc, and (I) choline with ZnPc, respectively. (J) Changes of fluorescence emission intensities of ZnPc in TNP1/ZnPc before and after incubation with esterase at different pH, or extracted lysosome, for 2 h at 37 °C, respectively. (K) ROS generation of TNP1/ZnPc before and after incubation with esterase at different pH or extracted lysosome upon LED light irradiation (660 nm, 20 mW/cm^2^, 5 min). DCFH was used as ROS probe. (L) Cell viability of 4T1 cells incubated with NP1/ZnPc or TNP1/ZnPc under different concentrations and without or with LED light irradiation (660 nm, 20 mW/cm^2^, 5 min). Mean ± SD, n = 3, **P < 0.01, ***P < 0.001. (M) Cell apoptosis of 4T1 cells analyzed by flow cytometer with Annexin V-FITC/PI double staining after various treatments. (N) Live-dead staining assay for 4T1 cells after various treatments. Scale bar, 50 μm.

Inspired by the exceptional performance of completely switching OFF photoactivity within poly(cation-π) nanophotosensitizers (like NP1/ZnPc), we are keenly investigating and characterizing the profile of the photoactivity in tumor cells. To this end, a tumor-targeted formulation of poly(cation-π) nanophotosensitizers, namely TNP1/ZnPc, was constructed to improve the tumor-targeted delivery efficiency (Figure 1A). Firstly, a tumor-targeted and negatively charged polymer, PSPMA_8_-cRGD, was synthetized and characterized (Figure S34-S36). Subsequently, the tumor-targeted TNP1/ZnPc was engineered using a similar procedure as NP1/ZnPc, employing the co-assembly of ZnPc, P1, and PSPMA8-cRGD in water. During the self-assembly process, PSPMA_8_-cRGD were encapsulated based on polyionic complex interactions with cRGD moieties anchored to TNP1/ZnPc. DLS and TEM revealed the successful preparation of TNP1/ZnPc with slightly change in both size (62 nm) and Zeta potential (+30.5 mV) compared with NP1/ZnPc (56 nm and +39.6 mV) (Figure 4A and Figure S37). Notably, the encapsulation efficiency (79.3%) and loading capacity (2.12%) of ZnPc in TNP1/ZnPc are only slightly lower than those in NP1/ZnPc. TNP1/ZnPc exhibited well-dispersed in aqueous media with no significant change in size for up to one month when stored at 4 °C, thereby demonstrating remarkable structural stability (Figure S38). Significantly, TNP1/ZnPc has been demonstrated to possess a complete OFF function for photoactivity, which is in alignment with the properties of NP1/ZnPc. (Figure S39). Besides, the complete OFF function of TNP1/ZnPc exhibited high stability during one month of storge at 4 °C (Figure S40). Moreover, the structure integrity and OFF function stability were also evaluated in water under different pH (5.0, 6.5, and 7.4) as well as in culture medium (DMEM). Taken altogether, TNP1/ZnPc was stable in size and OFF function in water under different pH for one month. However, only slight size change of TNP1/ZnPc could be observed upon exposure to DMEM compared to the water environment, while the OFF function still kept stable (Figure S41).

Next, we evaluated the tumor-targeted endocytosis of TNP1/ZnPc in 4T1 cells that overexpress the αvβ3 receptor. As shown in Figure 4B-C, the fluorescence signal of ZnPc was significantly stronger in the TNP1/ZnPc-treated 4T1 cells, approximately 6.3 times higher than that of the NP1/ZnPc-treated group. The results indicated highly effective tumor-targeted delivery of TNP1/ZnPc. To further verify the tumor-targeted property of TNP1/ZnPc mediated by cRGD, cell uptake study of TNP1/ZnPc in cell lines with different expression levels of integrin was conducted. The result clearly showed the excellent endocytosis of TNP1/ZnPc in cell lines with high expressed integrin (A549, B16F10 and 4T1), while less uptake was occurred in cell line with low expressed integrin (MCF-7) (Figure S42). More importantly, considering that the fluorescence of ZnPc was in the OFF state in both TNP1/ZnPc and NP1/ZnPc, the detected fluorescence signal indicated the restoration of the photosensitizers' fluorescence (termed FL Switch-On) in the tumor cells. Meanwhile, the ability of TNP1/ZnPc to generate reactive oxygen species (ROS) in tumor cells was investigated using the DCFH-DA probe (Figure 4D). Along with the Switch-On of FL of TNP1/ZnPc, a significant amount of ROS was produced after a 2-hour incubation upon LED light irradiation, which demonstrated the recovery of photoactivity of TNP1/ZnPc (termed PDT Switch-On) in tumor cells. By contrast, 4T1 cells incubated with NP1/ZnPc or TNP1/ZnPc without irradiation showed very weak signals of ROS. Similarly, PDT Switch-On ability of NP1/TAPP or NP1/IR780 were also confirmed in 4T1 cells upon light irradiation compared with non-irradiation group (Figure S43).

Moreover, co-localization experiments using Lyso-tracker and Mito-tracker revealed that the fluorescence signals of TNP1/ZnPc were predominantly localized in the lysosomes, with some signals also observed in the mitochondria following 2 hours of incubation. (Figure 4E). The correlation coefficient between TNP1/ZnPc and lysosomes was 0.69, whereas it was 0.45 for mitochondria (Figure 4F). Taking into account the pronounced switching on photoactivity of tumor-targeted TNP1/ZnPc, the mitochondria damage in tumor cells upon irradiation was examined. JC-1 staining was performed to assess the effect of ROS generated by TNP1/ZnPc on mitochondrial membrane potential (MMP) (Figure 4G). The results demonstrated that 4T1 cells treated with TNP1/ZnPc and exposed to LED irradiation exhibited a significant decrease in the J-aggregate signal (red) and an increase in the J-monomer signal (green). This suggests effective MMP depolarization, indicating a potential enhancement of programmed cell death and tumor suppression effects through the PDT treatment with TNP1/ZnPc.

Based on the above results, we determined that the endocytosis of TNP1/ZnPc mainly mediated by the lysosome pathway, which is accompanied by the spontaneous Switch-On of PDT activity in tumor cells and mitochondria. Subsequently, the underlying mechanism for the recovery of photoactivity of TNP1/ZnPc in tumor cells was explored. Certainly, linkers featuring enzyme-cleavable ester or carbamate bonds are typically engineered for selective cleavage within the tumor microenvironment, such as endosomes and lysosomes, which are rich in esterase and various hydrolases 59,60. Herein, cationic choline moieties in the P1 polymer were covalently linked to polymer backbone *via* ester and carbamate bonds within TNP1/ZnPc, which can be efficiently degraded in endosome and lysosome. Therefore, we hypothesized that the degradation of TNP1/ZnPc occurs within lysosomes, resulting in the transformation of cation-π interactions from a strong polyvalent form to a less stable one, thereby weakening the cation-π complexes and facilitating the recovery of the FL and PDT activity of ZnPc (Figure 1B). Furthermore, the degradation of P1 skeleton resulted in the exposure of a great deal of primary amines (Figure 1B), which finally contributed to the lysosomal escape through the “proton sponge effect”.

To investigate the proposed mechanism of the intracellular Switch-On of photoactivity in TNP1/ZnPc, ITC titrations was employed to quantitatively evaluate the strength of cation-π interactions between ZnPc and P1 or small molecular choline (mimicking the hydrolysis products). As shown in Figure 4H-I and Figure S44-S45, the dissociation constant (*K_D_*) of P1 with ZnPc was determined to be 6.78 × 10^-5^ M, which is approximately two orders of magnitude lower than that of choline with ZnPc (*K_D_* = 2.32 × 10^-3^ M). This indicated the superior binding affinity of the cation-π interaction between P1 and ZnPc. The results revealed that the structural degradation of TNP1/ZnPc in lysosomes weakens the cation-π interactions between ZnPc and cationic moieties. Furthermore, the correlation between the degradation of TNP1/ZnPc triggered by esterase or extracted lysosomes and the recovery of FL and photoactivity was also investigated (Figure 4J-K and Figure S46-S48). After incubation with esterase at different pH or extracted lysosomes at 37 ºC, the fluorescence and photoactivity of TNP1/ZnPc was dramatically increased. All the above results of extracellular and intracellular experiments demonstrated that two novel poly(cation-π) nanophotosensitizers, NP1/ZnPc and TNP1/ZnPc, have been successfully developed, which not only possess the complete OFF state of photoactivity upon self-assembly in aqueous environments, but also feature the spontaneously Switch-On capability after targeted uptake by tumor cells.

### *In vitro* PDT performance of TNP1/ZnPc

Motivated by the efficient intracellular ROS generation of TNP1/ZnPc, MTT assay was utilized to assess the *in vitro* phototherapeutic efficacy toward tumor cells. After being irradiated with a 660 nm LED light (20 mW/cm^2^) for 5 minutes, the viability of 4T1 cells significantly decreased in a dose-dependent manner in the presence of TNP1/ZnPc (Figure 4L). Notably, benefiting from the efficient tumor-targeted endocytosis, TNP1/ZnPc showed enhanced cytotoxicity toward 4T1 cells under irradiation compared to NP1/ZnPc without targeting function. Furthermore, the results of cell apoptosis assays showed that 4T1 cells treated with TNP1/ZnPc in conjunction with light irradiation induced a higher apoptosis rate (67.7%) (early and late apoptosis) compared to NP1/ZnPc (46.4%) (Figure 4M). Finally, Calcein AM/PI double staining experiments were performed to further validate the *in vitro* cytotoxicity of nanophotosensitizers on 4T1 cells. As shown in Figure 4N, TNP1/ZnPc exhibited the highest proportion of dead cells (red fluorescence), indicating the effective PDT efficiency of TNP1/ZnPc on 4T1 cells. Importantly, both TNP1/ZnPc and NP1/ZnPc demonstrated negligible cytotoxicity towards 4T1 cells under dark conditions, highlighting the excellent biocompatibility of poly(cation-π) nanophotosensitizers (Figure 4L-N).

### *In vivo* imaging and PDT efficacy of TNP1/ZnPc

**Figure 5:**
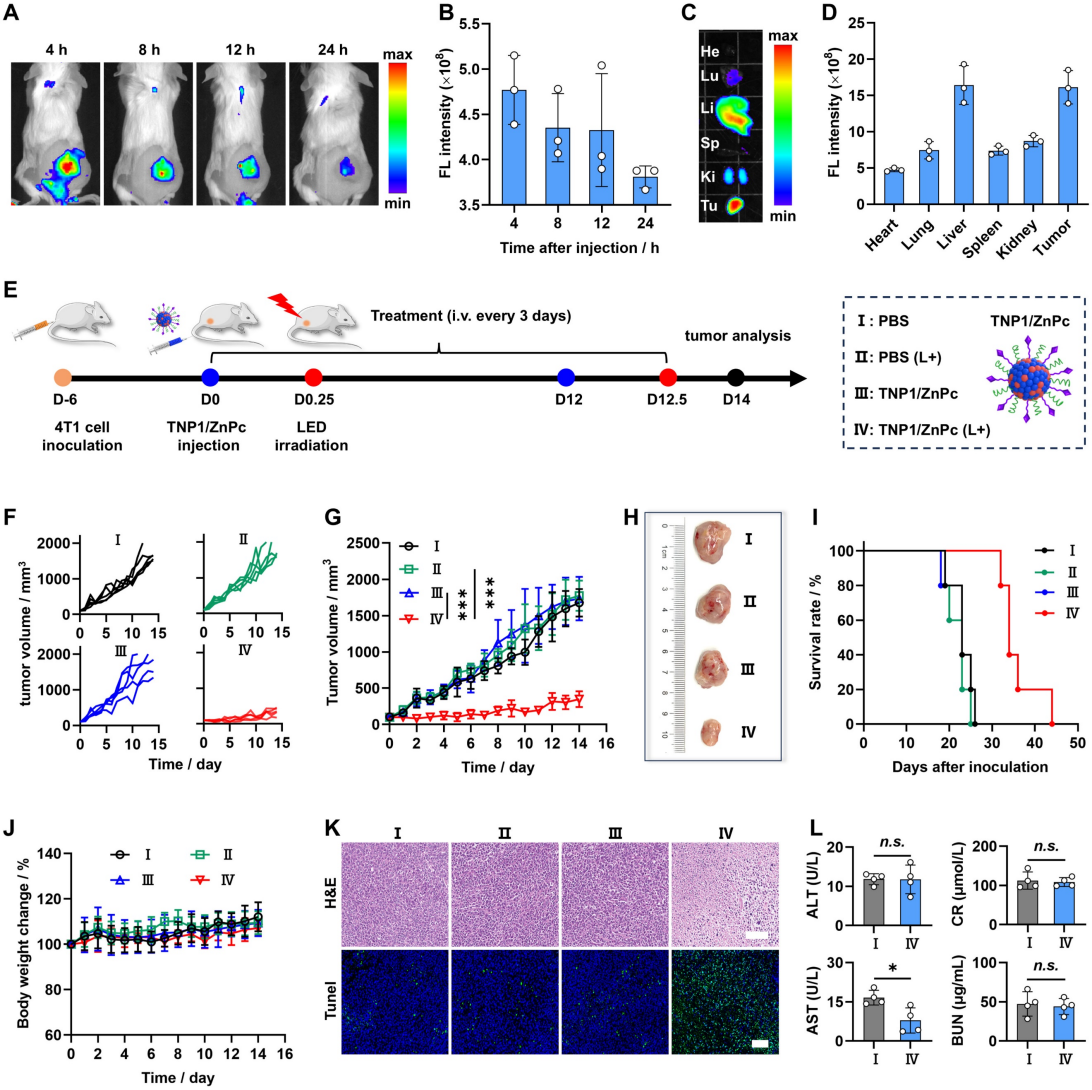
Tumor-targeted PDT performance of TNP1/ZnPc in tumor-bearing mice. (A) *In vivo* imaging of 4T1 tumor-bearing BALB/c mice and (B) statistical analysis of the quantitative fluorescence signals in the tumors after intravenous injection of Cy5 labelled TNP1/ZnPc@Cy5. Mean ± SD,* n* = 3. (C) *Ex vivo* imaging and (D) quantification of fluorescence signals in tumors and the other major organs at 24 h post-injection of TNP1/ZnPc@Cy5. Mean ± SD,* n* = 3. (E) Schematic illustration of the schedule for subcutaneous 4T1 tumor model inoculation and the therapeutic processes. L+: LED light irradiation (660 nm, 20 mW/cm^2^, 5 min). (F, G) Tumor growth curves of mice after different treatments, including PBS, PBS (L+), TNP1/ZnPc, and TNP1/ZnPc (L+), respectively. *n* = 5, ***P < 0.001. (H) Representative photographs of the excised tumors after 14 days various treatments. (I) The survival analysis of mice following various treatments. (J) Body weight changes of mice during various treatments. (K) H*&*E staining and TUNEL assays of the excised tumor tissues resected from mice after 14 days various treatments. Scale bar, 100 μm. (L) Biochemical analysis for liver and kidney functions of mice after 14 days treatments. Mean ± SD, *n* = 4, *n.s.* means no significance, *P < 0.05.

To evaluate the tumor enrichment and biodistribution of TNP1/ZnPc, Cy5-labeled TNP1/ZnPc (TNP1/ZnPc@Cy5) was prepared and characterized (Figure S49). As shown in Figure 5A-B and Figure S50-S51, following the intravenous (*i.v.*) injection of TNP1/ZnPc@Cy5, the fluorescence (FL) signals at the tumor sites peaked at 4 hours post-injection and subsequently declined over time. Compared with TNP1/ZnPc@Cy5, the non-targeted group (NP1/ZnPc) showed relatively lower tumor enrichment at each time point. The *ex vivo* FL images of the resected 4T1 tumors and major organs revealed that the 4T1 tumors and liver possessed the relative high FL at 24 h post-injection of TNP1/ZnPc@Cy5, whereas other organs displayed significantly lower FL than that in tumors (Figures 5C-D and S50B). These results indicated the remarkable tumor-targeting capability of the poly(cation-π) nanophotosensitizers.

Encouraged by the excellent *in vitro* PDT performance, *in vivo* PDT efficacy of TNP1/ZnPc was further evaluated. The procedure of the animal experiments was shown in Figure 5E. The mice were randomly grouped and treated with PBS, PBS+LED, TNP1/ZnPc, and TNP1/ZnPc+LED separately, when the tumor volume reached 100 ~ 150 mm^3^ (n = 6). As shown in Figure 5F-G, administration of TNP1/ZnPc+LED significantly suppressed the tumor growth during 14 days treatment, whereas PBS+LED and TNP1/ZnPc groups showed no obviously inhibition effect on 4T1 tumor when compared to the PBS group. Additionally, images of representative tumor tissues in each group also confirmed the significant anti-tumor efficacy of TNP1/ZnPc+LED (Figure 5H). The study found that the survival rate of 4T1 tumor-bearing mice treated with TNP1/ZnPc+LED was effectively prolonged (Figure 5I). Significantly, there were no obvious body weight change in all groups during the treatment, indicating the favorable biosafety of TNP1/ZnPc (Figure 5J). Furthermore, the therapeutic effects were also evaluated by tumor hematoxylin and eosin (H&E) and TUNEL staining after therapy (Figure 5K). Representative images of H&E staining revealed the largest area of tumor necrosis in TNP1/ZnPc+LED treated group in comparison to other groups. Meanwhile, the largest amount of apoptosis cells was also observed in TNP1/ZnPc+LED treated group through TUNEL staining. On the other hand, during the therapy process, TNP1/ZnPc caused no hepatic and renal damages (Figure 5L). Finally, the H&E staining analysis of major organs were further confirmed the great biocompatibility of TNP1/ZnPc indicating by the absence of pathological abnormalities (Figure S52). Moreover, the relationship between irradiation dose and PDT efficiency was elucidated. As expected, it can be inferred from Figure S53-S54 that a higher irradiation dose (660 nm, 50 mW/cm^2^, 10 min) used in PDT treatment resulted in stronger tumor growth inhibition compared to a lower irradiation dose (660 nm, 20 mW/cm^2^, 5 min). Along with the above exploration, the skin phototoxicity of TNP1/ZnPc was initially evaluated during the above experiment. Detailly, mice in PBS+LED group and TNP1/ZnPc+LED groups (including high irradiation dose group and low irradiation dose group) were subjected to additional sunlight exposure during the PDT process. In line with our expectations, no visible skin damage was observed across all groups during the PDT process, thereby providing preliminary validation of the skin phototoxicity-free nature of TNP1/ZnPc (Figure S55).

### *In vivo* skin phototoxicity-free of TNP1/ZnPc

**Figure 6:**
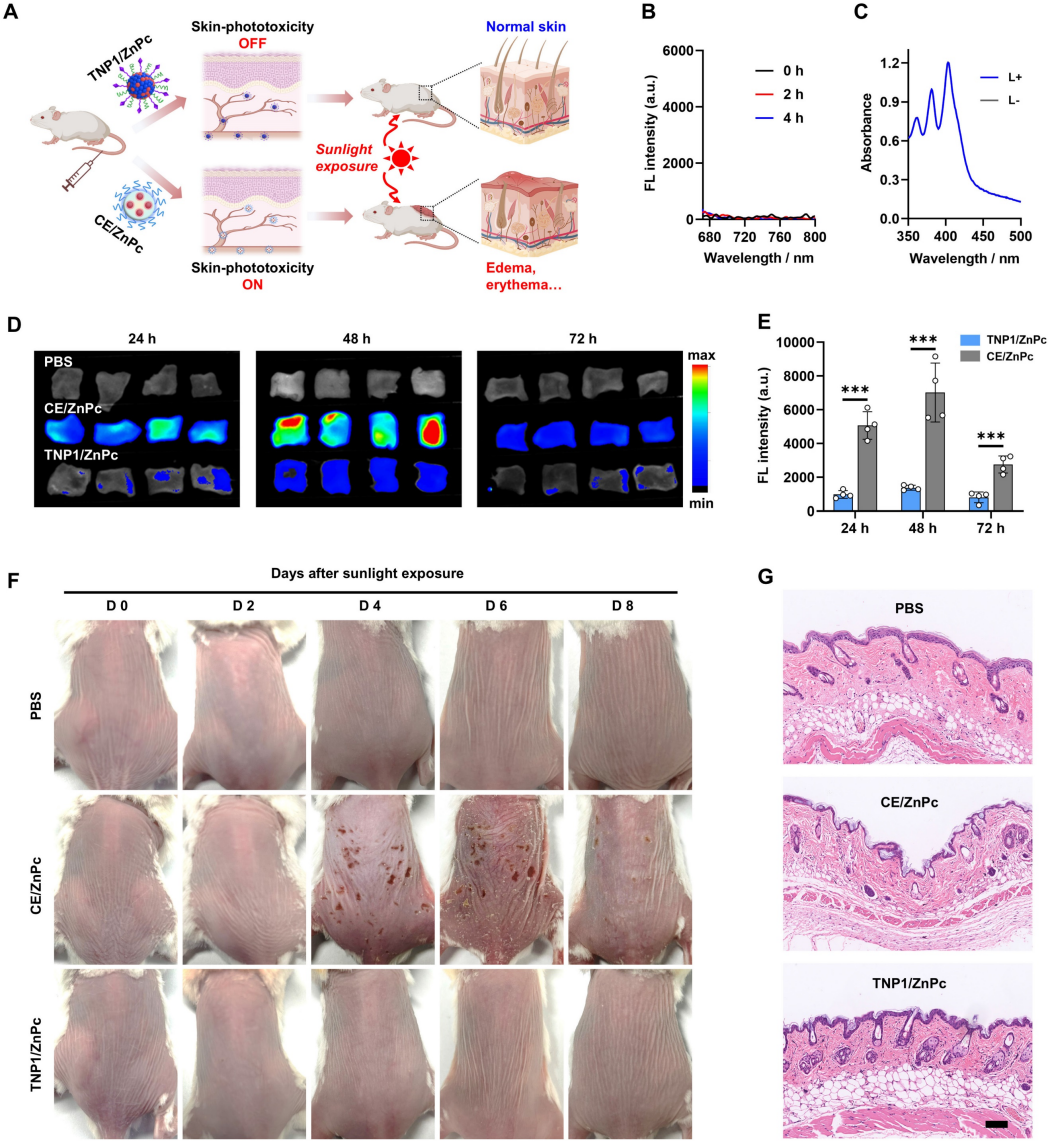
Skin safety and phototoxicity-free of TNP1/ZnPc. (A) Schematic illustration of the skin-phototoxicity OFF of TNP1/ZnPc *in vivo* after intravenous injection, compared to the significant skin-phototoxicity of traditional NPs (e.g. CE/ZnPc formulated by Cremphor EL). (B) Fluorescence spectra recorded for TNP1/ZnPc in water with 10% FBS after incubation for 0, 2, 4 h, respectively. (C) Absorbance changes of ABDA in the presence of TNP1/ZnPc before and after light irradiation (LED, 660 nm, 20 mW/cm^2^, 5 min). TNP1/ZnPc was pre-incubated in water with 10% FBS for 4 h. (D) *Ex vivo* images and (E) quantification of fluorescence signals of skin tissues excised from the back of mice at 24, 48, 72 h post-intravenous injection of TNP1/ZnPc or CE/ZnPc, respectively. Mean ± SD,* n* = 4, ***P < 0.001. (F) Photographs of mice after intravenous injection of PBS as negative control, TNP1/ZnPc, or CE/ZnPc followed by simulated sunlight exposure for 0-8 days. (G) The corresponding H&E staining of skin slice at 4 days post-light exposure. Scale bars, 100 μm.

Building upon the aforementioned results, a novel tumor-targeted activatable nanophotosensitizer has been successfully developed, featuring a complete OFF state of photoactivity in aqueous media and a spontaneous Switch-ON capability of PDT activity within tumor cells. Therefore, we hypothesize that this nanophotosensitizer possesses the potential to circumvent the adverse effects triggered by sunlight exposure, particularly photosensitization and severe skin damage. To evaluate the skin phototoxicity of TNP1/ZnPc *in vivo*, CE/ZnPc, a conventional nanophotosensitizer consisting of Cremphor EL (CE) (a pharmaceutical compound commonly used in clinic) was chosen as a control in the experiments (Figure 6A). Firstly, the complete OFF state of TNP1/ZnPc in blood circulation was demonstrated. The fluorescence and ROS generation of TNP1/ZnPc were determined after incubated with 10% fetal bovine serum (FBS) for 4 h at 37 ºC. As shown in Figure 6B-C and S56, the complete OFF state of both the FL and photoactivity suggested the excellent structure stability of TNP1/ZnPc during blood circulation. Meanwhile, the photoactivity transformation of TNP1/ZnPc in immune cells with phagocytosis function in the blood circulation was also evaluated. To some extent, TNP1/ZnPc can be uptake by RAW264.7 and CTLL-2 cells. Notably, in conjunction with the inefficient photoactivity recovery, TNP1/ZnPc showed poor ROS generation ability upon light irradiation in RAW264.7 and CTLL-2 cells when compared with that in tumor cells (Figure S57-S58). Then, normal mice were treated with TNP1/ZnPc and CE/ZnPc at the same ZnPc concentrations *via* tail vein injection, respectively. Mice in each group were sacrificed at 24, 48, and 72 h post-injection and the skin tissues of mice were collected for *ex vivo* FL imaging. The results revealed that the skin tissues of mice treated with TNP1/ZnPc exhibited a significantly weaker FL signal compared to those treated with CE/ZnPc (Figure 6D). The FL signal of each group peaked at 48 hours post-injection and gradually declined thereafter. Quantitative assessment of FL intensity indicated that the CE/ZnPc-treated group exhibited FL signals 5.1, 5.1, and 3.4 times higher than those of the TNP1/ZnPc-treated group at 24, 48, and 72 hours post-injection, respectively (Figure 6E). The results from both *in vitro* and *in vivo* experiments clearly indicated that TNP1/ZnPc exhibits a stable OFF state of photoactivity. This finding is of considerable significance as it may provide a potential solution to the issue of phototoxicity induced by photosensitizers* in vivo*.

To further evaluate the skin phototoxicity of nanophotosensitizers following sunlight exposure, mice were administered a single intravenous dose of either TNP1/ZnPc or CE/ZnPc, ensuring that the ZnPc concentrations in both formulations were equivalent. After being anesthetized and exposed to a solar simulator for 30 minutes (100 mW/cm^2^) at 0.5 days post-injection, mice in each group were kept in the dark and photographed every 2 days. Clearly, no skin phototoxicity was observed in the TNP1/ZnPc treated group during the experimental period compared to the PBS control group (Figure 6F). In contrast, mice subjected to treatment with CE/ZnPc endured severe skin erythema and edema on their backs, which progressed to scarring by day 4. Moreover, the skin damage in the CE/ZnPc group could not fully recover within 8 days post-irradiation. Additionally, H&E staining was conducted to assess the histological alterations of the skin (Figure 6G). It was evident that the skin of TNP1/ZnPc treated mice remained intact following light irradiation, similar to that of mice treated with PBS. Strikingly, the groups treated with CE/ZnPc displayed a markedly thinner epidermis and a reduction of hair follicles number when compared with both the PBS and TNP1/ZnPc treated groups. Furthermore, pronounced necrosis was evident within the dermal region. The above results unequivocally confirmed that TNP1/ZnPc did not induce skin phototoxicity following irradiation. Finally, the hematological parameters of the mice with TNP1/ZnPc treatments at 8 days post-irradiation remained in the normal range compared to those in the PBS group, suggesting the good biocompatibility of the nanophotosensitizers (Figure S59). Overall, it is critically important to engineer activatable nanophotosensitizers capable of a complete OFF state to ensure both efficacy and safety in photodynamic therapy applications. The poly(cation-π) nanophotosensitizers, designed innovatively through cation-π interactions, possess significant potential to surmount this challenge.

## Conclusion

We herein developed a novel tumor-targeted activatable nanophotosensitizer platform utilizing cation-π interactions for high anti-tumor efficacy and completely avoiding skin phototoxicity. The robust polyvalent cation-π interactions between the cationic choline-containing polymer (P1) and aromatic photosensitizers (PSs) facilitate the self-assembly of P1 and PSs, resulting in the formation of stable poly(cation-π) nanophotosensitizers designated as TNP1/PSs. Interestingly, a complete OFF state of photoactivity was observed in TNP1/PSs. Mechanism studies revealed that a multi-pathway process induced by cation-π complexes, including reduced absorption and radiative decay, as well as enhanced thermal decay and intermolecular charge transfer (ICT), collectively contributes to the complete PDT OFF state of TNP1/PSs. The universality of the PDT OFF function to various photosensitizers across the exciting wavelength range of 400 nm to 800 nm were also demonstrated, employing TAPP, ZnPc, and IR780 as examples. Upon targeting tumor cells, TNP1/ZnPc was effectively endocytosed and predominantly passing through the lysosomes. Then, the hydrolysis of the ester bonds and carbamates within TNP1/ZnPc results in the degradation of the cationic polymers, which causes the transformation of strong polyvalent cation-π interactions to weak cation-π interactions. Along with this process, the photoactivity of ZnPc spontaneously switched on in tumor cells. Both *in vitro* and *in vivo* studies demonstrated the high anti-tumor efficacy following mild near-infrared irradiation. Importantly, benefitting from the full OFF function of the nanophotosensitizer in the bloodstream prior to entering tumor cells, the intravenous treatment using TNP1/ZnPc did not cause noticeable skin phototoxicity or any other adverse side effects, even when exposed to simulated sunlight irradiation. In summary, we successfully developed a tumor-targeted, activatable, and skin-phototoxicity-free nanophotosensitizer based on cation-π interactions facilitated self-assembly and the OFF-ON switching of photoactivity. This work provides valuable insights into the design of safe and effective nanophotosensitizer for cancer treatment.

## Materials and Methods

### Materials, cell lines, and animals

N-Hydroxysulfosuccinimide sodium salt (Sulfo-NHS) and esterase from porcine liver were purchased from Meryer Chemical Technology Co., Ltd. Triethylamine (TEA) were purchased from Energy chemical. 2-Hydroxyethyl methacrylate and dibutyltin dilaurate (DBTL) were purchased from Sigma-Aldrich. Palmitic acid (PA) was purchased from Ailan Chemical Technology Co., Ltd. Methacrylic acid 3-sulfopropyl ester potassium salt (SPMA) and 1-(3-Dimethylaminopropyl)-3-ethylcarbodiimide hydrochloride (EDCl), 9,10-anthracenediyl-bis(methylene)dimalonic acid (ABDA) were purchased from Bide Pharmatech Ltd. Tumor-targeting peptide cRGDFK was purchased from Nanjing Peptide Biotech Ltd. Purified 2,2-Azoisobutyronitrile (AIBN) was obtained by recrystallization from 95% ethanol. Model choline derivate (C1), choline-containing monomer (TAMA), RAFT agent (CTA-COOH) and poly (ethylene oxide) based macromolecular RAFT agent (PEO_45_-CTA) was synthesized according to previous literature reports 61,62. Palmitic acid-containing monomer (PAMA) was designed and synthesized by our research group. Choline Iodide, N, N'-Dicyclohexylcarbodiimide (DCC), 4-Dimethylaminopyridine (DMAP), Tween-80 (Tw80), and Cremophor EL (CE) were purchased from Shanghai Macklin Biochemical Technology Co., Ltd. Cholesterol, zinc phthalocyanine (ZnPc), 2,7-dichlorodihydrofluorescein (DCFH) and MTT was purchased from Shanghai yuanye BioTechnology Co., Ltd. DSPC was purchased from Ponsure biological. mPEG-DSPE was purchased from Tanshtech Co. Ltd. 5,10,15,20-Tetrakis(4-aminophenyl) porphyrin (TAPP) and IR780 were purchased from Shanghai Acmec Biochemical Technology Co., Ltd. DiSulfo Cyanine5 carboxylic acid (Disulfo-Cy5-COOH) was purchased from CONFLUORE BioTechnology Co., Ltd. 4',6-diamidino-2-phenylindole (DAPI) was purchased from Leagene Biotechnology. Live/dead cell double staining kit and Annexin V-FITC/PI apoptosis detection kit were purchased from KeyGEN BioTECH Co., Ltd. Lyso-tracker green, Mito-tracker green probe, ROS assays Kit and mitochondrial membrane potential assay kit with JC-1 was purchased from Beyotime Biotechnology Co., Ltd. Lysosome extraction kit was purchased from BestBio Technology Co., Ltd. Dulbecco's modified Eagle medium (DMEM) and 0.25% Trypsin-EDTA solution were purchased from Gibco. Phosphate buffered saline (PBS). Fetal bovine serum (FBS) was purchased from ExCell Bio.

4T1 cell line, MCF-7 cell line, A549 cell line, B16F10 cell line, RAW264.7 cell line, and CTLL-2 cell line were obtained from American Type Culture Collection (ATCC). 4T1 cell line, A549 cell line, B16F10 cell line, and RAW264.7 cell line were cultured in DMEM medium supplemented with 10% fetal bovine serum (FBS) and 1% penicillin/streptomycin. MCF-7 cell line and CTLL-2 cell line were cultured in RPMI 1640 medium supplemented with 10% fetal bovine serum (FBS) and 1% penicillin/streptomycin.

Female Balb/c mice (5-week-old) were purchased from Center for Experimental Animals, Southern Medical University. All mice were housed in pathogen-free conditions and kept in a room with controlled temperature (~22 ºC) and humidity (45%-60%) under 12 h light/dark cycle. All animal experiment were carried out under the guideline approved by the Institutional Animal Care and Use Committee (IACUC) of Southern Medical University (2021178).

### Characterization

All ^1^H NMR spectra were recorded on a 400 MHz Bruker instrument. All UV/vis absorbance spectra were obtained using Evolution 300 instrument (ThermoFisher Scientific). Fluorescence measurements were performed on a LUMINA Fluorescence Spectrometer (Thermo Scientific). Dynamic laser light scattering (DLS) and zeta potential measurements were performed using a Malvern Zetasizer Nano ZS instrument. Transmission electron microscope (TEM) images were obtained using a FEI Tecnai 12 electron microscope. The samples for TEM observations were prepared by dropping 10 µL of aqueous dispersion of the self-assembled aggregates onto copper grids coated with thin films of Formvar and carbon. Cryo-TEM images were obtained using a FEI Talos F200c electron microscope. Elemental mapping images were obtained using a JEOL JEM-F200 electron microscope. X-ray photoelectron spectroscopy (XPS) spectra were obtained using K-Alpha+ instrument (Thermo Scientific). Isothermal titration calorimetry (ITC) data were obtained using a Nano-ITC LV instrument (TA Instruments). MTT assays were conducted on a Synergy H1 microplate reader (BioTek). Fluorescence images were taken by Nikon A1 confocal laser scanning microscopy (CLSM) (Nikon Instruments). Apoptosis assays were conducted on a flow cytometry (BD Fortesa). *In vivo* biodistribution assays were conducted on a IVIS Lumina II (PerkinElmer).

### Synthesis of choline contained diblock copolymer, PEG-b-PTAMA_n_ (P1)

P1 polymer was synthesized through reversible addition-fragmentation chain transfer (RAFT) polymerization. Briefly, choline-containing monomer (TAMA) (400 mg, 1.03 mmol, 30 equiv.), PEG_45_-CTA (78 mg, 0.034 mmol, 1.0 equiv.), and AIBN (1.5 mg, 0.009 mmol, 0.26 equiv.) were dissolved in DMF (500 μL). The above mixtures were transferred into a reaction tube and degassed by freeze-pump-thaw cycles for three times and finally sealed under vacuum. The reaction tube was then immersed into an oil bath at 65 ºC. After stirring for 12 h, the reaction tube was quenched into liquid nitrogen and opened. The reaction mixture was diluted with 3 mL water and dialyzed against deionized water for 8 h with replacing fresh deionized water every 2 h, to remove organic solvent and water-soluble impurities. Finally, the solution in dialysis bag was lyophilized and the P1 polymer was obtained as a light-yellow solid (379 mg, yield: 79%). The total degree of polymerization, DP, of PTAMA_n_ block was determined to be 30 by ^1^H-NMR analysis. Thus, the polymer was denoted as PEO_45_-*b*-PTAMA_30_, (Figure S2).

### Synthesis of amphiphilic diblock copolymer as control, PEG_45_-b-P(PAMA_x_-co-TAMA_y_) (P2)

Palmitic acid-containing monomer (PAMA) (400 mg, 1.05 mmol, 35 equiv.), TAMA (117 mg, 0.3 mmol, 10 equiv.), PEG_45_-CTA (68 mg, 0.03 mmol, 1.0 equiv.), and AIBN (1.5 mg, 0.009 mmol, 0.3 equiv.) were dissolved in DMF (600 μL), and then were transferred into a reaction tube followed by degassed via three freeze-pump-thaw cycles and finally sealed under vacuum. The tube was immersed into an oil bath at 65 ºC. After stirring for 12 h, the reaction tube was quenched into liquid nitrogen and opened. The reaction mixture was precipitated into an excess of n-hexane. Then the residue was dissolved in DCM and precipitated into an excess of n-hexane again. The above dissolution-precipitation cycle was carried out for three times and the polymer was obtained as yellow viscous solid (355 mg, yield: 76%). The degree of polymerization, DP, of P(PAMA_x_-*co*-TAMA_y_) block was determined to be x = 30 and y = 6, by ^1^H-NMR analysis. Thus, the diblock polymer was denoted as PEG_45_-*b*-P(PAMA_0.83_-*co*-TAMA_0.17_)_36._ (Figure S14).

### Synthesis of amphiphilic diblock polymer as control, PEG_45_-b-PPAMA_z_ (P3)

PAMA (400 mg, 1.05 mmol, 35 equiv.), PEG_45_-CTA (68 mg, 0.03 mmol, 1.0 equiv.) and AIBN (1.5 mg, 0.009 mmol, 0.3 equiv.) were dissolved in DMF (500 μL) and then were transferred into a reaction tube followed by degassed via three freeze-pump-thaw cycles and finally sealed under vacuum. The tube was immersed into an oil bath at 65 ºC. After stirring for 10 h, the reaction tube was quenched into liquid nitrogen and opened. The reaction mixture was precipitated into an excess of n-hexane. Then the residue was dissolved in DCM and precipitated into an excess of n-hexane again. The above dissolution-precipitation cycle was carried out for three times and the polymer was obtained as the yellow oil (363 mg, yield: 62%). The degree of polymerization, DP, of PPAMA_z_ block was determined to be 33, by ^1^H-NMR analysis. Thus, the diblock copolymer was denoted as PEG_45_-*b*-PPAMA_33._ (Figure S15).

### Synthesis of negative-charge polymer PSPMA_m_-COOH and cRGD conjugated PSPMA_m_-cRGD

SPMA (300 mg, 1.22 mmol, 15 equiv.), CTA-COOH (23 mg, 0.08 mmol, 1.0 equiv.), and AIBN (1.5 mg, 0.009 mmol, 0.11 equiv.) were dispersed in DMF (500 μL), and then were transferred into a reaction tube followed by degassed via three freeze-pump-thaw cycles and finally sealed under vacuum. The tube was immersed into an oil bath at 65 ºC. After stirring for 16 h, the reaction tube was quenched into liquid nitrogen and opened. The reaction mixture was diluted with 3 mL water and dialyzed against deionized water for 8 h with replacing fresh deionized water every 2 h, to remove organic solvent and water-soluble impurities. Finally, the solution in dialysis bag was lyophilized and the PSPMA_m_-COOH polymer was obtained as light-yellow solid (203 mg, yield: 63%). The total degree of polymerization, DP, of PSPMA_m_ block was determined to be 8 by ^1^H-NMR analysis. Thus, the polymer was denoted as PSPMA_8_-COOH (Figure S35).

Next, for cRGD conjugation, PSPMA_8_-COOH (30 mg, 13.2 μmol), Sulfo-NHS (18.2 mg, 84 μmol), and EDCl (48 mg, 250 μmol) were dissolved in 2 mL MES buffer and stirred for 4 h at room temperature. cRGD (9.7 mg, 16 μmol), was then added into the above mixture and keep stirring for 12 h at room temperature. The product was purified by dialysis against deionized water and finally lyophilized. The successful conjugation of cRGD was characterized by ^1^H-NMR analysis. The polymer was denoted as PSPMA_8_-cRGD (Figure S36).

### Computational simulation

The Independent Gradient Model (IGM) 63. analysis was performed through Multiwfn (version 3.8) software package 64 and visualized by VMD (version 1.9.3) 65. The ground state geometry was optimized using DFT calculations. All calculations were performed with the Gaussian 16 package 66 using the hybrid B3LYP functionals 67,68 and the 6-31G* basis set. Grimme's D3BJ dispersion correction 69,70 was used to improve calculation accuracy.

### Preparation of nanophotosensitizer based on cation-π interactions

Typical procedures employed for photosensitizer (ZnPc, TAPP, and IR780)-loaded cation-π nanoparticles (NP1/ZnPc, NP1/TAPP, and NP1/IR780) was as follows. Taking NP1/ZnPc as an example, 10 mg P1 was dissolved in 1 mL DMF solution containing 0.3 mg ZnPc, and then the above solution was added into 9 mL deionized water in one shot under magnetic stirring (1500 rpm). After being stirred for 10 min at room temperature, the suspension was transferred into a dialysis bag (MWCO: 3,500 Da) and dialyzed against water (2 L) for 12 h, with replacing fresh water every 3 h, to remove organic solvent. NP1/TAPP and NP1/IR780 were also prepared using the similar procedures as NP1/ZnPc.

### Preparation of nanophotosensitizer based on traditional formulations

For photosensitizer-loaded lipid-based nanoparticles preparation (LNPs/ZnPc, LNPs/TAPP, and LNPs/IR780), typical procedures were employed as follows. Taking LNPs/ZnPc as an example, 2 mg DSPE-PEG, 6 mg DSPC, and 2 mg cholesterol were dissolved in 1 mL DMF solution containing 0.3 mg ZnPc, and then the above solution was added into 9 mL deionized water in one shot under magnetic stirring (1500 rpm). After being stirred for 10 min in room temperature, the suspension was transferred into a dialysis bag (MWCO: 3,500 Da) and dialyzed against deionized water (2 L) for 12 h, with replacing fresh deionized water every 3 h, to remove organic solvent. Similar procedures as LNPs/ZnPc were employed for preparation of LNPs/TAPP and LNPs/IR780.

Cremophor EL and Tween-80 are the two typical kinds of surfactant widely used in clinical practice. Herein, the method used to prepare photosensitizer-loaded Cremophor EL (CE/ZnPc) or Tween-80 (Tw80/ZnPc) was similar to the preparation of FDA-approved Paclitaxel injection (Taxol^®^) 71,72. Briefly, 5 mg ZnPc, 527 mg Cremophor EL and 49.7 (v/v) % ethanol were well-mixed under vortex, and then the mixture was diluted to certain concentration for further experiments with deionized water. CE/TAPP, CE/IR780, Tw80/ZnPc, Tw80/TAPP and Tw80/IR780 were also prepared using the similar procedures as CE/ZnPc.

### Preparation of nanophotosensitizer based on cation-π and/or hydrophobic interactions

The photosensitizer (ZnPc, TAPP, and IR780)-loaded nanoparticles based on cation-π and hydrophobic interactions (NP2/ZnPc, NP2/TAPP, and NP2/IR780) using P2 polymer, and only hydrophobic interactions (NP3/ZnPc, NP3/TAPP, and NP3/IR780) using P3 polymer, were prepared using the similar procedures as NP1/ZnPc.

### Measurement of loading efficiency and loading content

Taking NP1/ZnPc for instance, after dialyzed against distill water, NP1/ZnPc suspension (0.5 mL) was freeze-drying and redissolved in 2.5 mL DMF. The ZnPc concentration were determined using UV-vis spectroscopy based on the standard calibration curves. The loading content and loading efficacy were calculated according to the following formulas: Loading content (%) = weight of ZnPc in nanoparticle/weight of ZnPc loaded nanoparticle × 100%. Loading efficiency (%) = weight of ZnPc in nanoparticle/weight of ZnPc in feed × 100%. The loading efficiency and loading content of other nanoformulations were determined using the similar methods as described above.

### ITC determination for cation-π interactions between choline derivates and photosensitizers

Nano-ITC system equipped with a 500 μL sample cell and a 50 μL injection syringe was used to study the cation-π interactions between C1 compound and ZnPc. C1 compound was synthesized to simulate the choline moieties in P1. The experiments were carried out at 298.15 K, and the data was corrected for the heat of dilution of the titrants. The samples were degassed with the degassing station (TA Instruments). Detailly, the syringe consisted of 34 mM of C1 solution (DMF, titrant) and the cell consisted of 0.66 mM of photosensitizers (DMF, titrant). The titration consisted of 25 injections of 1.9 μL of the syringe solution at the time interval of 200 s in all the ITC measurements. For all titrations, heats of dilution were subtracted and curves were fitted with an Independence binding model.

Besides, cation-π interactions between photosensitizers and P1 or choline were also conducted using the similar procedures as above. Differently, the syringe consisted of 1.13 mM of P1 solution (DMF, titrant) or 34 mM of choline solution (DMF, titrant), respectively.

### *In vitro* detection of ^1^O_2_ generation

The ^1^O_2_ generation of free photosensitizer or photosensitizer-loaded nanoparticles under light irradiation was detected by a singlet oxygen detection probe, ABDA. Briefly, for ZnPc, 2 mL of ZnPc formulations solution (including ZnPc 20 μM, free ZnPc in DMF or nanoparticles in water) containing 50 μM ABDA were irradiated with an LED light (660 nm, 20 mW/cm^2^), and the absorption of ABDA was recorded at timed intervals. Similar procedures were used in measurement of TAPP formulations, and 530 nm LED light (20 mW/cm^2^) was used for TAPP excitation.

Differently, DCFH was employed for ^1^O_2_ generation detection of IR780 formulations. Specifically, 2 mL of IR780 formulations solution (including IR780 20 μM, free IR780 in DMF or nanoparticles in water) containing 10 μM DCFH were irradiated with laser (808 nm, 20 mW/cm^2^), and the fluorescence intensity of DCFH was recorded at timed intervals.

The relative PDT OFF index was calculated as the followed formula (A) for ZnPc and TAPP, and (B) for IR780, respectively:

**A**



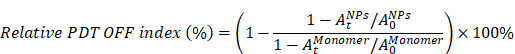



**B**



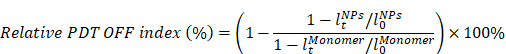



### Photothermal performance of ZnPc loaded nanoparticles with different formulations

The thermal effects of ZnPc loaded nanoparticles with different formulations, including NP1/ZnPc, NP2/ZnPc, NP3/ZnPc, LNPs/ZnPc, Tw80/ZnPc, and CE/ZnPc were inspected respectively. The above ZnPc formulations with the specified concentrations (12, 25, and 50 μM) were continuously exposed to 660 nm laser (1 W/cm^2^). The temperature was continuously monitored using TiS20 Thermal imager (FLUKE) and recorded every 1 min. Meanwhile, pure deionized water under the same condition served as the control groups and calculated the temperature difference (ΔT).

### Preparation of tumor-targeted ZnPc-loaded cation-π nanoparticles (TNP1/ZnPc)

10 mg P1 was dissolved in 1 mL DMF solution containing 0.3 mg ZnPc, and then the above solution was added into 9 mL deionized water containing 1 mg cRGD-PSPMA_8_ in one shot under magnetic stirring (1500 rpm). After stirred for 10 min at room temperature, the suspension was transferred into a dialysis bag (MWCO: 3,500 Da) and dialyzed against water (2 L) for 12 h, with replacing fresh water every 3 h, to remove organic solvent.

### CLSM image of cell internalization of NP1/ZnPc and TNP1/ZnPc

Briefly, 4T1 cells were cultured in a 35 mm confocal dish and treated with NP1/ZnPc or TNP1/ZnPc (including ZnPc 5 µM) for 2 h, respectively. The supernatant was removed and then the cells were washed with PBS for three times. The cells were fixed with 4% paraformaldehyde and stained with DAPI. Finally, fluorescent images were captured by CLSM. The fluorescent intensity of each group was calculated by Image J software.

Besides, tumor cells with different expression levels of integrin receptor (MCF-7, A549 and B16F10) were used to evaluate the cRGD mediated uptake behavior. MCF-7, A549 and B16F10 cells were treated following the same treatment as described above. Then, the fluorescent images were captured by CLSM.

To explore the phagocytosis of TNP1/ZnPc by immune cells, cell uptake experiments were also performed on RAW264.7 and CTLL-2 cells. RAW264.7 and CTLL-2 cells were both accepted the same treatment as described in that of 4T1 cells. The fluorescent images of RAW264.7 and CTLL-2 cells were captured by CLSM after the same treatment as described above.

### Intracellular ROS generation measurement of NP1/ZnPc and TNP1/ZnPc

The intracellular ROS generation of NP1/ZnPc or TNP1/ZnPc under light irradiation was detected by DCFH-DA. Briefly, 4T1 cells were seeded on a 35 mm confocal dish (4 × 10^5^ cells per well) and cultured overnight for adherence. The cells were incubated with NP1/ZnPc or TNP1/ZnPc (including ZnPc 5 μM) for 2 h. And then, the supernatant was removed, followed by addition of DCFH-DA (10 μM). After 30 min incubation, the cells were irradiated with LED light (660 nm, 20 mW/cm^2^) for 5 min, and then cells were washed twice with PBS, and imaged by CLSM. ROS generation without light irradiation was measured for comparison. Cells without treatment were served as control.

To explore the photoactivity recovery of TNP1/ZnPc in immune cells, RAW264.7 and CTLL-2 cells were incubated with TNP1/ZnPc (including ZnPc 5 μM) for 2 h. And then, the supernatant was removed, followed by addition of DCFH-DA (10 μM). After 30 min incubation, the cells were irradiated with LED light (660 nm, 20 mW/cm^2^) for 5 min, and then cells were washed twice with PBS, and imaged by CLSM.

### MTT assay

Intracellular PDT performance of NP1/ZnPc and TNP1/ZnPc on 4T1 cells was estimated by classical MTT assay. Briefly, 4T1 cells were seeded on two 96-well plates at 4000 cells/well. After 24 h incubation at 37 °C with 5% CO_2_ humidified atmosphere, the cultured medium in each well were removed and the cells were treated with NP1/ZnPc or TNP1/ZnPc at various ZnPc concentrations for 2 h in dark. One of the plates was irradiated with LED light (660 nm, 20 mW/cm^2^) for 5 min. After that, the supernatant of both plates was removed and the cells were washed with PBS twice, followed by replacing with 200 μL fresh complete cultured medium. After being incubated for another 22 h in dark, 20 μL MTT solution (5 mg/mL in PBS) was added to each well and further incubated for 4 h in the incubator. The supernatant in each well was carefully removed and 150 μL DMSO was added to each well, the 96-well plate was shaken for 15 min and then the absorbance of each well was measured by a microplate reader at 570 nm. Cells without treatment were served as control.

### Live-dead staining

For live/dead staining assay, 4T1 cells were cultured on 35 mm confocal dish and followed by the same treatment procedure as described above (including ZnPc 5 μM). After that, the supernatant was removed and the cells were washed with PBS for three times. Then, cells were incubated with Calcein-AM/PI staining solution at 37 °C for 20 min according to the manufacturer's protocol. After washed with PBS for three times to remove the dye outside the cells completely, the cells were imaged with confocal laser scanning microscopy. Cells without treatment were served as control.

### Cell apoptosis assay

For cell apoptosis assay, 4T1 cells were cultured in 12-well plate and incubated for 24 h, followed by the same treatment procedure as described above (including ZnPc 5 μM). After that, the cells were collected and resuspended in 500 μL binding buffer and stained with Annexin V-FITC and PI for 15 min at room temperature in dark. Finally, samples were detected by flow cytometry. Apoptosis without light irradiation was measured for comparison. Cells without treatment were served as control.

### Subcellular localization of TNP1/ZnPc

4T1 cells were seeded on 35 mm confocal dish at a density of 4 × 10^5^cells per well overnight and then cultured with TNP1/ZnPc (including ZnPc 5 μM) for 2 h. And then, the supernatant was removed and the cells were washed with PBS for three times and separately stained with Lyso-Tracker green or Mito-Tracker green for 15 min. After washing with PBS for twice, the cells were imaged by CLSM. The Pearson's R values were generated and analyzed.

### Mitochondrial membrane potential assessment (JC-1 staining)

For JC-1 staining assay, 4T1 cells were cultured on 35 mm confocal dish and followed by the same treatment procedures as described in live-dead staining experiments. After various treatments, the supernatants were removed and the cells were washed with PBS for three times. Afterward, the cells were stained with JC-1 in fresh medium for 15 min according to the manufacturer's protocol, followed by washing 3 times with PBS. Then, fluorescence images of the cells were captured by CLSM. The cells without light irradiation were measured for comparison. Cells without treatment were served as control.

### Mechanism study on the photoactivity recovery of TNP1/ZnPc in tumor cells

Briefly, 2 mL TNP1/ZnPc (including ZnPc 20 μM) was incubated with porcine liver esterase (100 U/mL) at pH 7.4, 6.5 or 5.0 or extracted lysosome (37 °C) in distilled water. Lysosomes were extracted according to manufacturer's protocol of lysosome extraction kit. At determined time intervals, the fluorescence emission spectrum of the treated TNP1/ZnPc was recorded (λ_ex_ = 660 nm). Meanwhile, the ROS generation ability of the treated TNP1/ZnPc was also detected using the DCFH probe after irradiated with an LED light (660 nm, 20 mW/cm^2^, 5 min) as described above.

### *In vivo* biodistribution of TNP1/ZnPc

Firstly, fluorescent probe (diSulfo-Cy5 carboxylic acid) labeled TNP1/ZnPc (TNP1/ZnPc@Cy5) was prepared following the similar method described above. Briefly, 10 mg P1 and 0.3 mg ZnPc were dissolved in 1.0 mL DMF. Then the above mixture was added into 9 mL deionized water containing 0.2 mg diSulfo-Cy5 carboxylic acid and 0.1 mg cRGD-PSPMA_8_ in one shot under magnetic stirring (1500 rpm). After being stirred for 10 min at room temperature, the suspension was dialyzed against deionized water (2 L) for 12 h, with replacing fresh PBS every 3 h, to remove organic solvent, unloaded diSulfo-Cy5 carboxylic acid. DiSulfo-Cy5 carboxylic acid labeled NP1/ZnPc (NP1/ZnPc@Cy5) was prepared following the similar method as described above.

To establish 4T1 tumor-bearing mice model, 4T1 cells suspension (3 × 10^6^ cells in 100 μL PBS) was subcutaneously injected into the right hind limb of each mouse. When the tumor volumes approached ~100 mm^3^, female 4T1 tumor-bearing mice were treated with 200 μL TNP1/ZnPc@Cy5 or NP1/ZnPc@Cy5 suspension by tail vein injection at a dose corresponding to 0.2 mg/kg of diSulfo-Cy5 carboxylic acid (n = 3). The tumor accumulation of TNP1/ZnPc@Cy5 or NP1/ZnPc@Cy5 was recorded at 0.5, 1, 2, 4, 8, 12, and 24 h post-injection using an* in vivo* imaging system (excitation at 630 nm and emission at 700 nm). For *ex vivo* imaging, the mice were euthanized and the tumor mass and major organs (heart, lung, liver, spleen, and kidney) were collected at 24 h post-injection and imaged. Fluorescence intensity data were quantified using Living Image software 4.5.

### *In vivo* antitumor efficacy of TNP1/ZnPc

4T1 tumor-bearing mice model was established as described above. Female 4T1 tumor-bearing mice were treated when the tumor volumes approached ~100 mm^3^. The 4T1 tumor-bearing mice were randomly distributed into four groups (n = 6): PBS, PBS (L+) (light irradiation), TNP1/ZnPc, and TNP1/ZnPc (L+) (light irradiation), respectively. Mice in groups of TNP1/ZnPc and TNP1/ZnPc (L+) were intravenously injected at an equivalent dose of 0.6 μmol/kg ZnPc once every 3 days for 5 times, respectively. Mice in groups of PBS (L+) and TNP1/ZnPc (L+) were anaesthetized and irradiated with LED light (660 nm, 20 mW/cm^2^, 5 min) at 0.25 d post-injection. Tumor volumes and body weight of each animal were measured during the experiment periods. Tumor volume (mm^3^) = width^2^ × length/2. At the end of the experiments, blood samples of each mouse were collected for biochemical analysis. At day 14, all the animals were euthanized and tumors, major organs were collected for further study.

In order to evaluate the skin phototoxicity of TNP1/ZnPc during PDT process and effect of irradiation dose on PDT outcome, female 4T1 tumor-bearing mice were randomly distributed into five groups (n = 5): PBS, PBS (660 nm, 20 mW/cm^2^, 5 min), TNP1/ZnPc, TNP1/ZnPc (660 nm, 20 mW/cm^2^, 5 min), and TNP1/ZnPc (660 nm, 50 mW/cm^2^, 10 min), respectively. Mice in groups of TNP1/ZnPc, TNP1/ZnPc (660 nm, 20 mW/cm^2^, 5 min), and TNP1/ZnPc (660 nm, 50 mW/cm^2^, 10 min) were intravenously injected at an equivalent dose of 0.6 μmol/kg ZnPc once every 3 days for 5 times, respectively. Mice in groups of PBS (660 nm, 20 mW/cm^2^, 5 min), TNP1/ZnPc (660 nm, 20 mW/cm^2^, 5 min), and TNP1/ZnPc (660 nm, 50 mW/cm^2^, 10 min), were anaesthetized and irradiated with LED light at 0.25 d post-injection followed by additional exposure to a solar stimulator for 30 min (100 mW/cm^2^) at 0.5 d post-injection. Tumor volumes and body weight of each animal were measured during the experiment periods. Tumor volume (mm^3^) = width^2^ × length/2. Skin images of mice in all groups were pictured every 2 days by digital camera.

### Serum stability

In order to evaluate the “OFF” state of TNP1/ZnPc in blood circulation, TNP1/ZnPc was mixed with fetal bovine serum (FBS) and incubated for 0, 2, and 4 h at 37 ºC, respectively. Then, 2 mL of the above mixture (including ZnPc 20 μM) containing 50 μM ABDA were irradiated with an LED light (660 nm, 20 mW/cm^2^, 5 min), and the absorption of ABDA was recorded. Additionally, the fluorescence spectra of the above mixture were also recorded at different incubation time.

### *In vivo* activity of photosensitizer in skin

To study the safety and phototoxicity-free of TNP1/ZnPc in skin tissue, healthy female BALB/c mice were sacrificed at different timepoints (24 h, 48 h, and 72 h) post-intravenous injection of TNP1/ZnPc or CE/ZnPc (ZnPc equiv. dose: 0.6 μmol/kg) (n = 9). Skin tissues in back of each mouse were carefully harvested and imaged (excitation at 630 nm and emission at 700 nm). Fluorescence signal of skin tissues was quantified. Mice treated with PBS were served as control.

### *In vivo* skin phototoxicity assay

Healthy female BALB/c mice with hair removal were randomly divided into 3 groups (n = 6) and then intravenously injected with PBS, TNP1/ZnPc, or CE/ZnPc (ZnPc equiv. dose: 0.6 μmol/kg) according to the corresponding groups. Mice in each group were anaesthetized and exposed to a solar stimulator for 30 min (100 mW/cm^2^) at 0.5 d post-injection. After that, mice of all groups were kept in dark and pictured every 2 days by digital camera. Skin tissues were collected from the back of mice at 4 d post-injection. H&E staining of skin tissues was performed for the skin damage evaluation.

### Statistics analysis

All experiments were repeated at least 3 times. Data are represented as mean ± SD. Statistical analysis were carried out with GraphPad Prism version 8.0.2 software. Statistical significance was calculated using unpaired Student's *t*-test.

## Supplementary Material

Supplementary figures.

## Abbreviations

PSs: photosensitizers
TNP1/PSs: photosensitizers-loaded tumor-targeted activatable nanophotosensitizers
NP1/PSs: photosensitizers-loaded activatable nanophotosensitizers
ZnPc: zinc phthalocyanine
cRGD-PSPMA: cRGD conjugated sulfated polymers
CE: cremphor el
ITC: isothermal titration calorimetry
IGM: independent gradient model
ABDA: 9,10-anthracenediyl-bis(methylene)dimalonic acid
Tw80: tween-80
LNPs: lipid nanoparticles
ISC: intersystem crossing
ICT: intermolecular charge transfer
TAPP: 5,10,15,20-tetrakis(4-aminophenyl)-21H,23H-porphine
IR780: 2-[2-[2-chloro-3-[(1,3-dihydro-3,3-dimethyl-1-propyl-2H-indol-2-ylidene) ethylidene]-1-cyclohexen-1-yl]ethenyl]-3,3-dimethyl-1-propylindolium iodide
DCFH: 2,7-dichlorodihydrofluorescein
RPOI: relative PDT OFF index
H&E: hematoxylin and eosin staining
