# Supplementary material for: A general strategy towards activatable nanophotosensitizer for phototoxicity-free photodynamic therapy
Source: Theranostics. 2025 Jan 1;15(3):943–64. doi: 10.7150/thno.100597 (PMC11700870; doi:10.7150/thno.100597)
Supplement: Supplementary file 1 — Supplementary figures. [file thnov15p0943s1.pdf]

Supporting Information

A general strategy towards activatable nanophotosensitizer for phototoxicity-free photodynamic therapy

Guozhu Tan[1,2], Qinjie Zhong[2], Jibin Zhang[2], Peiyi He[2], Xiaoxi Zhao[2], Guifeng Miao[2,3], Yafei Xu[1], and Xiaorui Wang[2,3*]

[1]Department of Orthopaedics and Traumatology, The Seventh Affiliated Hospital, Southern Medical University 528000, Foshan, Guangdong Province, China

[2]Biomaterials Research Center, School of Biomedical Engineering, Southern Medical University 510515, Guangzhou, Guangdong Province, China

[3]Department of Cardiovascular Surgery, Zhujiang Hospital, Southern Medical University, 510280, Guangzhou, Guangdong Province, China

*Corresponding authors: wxr114@smu.edu.cn

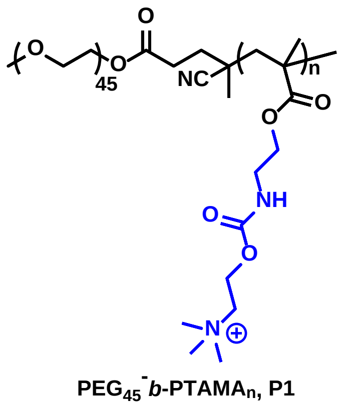

Figure S1. Synthetic route employed for the preparation of the cationic block copolymer containing choline moieties, PEG_{45}-*b*-$PTAMA_n$ (P1), using RAFT polymerization.

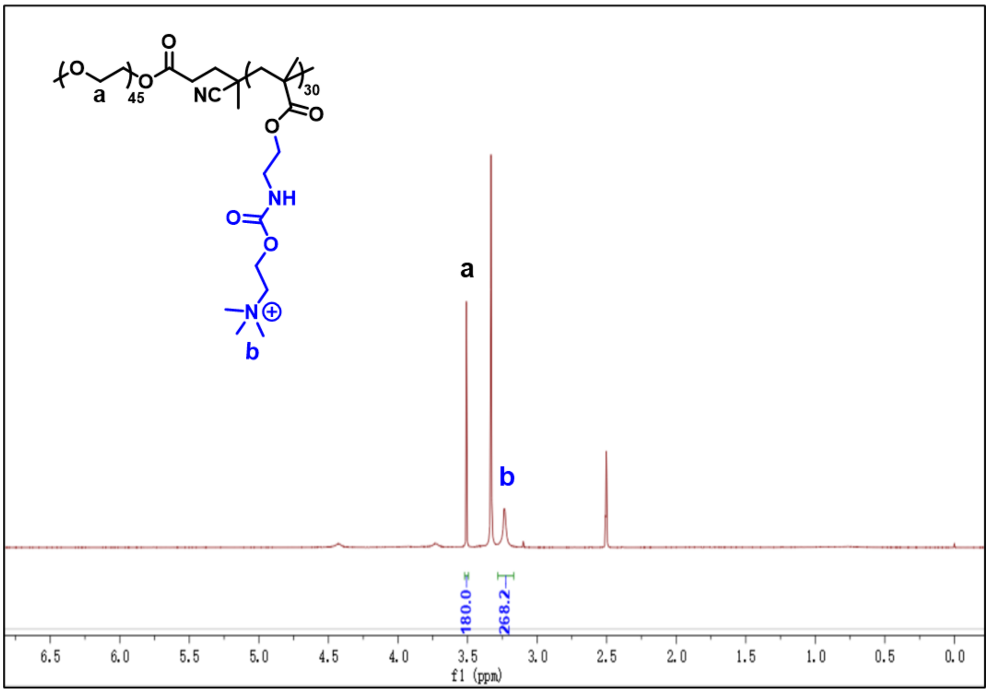

Figure S2. ^{1}H-NMR spectrum recorded for PEG_{45}-*b*-$PTAMA_{30}$ in DMSO-d_6.

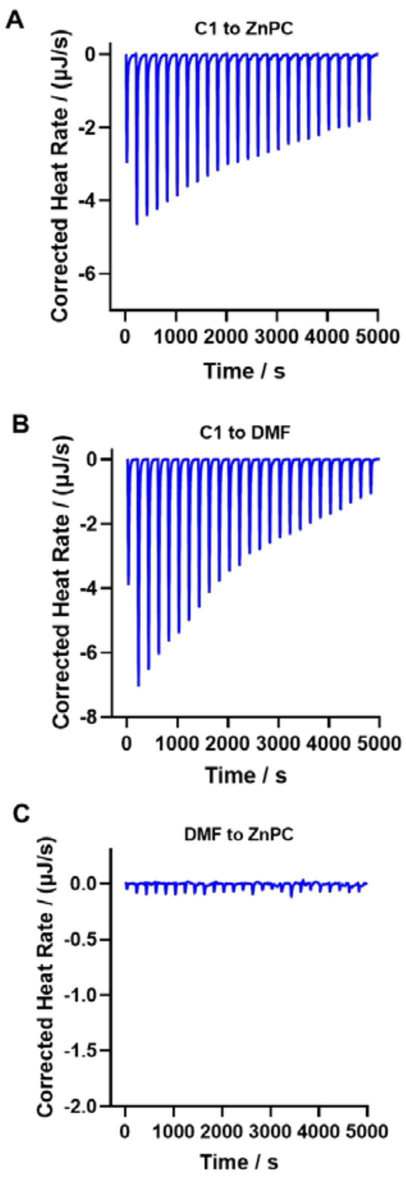

Figure S3. Cation-π interactions between ZnPc and choline-containing model compound (C1) characterized by ITC. (A) Representative ITC data for the titration of C1 to ZnPc. The corresponding heats of dilution of (B) C1 and (C) ZnPc, respectively.

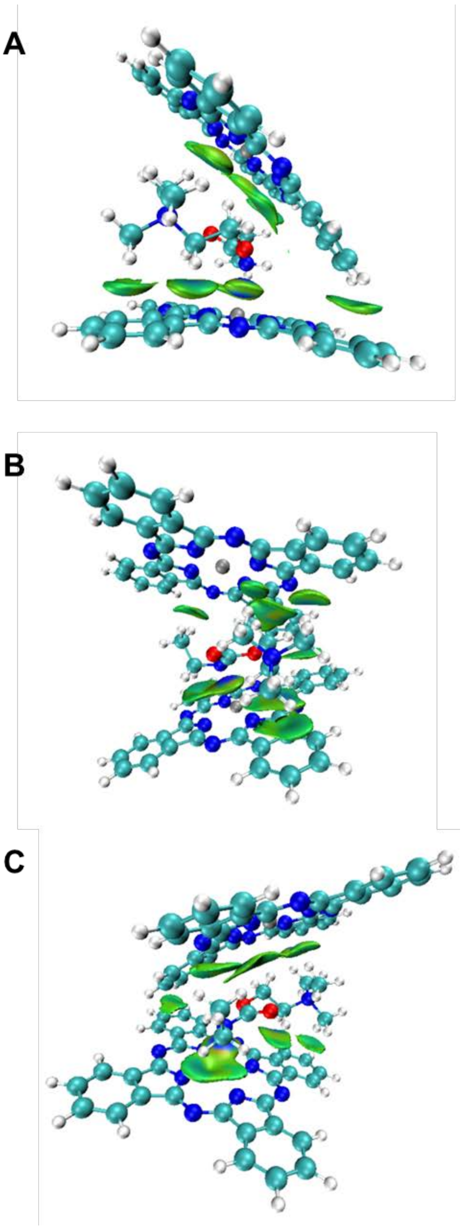

Figure S4. **(**A-C) Different views of independent gradient model (IGM) for the ZnPc/C1/ZnPc complexes with the lowest energy adsorption structures. C1, choline-containing model compound.

A

Trials	P1/mg	ZnPc/μg	Method	Size/nm	PDI
a	2.5	300	1	84.1 ± 2.1	0.208 ± 0.040
b	5	300	1	61.5 ± 1.7	0.125 ± 0.017
c	10	300	1	52.2 ± 3.0	0.148 ± 0.010
d	15	300	1	50.2 ± 2.6	0.190 ± 0.027
e	10	100	1	70.1 ± 1.5	0.178 ± 0.009
f	10	200	1	61.4 ± 3.2	0.192 ± 0.022
g	10	300	2	244.5 ± 23.9	0.167 ± 0.070
h	10	300	3	296.7 ± 26.6	0.189 ± 0.046

B

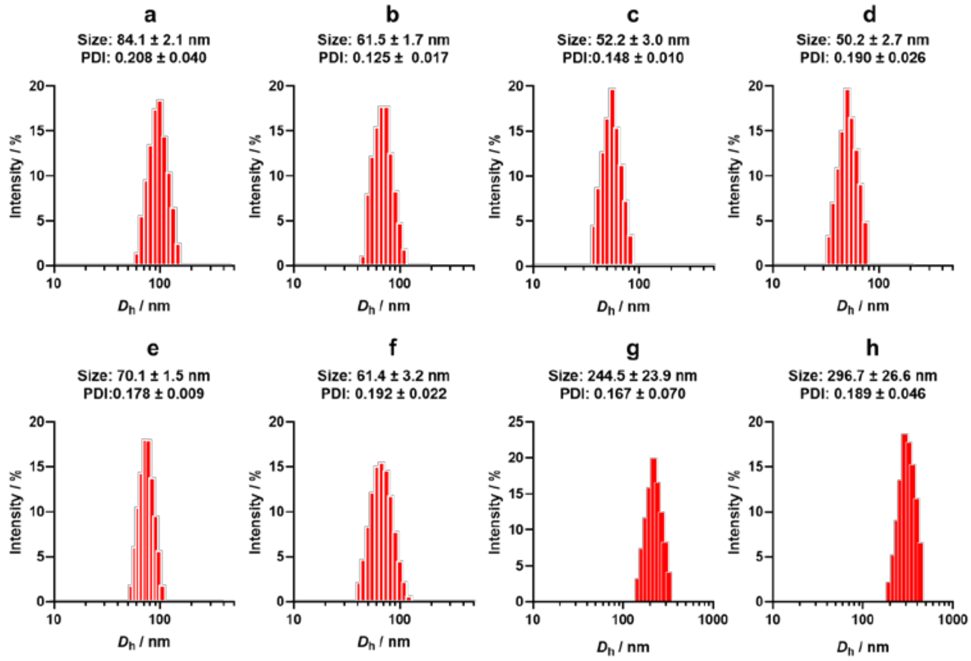

Figure S5. (A) Different conditions were used to prepare NP1/ZnPc by changing the mass ratio of P1 and ZnPc using different preparation methods. Results of these eight trials are shown above. In the method section, "1" represent nanoprecipitation method. "2" represent method commonly used in lipid nanoparticles (micelles and vesicles) preparation. Briefly, P1and ZnPc were dissolved in 1 mL DMF. Then deionized water (2 mL) was added into the organic solution by a syringe pump at a speed of 3 mL/h under stirring (1500 rpm), followed by dropwise addition of 6 mL deionized water for another 3 h. the above dispersion was then transferred into dialysis bag and dialyzed against deionized water. "3" represent method as followed: ZnPc (300 μg) was dissolved in 1 mL DMF and then added into deionized water (9 mL) containing P1 (10 mg) in one-shot under stirring (1500 rpm). After stirring for 10 min at room temperature, the above dispersion was then transferred into dialysis bag and dialyzed against deionized water. (B) Size distribution of NP1/ZnPc prepared by the above methods.

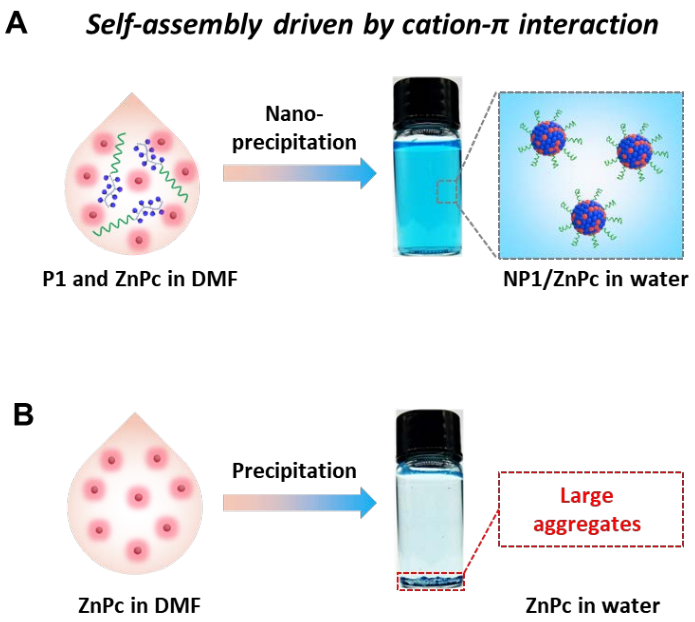

Figure S6. (A) Schematic illustration of cation-π interaction-driven self-assembly of NP1/ZnPc by nanoprecipitation method. (B) Only large aggregates were obtained after nanoprecipitation in water absence of P1 polymers.

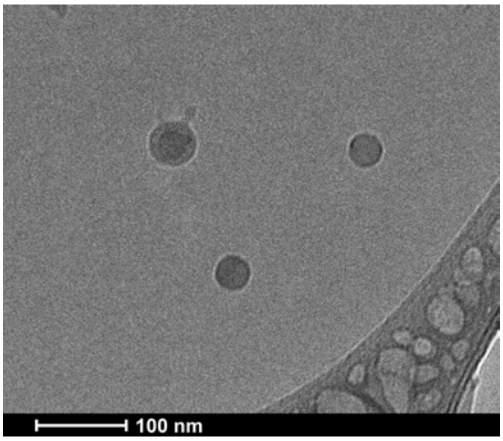

Figure S7. Cryo-TEM image of NP1/ZnPc.

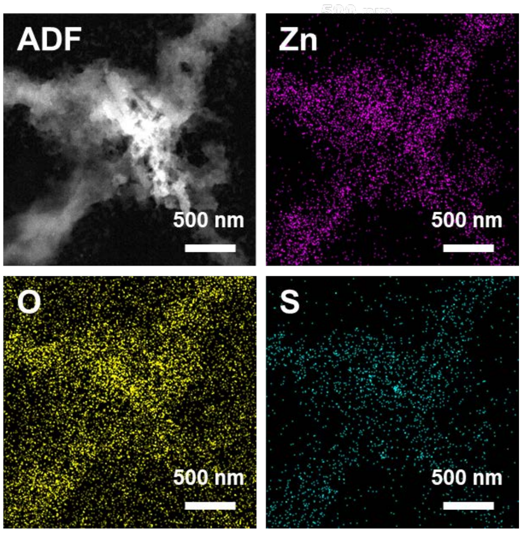

Figure S8. Elemental mapping images of Zn, O, and S in NP1/ZnPc, respectively.

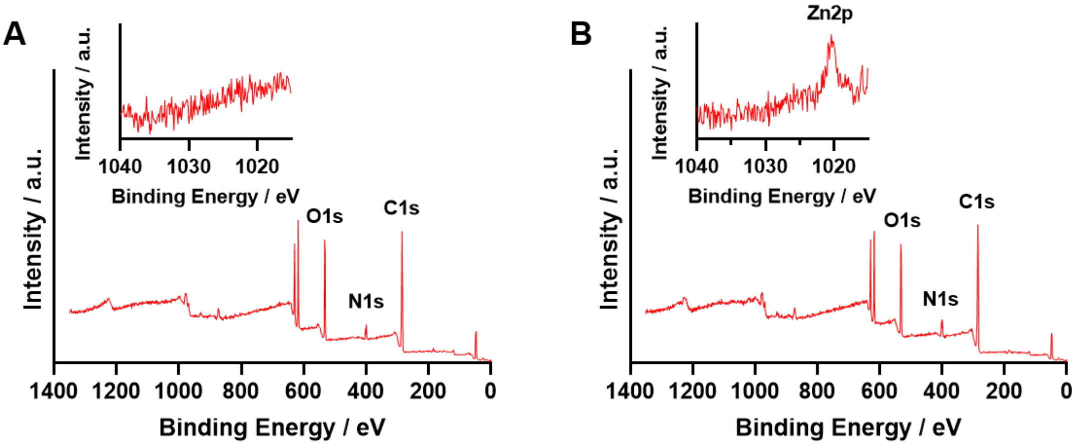

Figure S9. X-ray photoelectron spectroscopy (XPS) spectra of (A) P1, and (B) NP1/ZnPc, respectively.

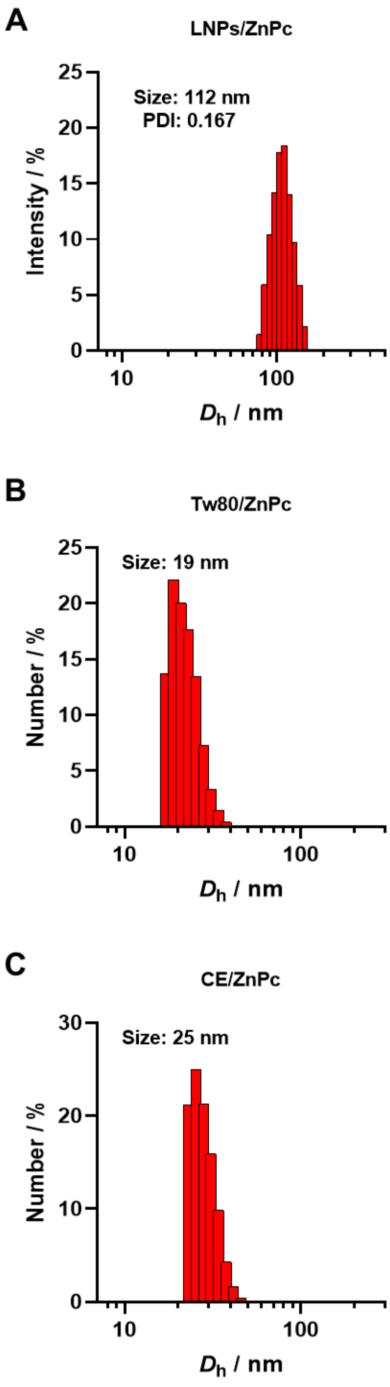

Figure S10. Hydrodynamic diameters, D_h, distributions recorded for (A) LNPs/ZnPc, (B) Tw80/ZnPc, and (C) CE/ZnPc, self-assembled from lipids, Tween 80 (Tw80), and Cremophor EL (CE) with ZnPc respectively.

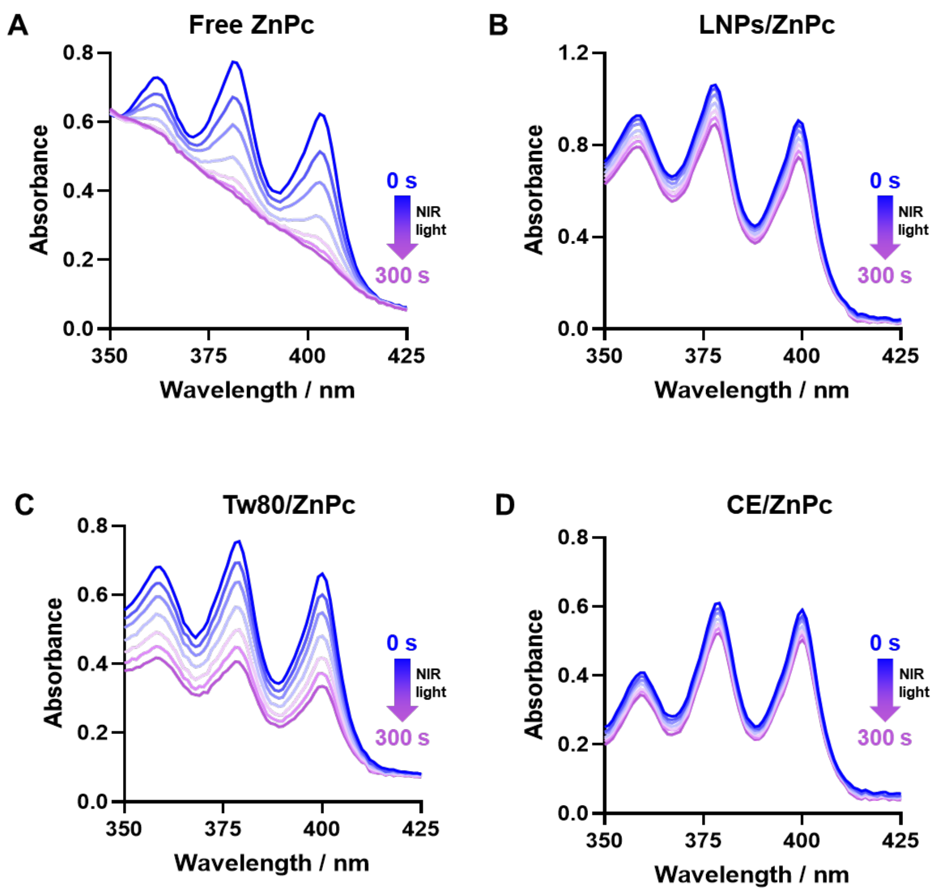

Figure S11. The changes of UV-vis spectra of ABDA (9,10-anthracenediyl-bis (methylene) dimalonic acid, a ROS probe, 50 μM) in the presence of (A) free ZnPc, (B) LNPs/ZnPc, (C) Tw80/ZnPc, and (D) CE/ZnPc were monitored after irradiation for different times in water using LED light (20 mW/cm^2). Free ZnPc was dissolved in DMF, while all the nanoparticles, LNPs/ZnPc, Tw80/ZnPc, and CE/ZnPc, were dispersed in water. All the ZnPc equivalent concentration was 20 μM.

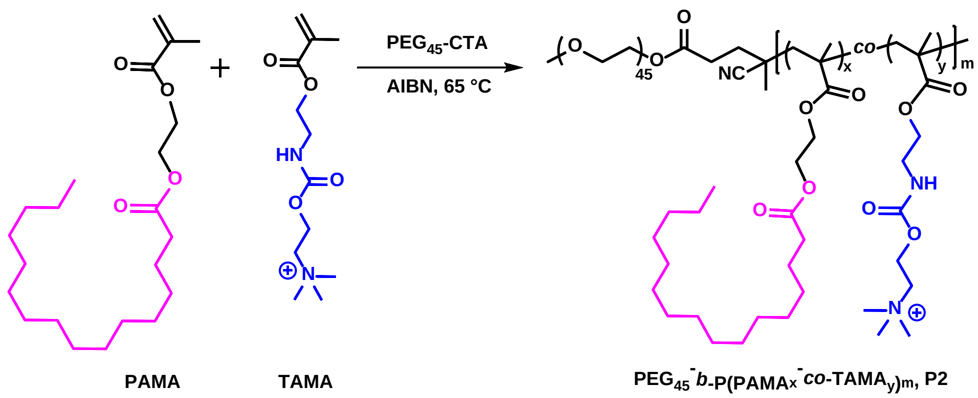

Figure S12. Synthetic route employed for the preparation of amphiphilic diblock copolymer containing hydrophobic palmitic acid moieties and cationic choline moieties, PEG_{45}-*b*-P($PAMA_x$-*co*-$TAMA_y$)$_m$ (P2).

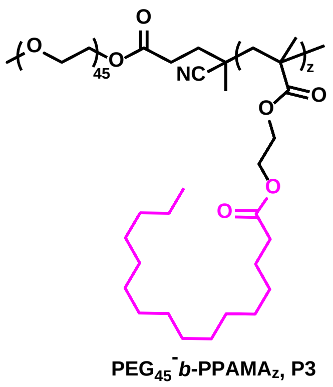

Figure S13. Synthetic route employed for the preparation of amphiphilic diblock polymer containing hydrophobic palmitic acid moieties, PEG_{45}-*b*-$PPAMA_z$ (P3).

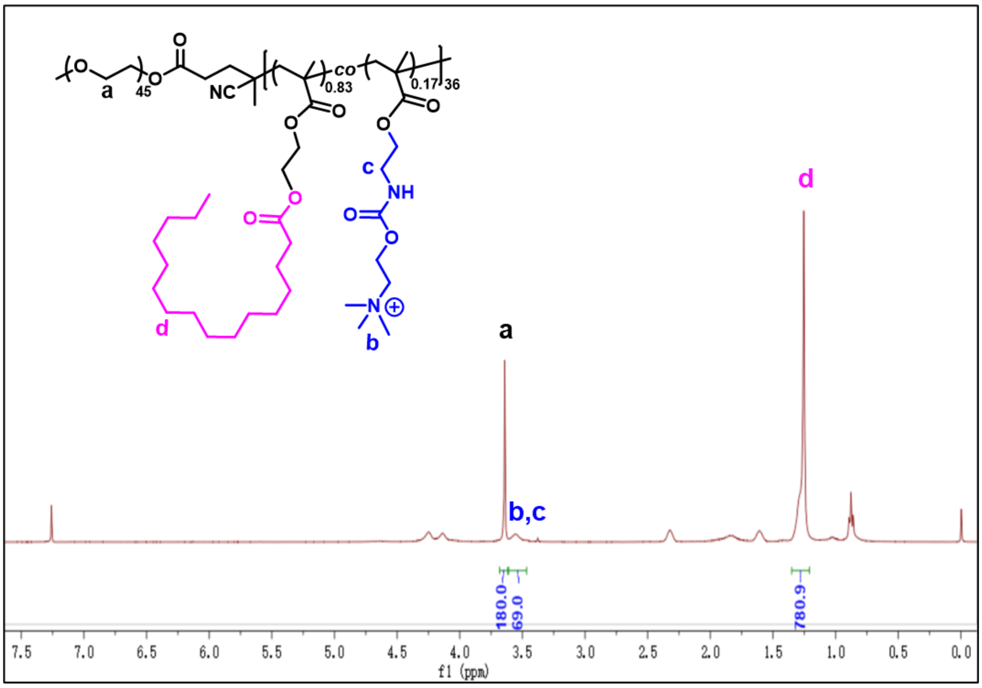

Figure S14. ^{1}H-NMR spectrum recorded for PEG_{45}-*b*-P($PAMA_{0.83}$-*co*-$TAMA_{0.17}$)$_{36}$ in $CDCl_3$.

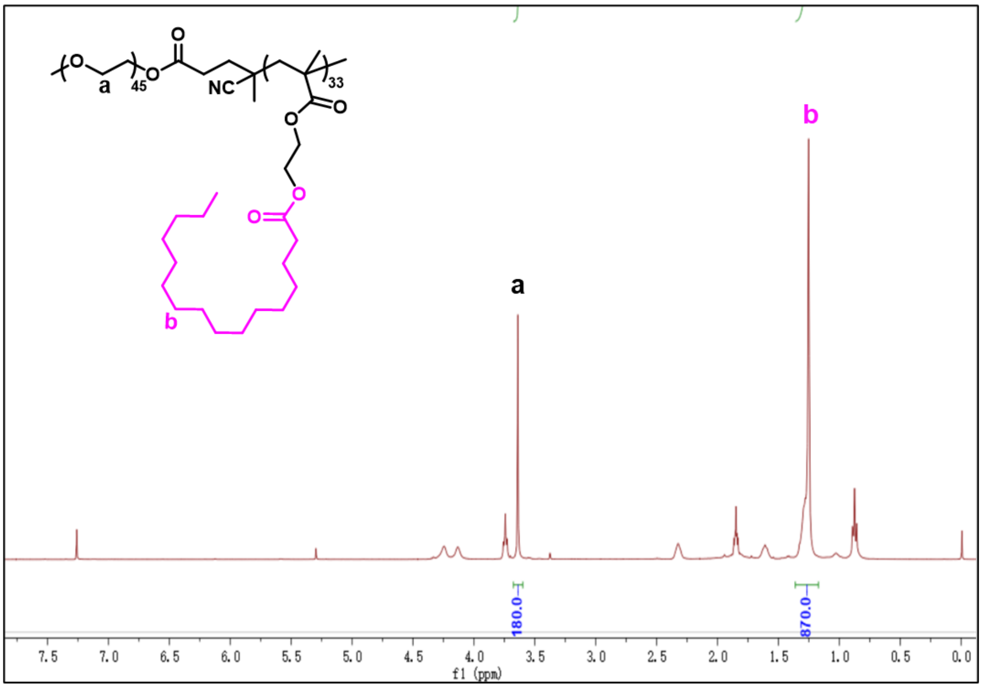

Figure S15. ^{1}H-NMR spectrum recorded for PEG$_{45}$-*b*-PPAMA$_{33}$ in $CDCl_3$.

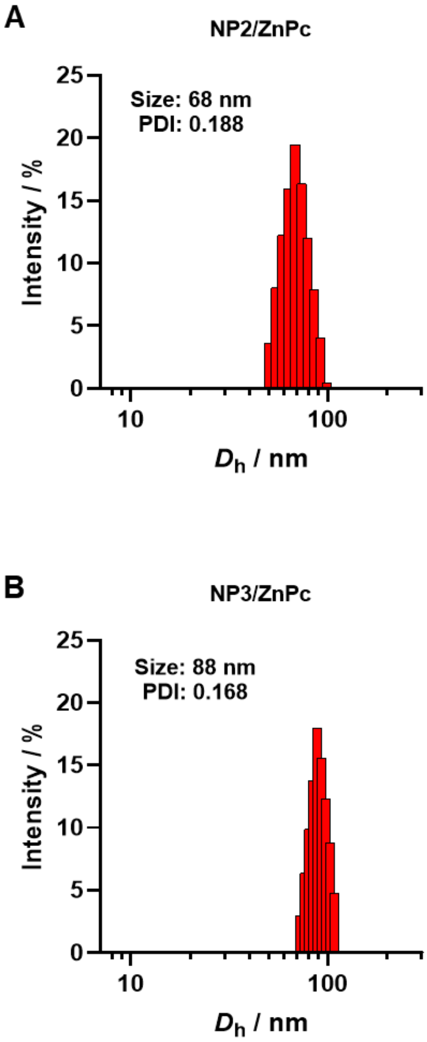

Figure S16. Hydrodynamic diameters, D_h, distributions recorded for (A) NP2/ZnPc, and (B) NP3/ZnPc, self-assembled from P2 and P3 with photosensitizer ZnPc, respectively.

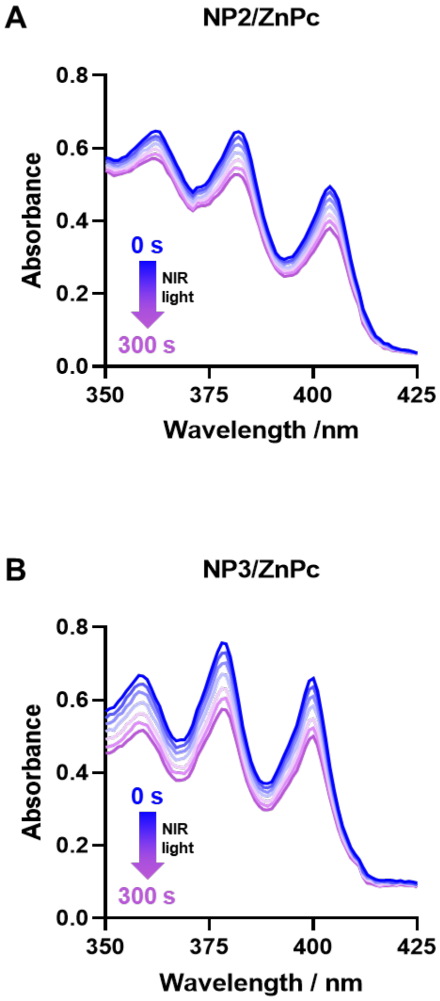

Figure S17. The changes of UV-vis spectra of ABDA (50 μM) in the presence of (A) NP2/ZnPc and (B) NP3/ZnPc were monitored after irradiation for different times in water using LED light (20 mW/cm^2). All the ZnPc equivalent concentration was 20 μM.

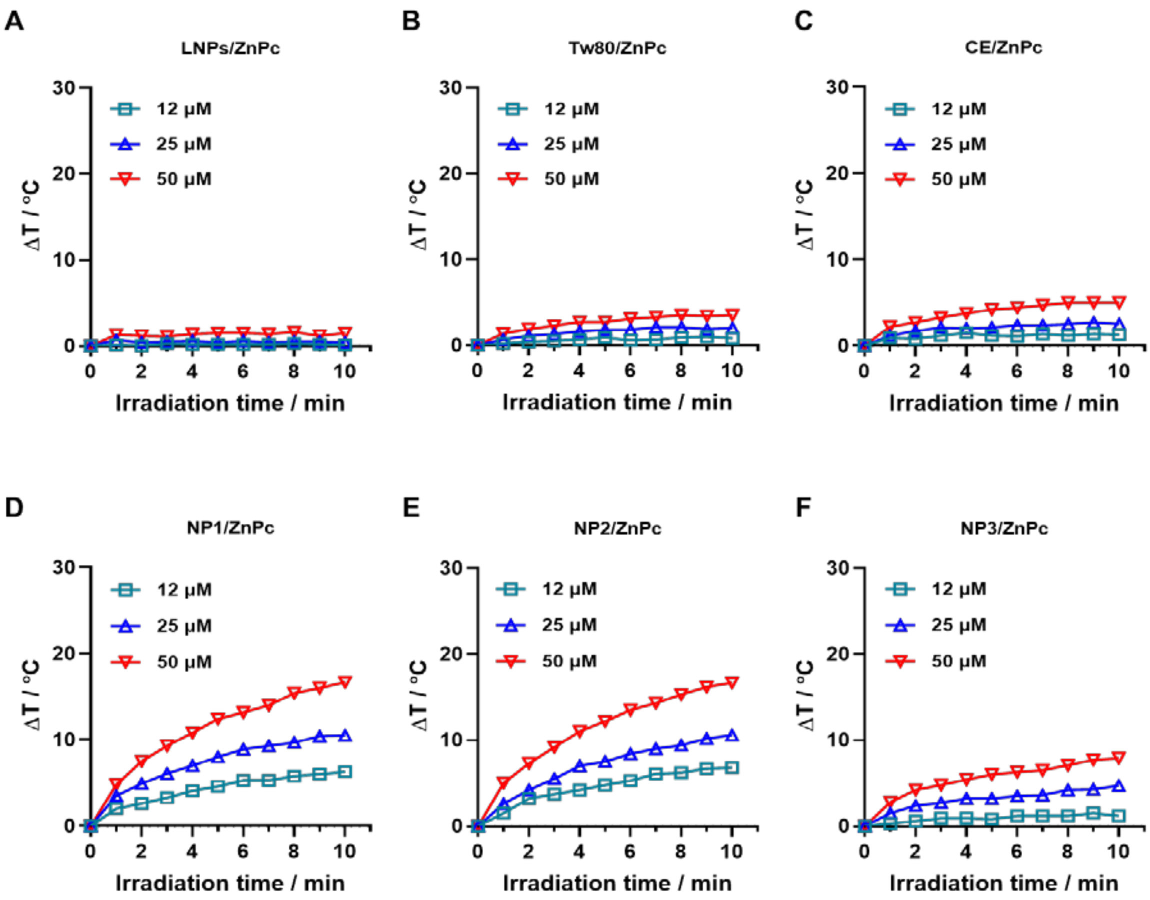

Figure S18. Photothermal effects of (A) LNPs/ZnPc, (B) Tw80/ZnPc, (C) CE/ZnPc, (D) NP1/ZnPc, (E) NP2/ZnPc, and (F) NP3/ZnPc at different ZnPc equivalent concentrations under 660 nm laser irradiation (1.0 W/cm^2), respectively.

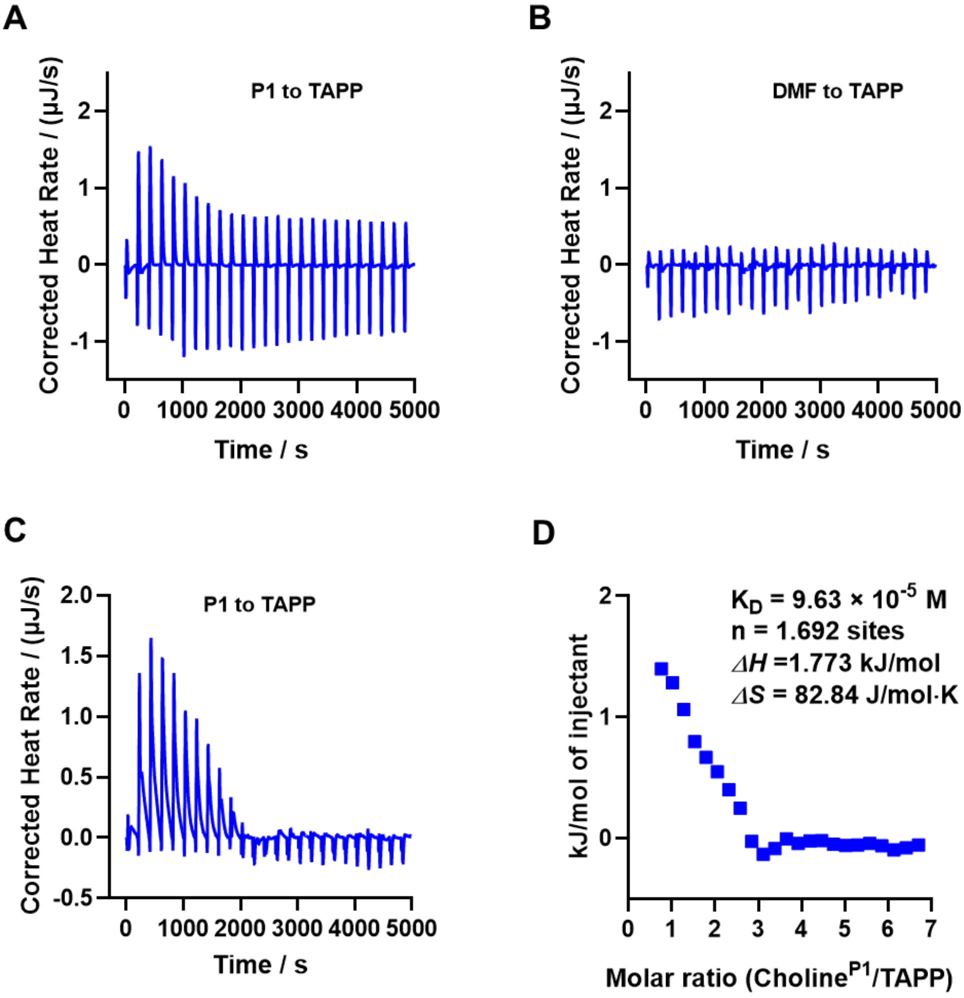

Figure S19. Cation-π interactions between TAPP and P1 characterized by ITC. (A) Representative ITC data for the titration of P1 to TAPP. (B) The corresponding heats of dilution of TAPP. (C) Representative thermogram and (D) binding isotherm analysis.

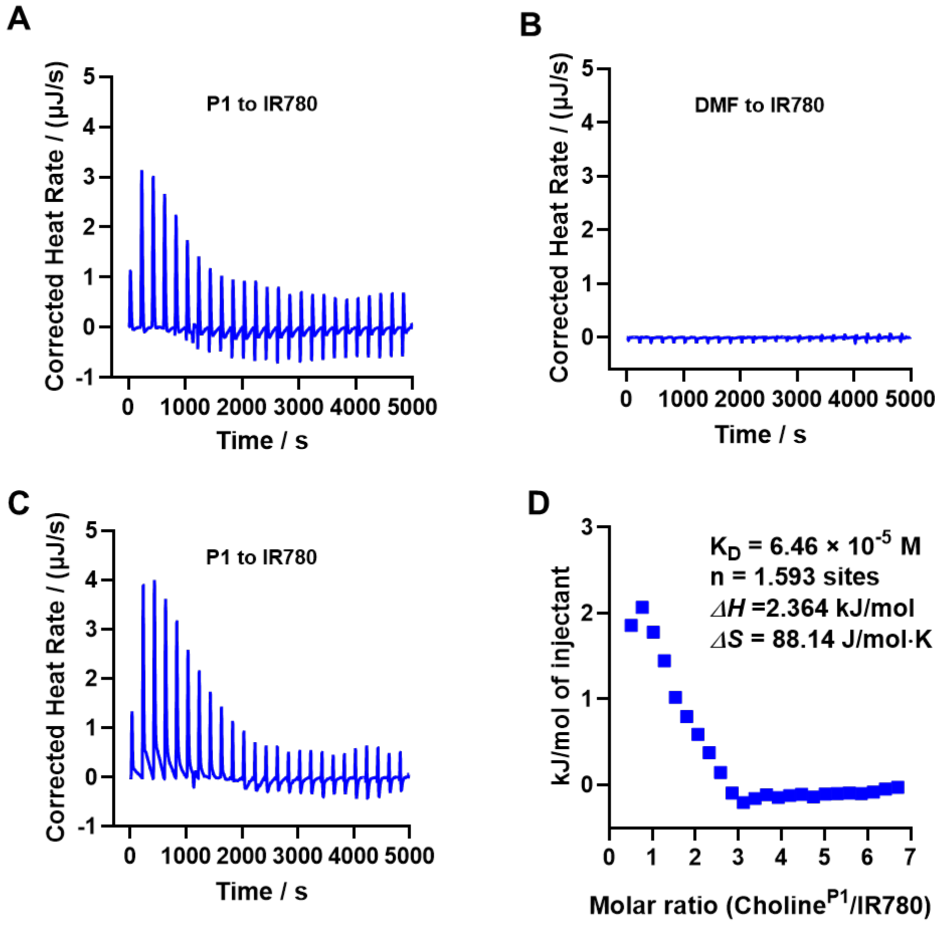

Figure S20. Cation-π interactions between IR780 and P1 characterized by ITC. (A) Representative ITC data for the titration of P1 to IR780. (B) The corresponding heats of dilution of IR780. (C) Representative thermogram and (D) binding isotherm analysis.

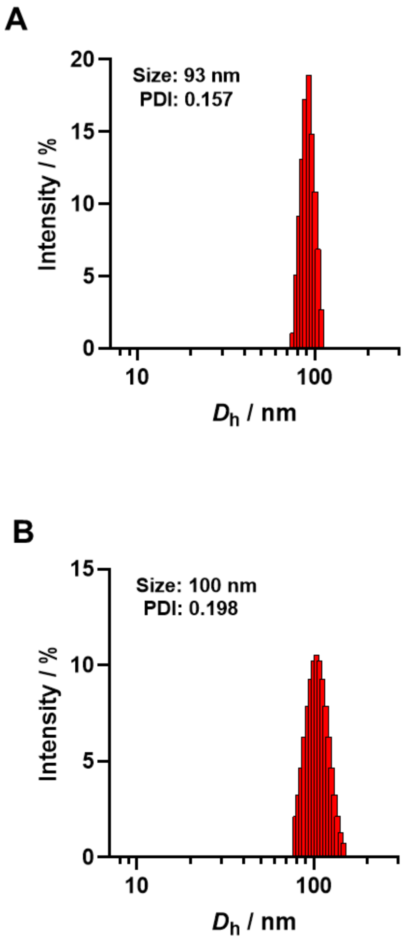

Figure S21. Hydrodynamic diameters, D_h, distributions recorded for (A) NP1/TAPP, and (B) NP1/IR780, self-assembled from P1 with photosensitizers TAPP and IR780, respectively.

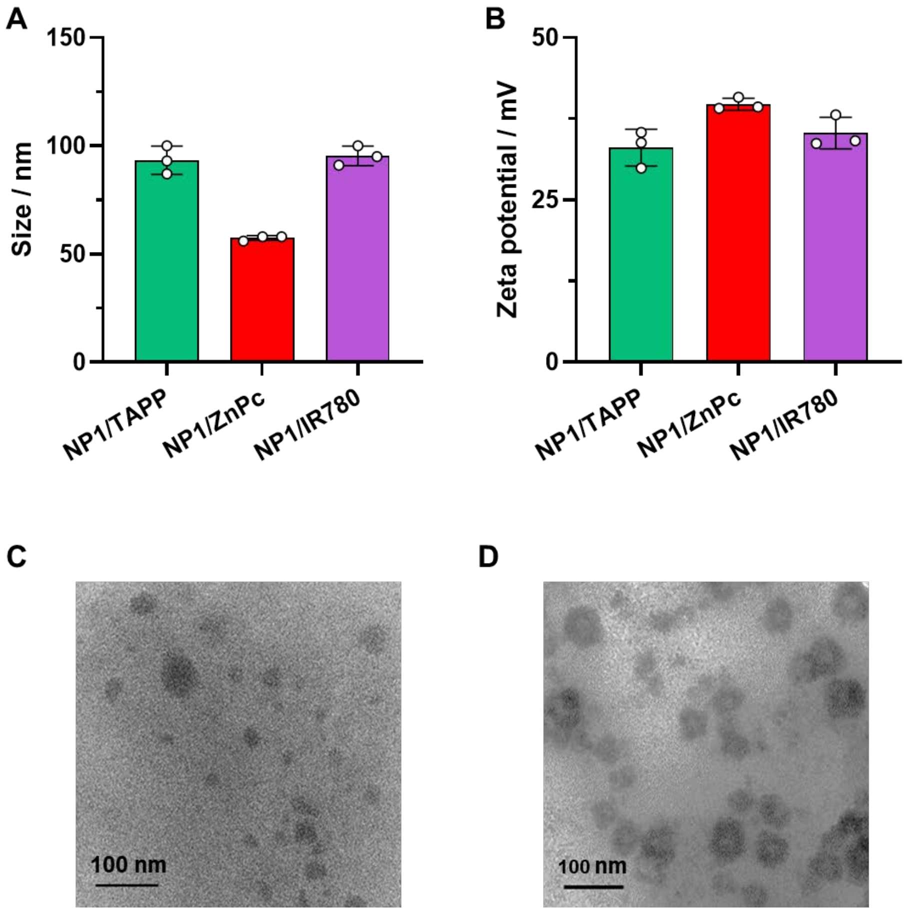

Figure S22. (A) Size and (B) zeta potential recorded for NP1/TAPP, NP1/ZnPc, and NP1/IR780, respectively. (C, D) TEM images of (C) NP1/TAPP and (D) NP1/IR780.

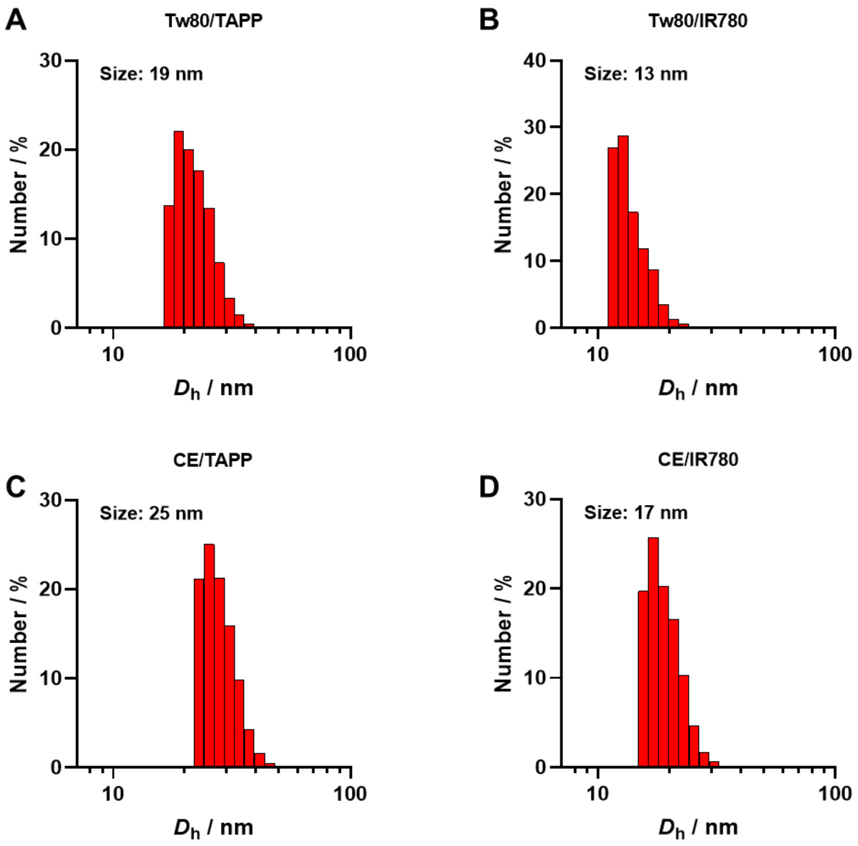

Figure S23. Hydrodynamic diameters, D_h, distributions recorded for (A) Tw80/TAPP, (B) Tw80/IR780, (C) CE/TAPP, and (D) CE/IR780, self-assembled from Tween 80 (Tw80) with photosensitizers (A) TAPP and (B) IR780, and Cremophor EL (CE) with (C) TAPP and (D) IR780, respectively.

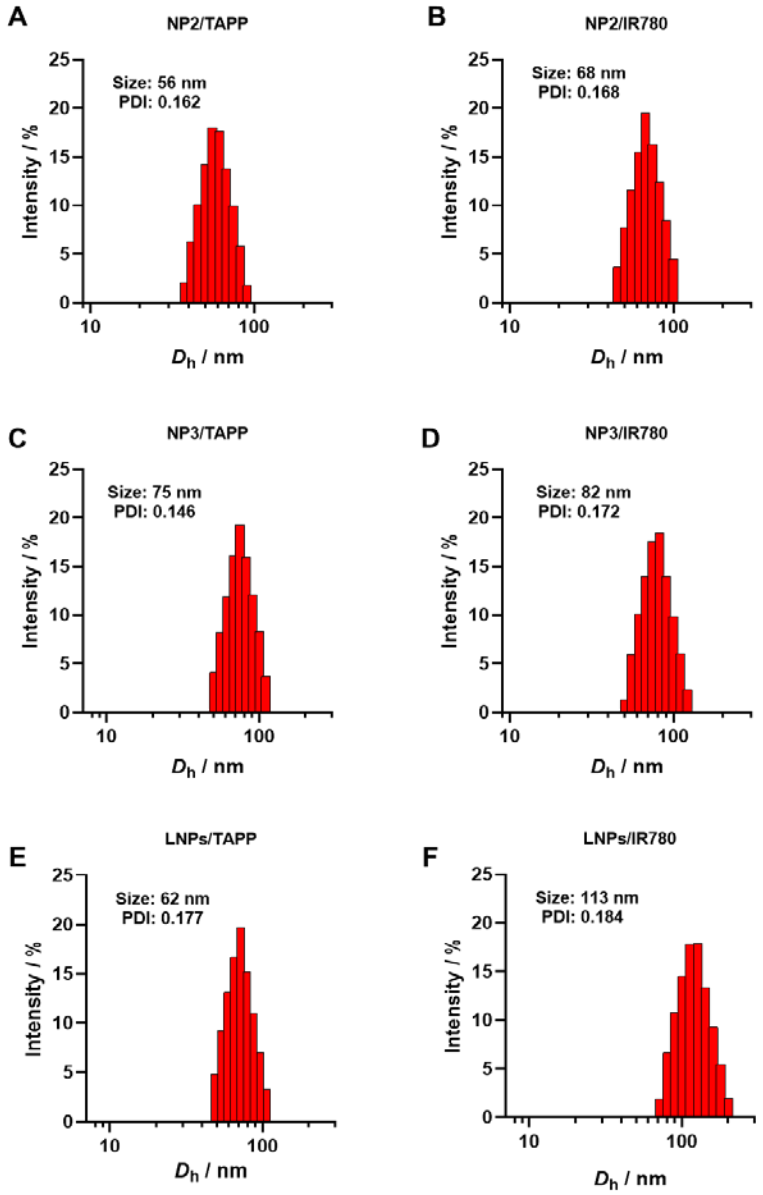

Figure S24. Hydrodynamic diameters, D_h, distributions recorded for (A) NP2/TAPP, (B) NP2/IR780, (C) NP3/TAPP, (D) NP3/IR780, (E) LNPs/TAPP, and (F) LNPs/IR780, self-assembled from P2 with photosensitizers (A) TAPP and (B) IR780, P3 with (C) TAPP and (D) IR780, and lipids with (E) TAPP and (F) IR780, respectively.

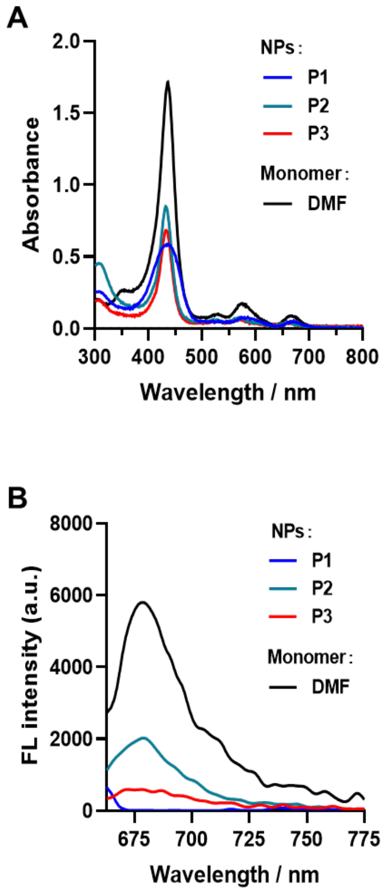

Figure S25. (A) Absorbance spectra and (B) fluorescence spectra recorded for free TAPP (monomer) dissolved in DMF, NP1/TAPP, NP2/TAPP, and NP3/TAPP formulated by P1, P2, and P3 in water, respectively. All the ZnPc equivalent concentration was 20 μM.

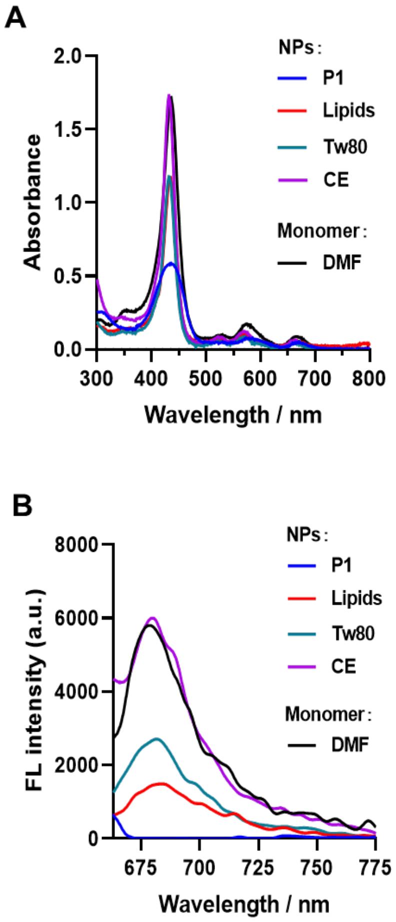

Figure S26. (A) Absorbance spectra and (B) fluorescence spectra recorded for free TAPP (monomer) dissolved in DMF, NP1/TAPP, LNPs/TAPP, Tw80/TAPP, and CE/TAPP formulated by P1, lipids, Tween-80, and Cremophor EL in water, respectively. All the TAPP equivalent concentration was 20 μM.

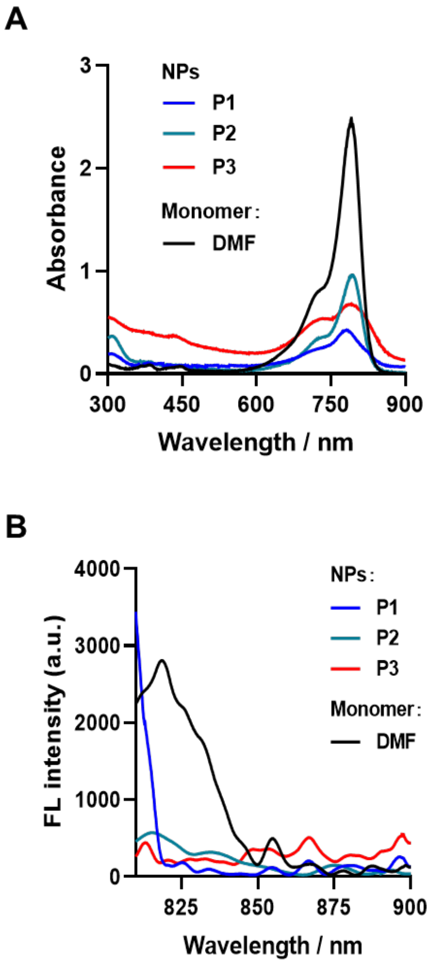

Figure S27. (A) Absorbance spectra and (B) fluorescence spectra recorded for free IR780 (monomer) dissolved in DMF, NP1/IR780, NP2/IR780, and NP3/IR780 formulated by P1, P2, and P3 in water, respectively. All the IR780 equivalent concentration was 20 μM.

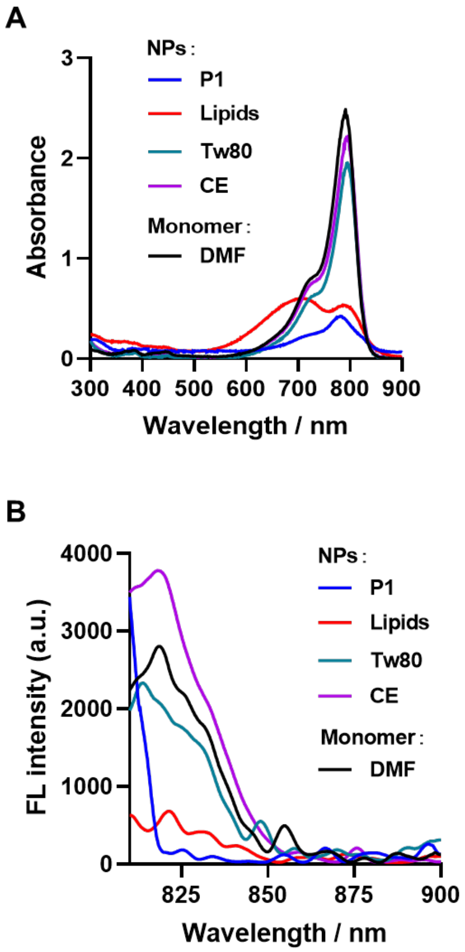

Figure S28. (A) Absorbance spectra and (B) fluorescence spectra recorded for free IR780 (monomer) dissolved in DMF, NP1/IR780, LNPs/IR780, Tw80/IR780, and CE/IR780 formulated by P1, lipids, Tween-80, and Cremophor EL in water, respectively. All the IR780 equivalent concentration was 20 μM.

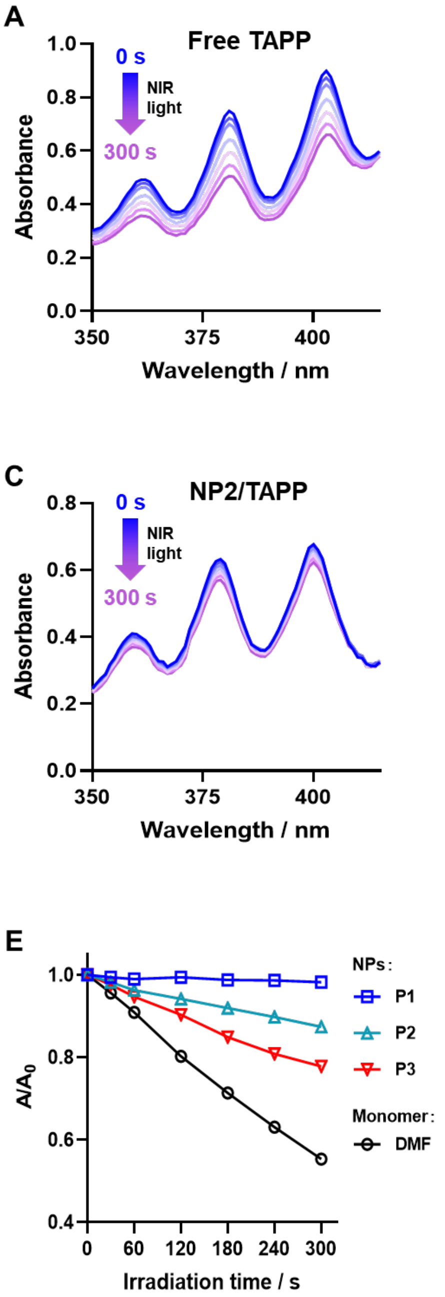

Figure S29. The changes of UV-vis spectra of ABDA (50 µM) in the presence of (A) Free TAPP, (B) NP1/TAPP, (C) NP2/TAPP, and (D) NP3/TAPP were monitored after irradiation for different times in water using LED light (20 mW/cm^2). (E) Plot of A/A_0 vs light irradiation time. For each sample, A_0 and A are the ABDA absorbance at 378 nm before and after irradiation, respectively. All the TAPP equivalent concentration was 20 µM.

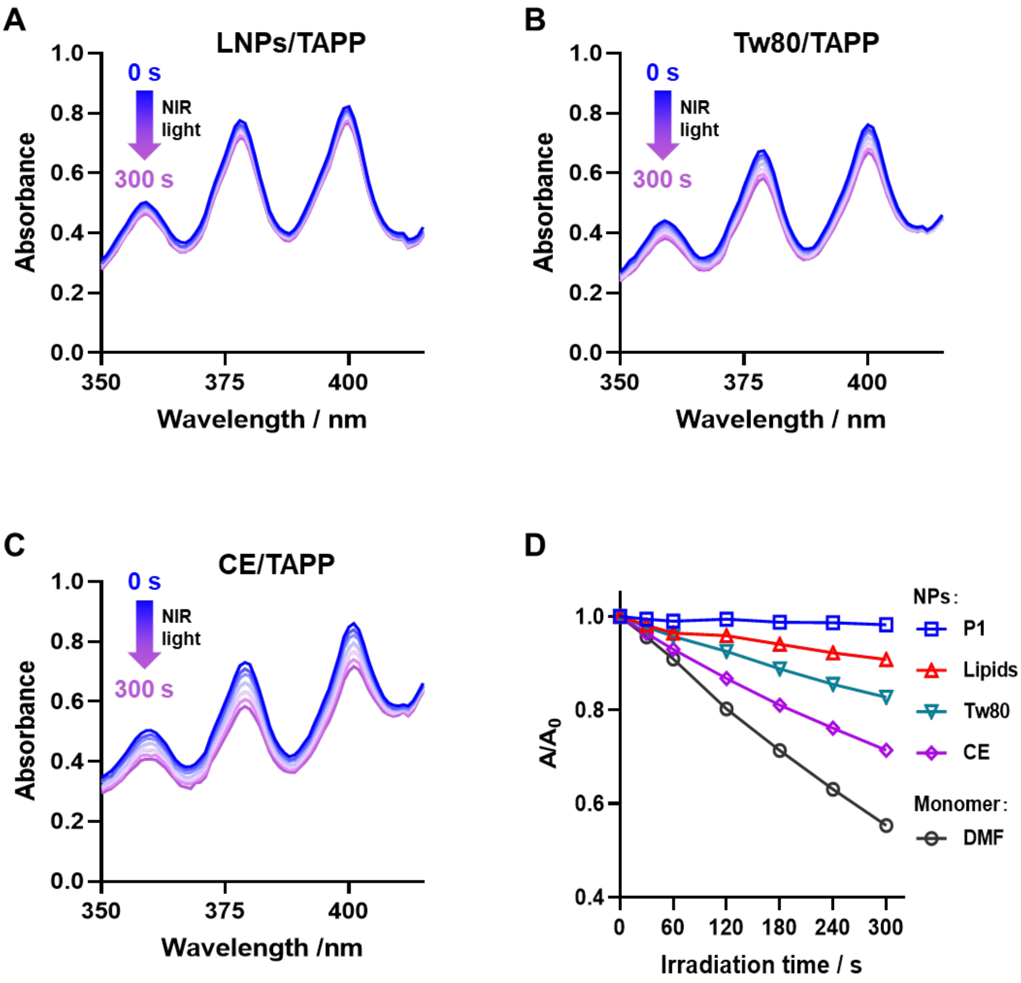

Figure S30. The changes of UV-vis spectra of ABDA (50 μM) in the presence of (A) LNPs/TAPP, (B) Tw80/TAPP, and (C) CE/TAPP were monitored after irradiation for different times in water using LED light (20 mW/cm^2). (D) Plot of A/A_0 vs light irradiation time. All the TAPP equivalent concentration was 20 μM.

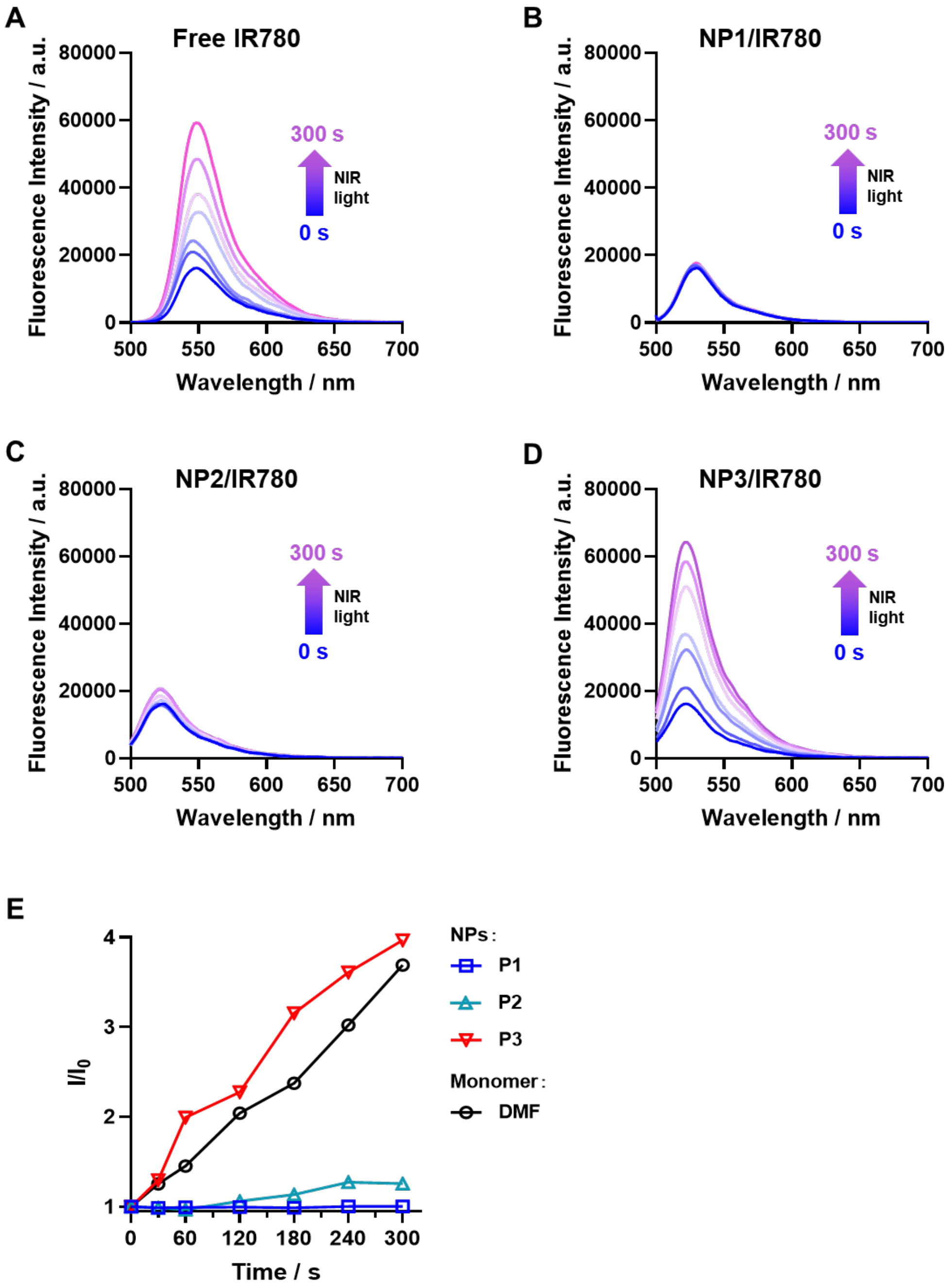

Figure S31. The changes of fluorescence spectra of DCFH (2,7-dichlorodihydrofluorescein, a ROS probe, 50 μM) in the presence of (A) Free IR780, (B) NP1/IR780, (C) NP2/IR780, and (D) NP3/IR780 were monitored after irradiation for different times in water using 808 nm laser (20 mW/cm^2). (E) Plot of I/I_0 vs light irradiation time. For each sample, I_0 and I are the DCFH fluorescence intensity at 522 nm before and after irradiation, respectively. All the IR780 equivalent concentration was 20 μM. λ_{ex} = 488 nm.

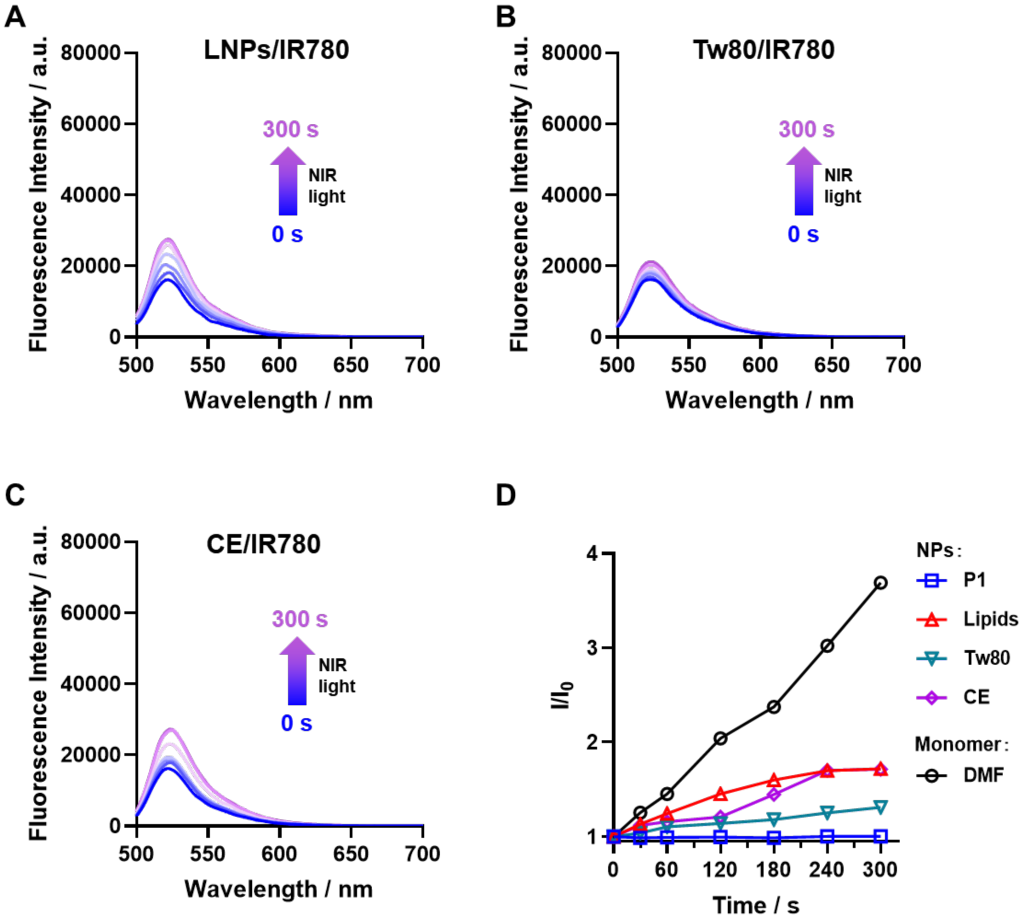

Figure S32. The changes of fluorescence spectra of DCFH (50 μM) in the presence of of (A) LNPs/IR780, (B) Tw80/IR780, and (C) CE/IR780 were monitored after irradiation for different times in water using 808 nm laser (20 mW/cm^2). (D) Plot of I/I_0 vs light irradiation time. For each sample, I_0 and I are the DCFH fluorescence intensity at 522 nm before and after irradiation, respectively. All the IR780 equivalent concentration was 20 μM. λ_{ex} = 488 nm.

A

$$\text{Relative PDT OFF index}(\%)=(1-\frac{1-A_t^{NPs}/A_0^{NPs}}{1-A_t^{Monomer}/A_0^{Monomer}})\times 100\%$$

B

$$\text{Relative PDT OFF index}(\%)=(1-\frac{I_t^{NPs}/I_0^{NPs}}{I_t^{Monomer}/I_0^{Monomer}})\times 100\%$$

Figure S33. (A) The formula used for calculation of Relative PDT OFF index for ZnPc and TAPP. $A_t^{NPs}$and $A_t^{monomer}$ represented the OD value of ABDA at 380 nm in the presence of photosensitizer NPs or free photosensitizer after irradiation for 5 min, respectively. $A_0^{NPs}$and $A_0^{monomer}$represented the OD value of ABDA at 380 nm in the presence of photosensitizer NPs or free photosensitizer without irradiation, respectively. (B) The formula used for calculation of Relative PDT OFF index for IR780. $I_t^{NPs}$and $I_t^{monomer}$ represented the FL intensity value of DCFH at 522 nm in the presence of photosensitizer NPs or free photosensitizer after irradiation for 5 min, respectively. I_0^{NPs} and $I_0^{monomer}$represented the FL intensity value of DCFH at 522 nm in the presence of photosensitizer NPs or free photosensitizer without irradiation, respectively.

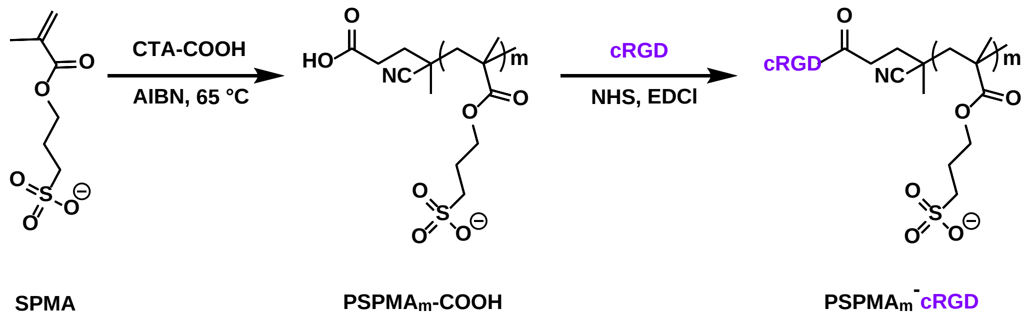

Figure S34. Synthetic routes employed for the preparation of negatively charged and tumor-targeting polymer PSPMA$_m$-cRGD.

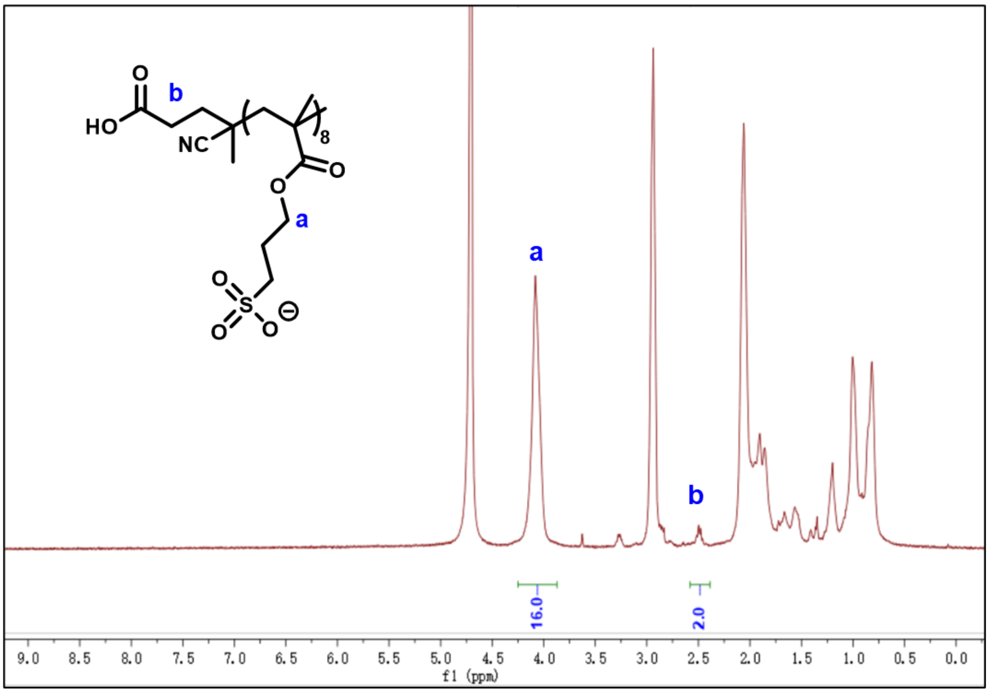

Figure S35. ^{1}H-NMR spectrum recorded for $PSPMA_8$ in D_2O.

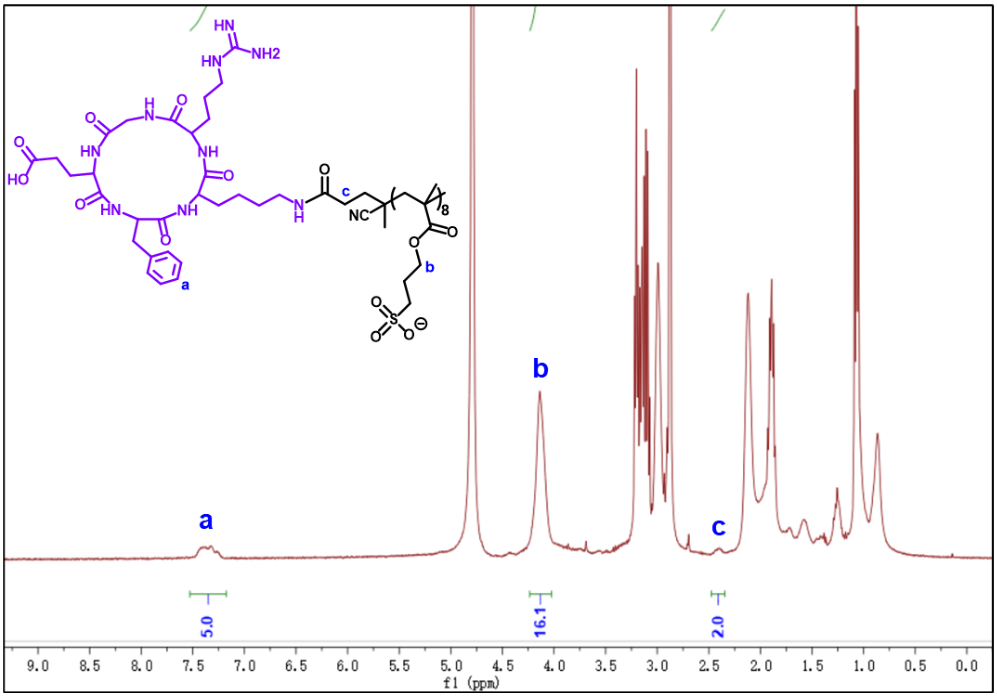

Figure S36. ^{1}H-NMR spectrum recorded for PSPMA$_8$-cRGD in D_2O.

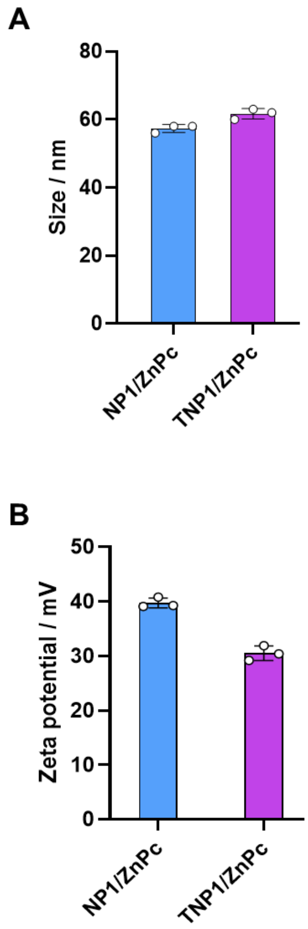

Figure S37. (A) Hydrodynamic diameters and (B) zeta potential recorded for NP1/ZnPc and TNP1/ZnPc, respectively.

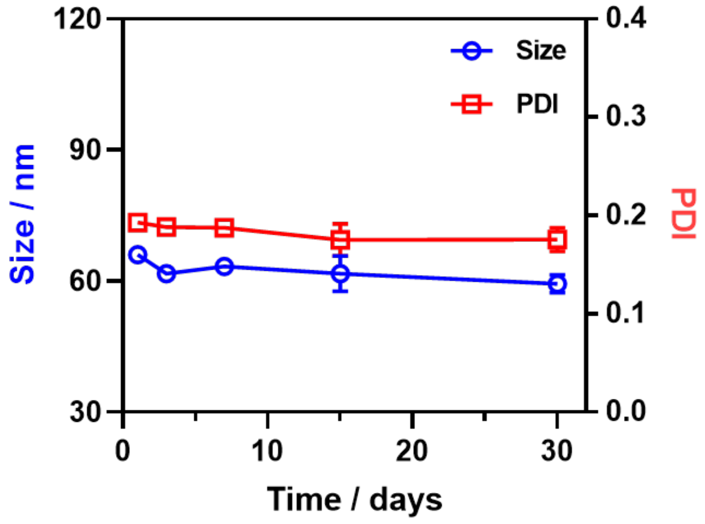

Figure S38. Long-term storage stability of TNP1/ZnPc at 4 °C in PBS.

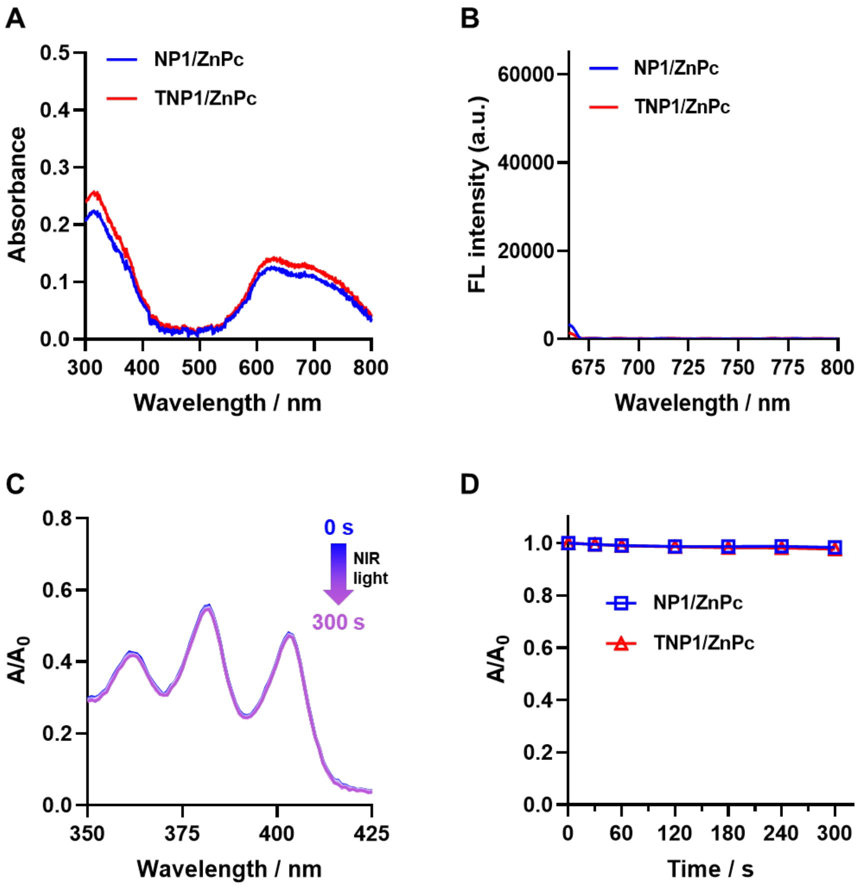

Figure S39. (A) The absorption and (B) fluorescence emission spectra (λ_{ex} = 660 nm) recorded for NP1/ZnPc and TNP1/ZnPc, respectively. (C) The changes of UV-vis spectra of ABDA (50 μM) in the presence of TNP1/ZnPc were monitored after irradiation for different times in water using 660 nm LED light (20 mW/cm^2). (D) Plot of A/A_0 vs light irradiation time for NP1/ZnPc and TNP1/ZnPc.

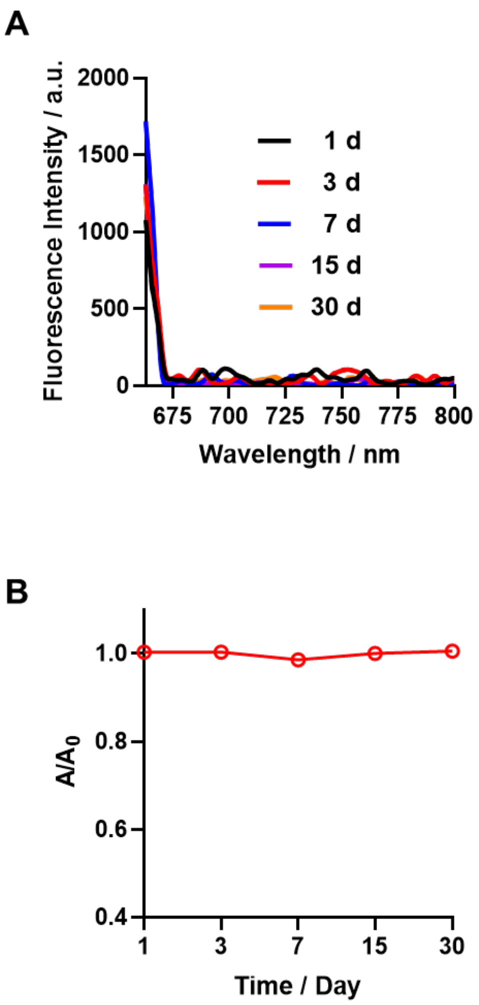

Figure S40. OFF state stability of TNP1/ZnPc during long-term storage at 4 °C in PBS through (A) fluorescence and (B) ROS generation measurements.

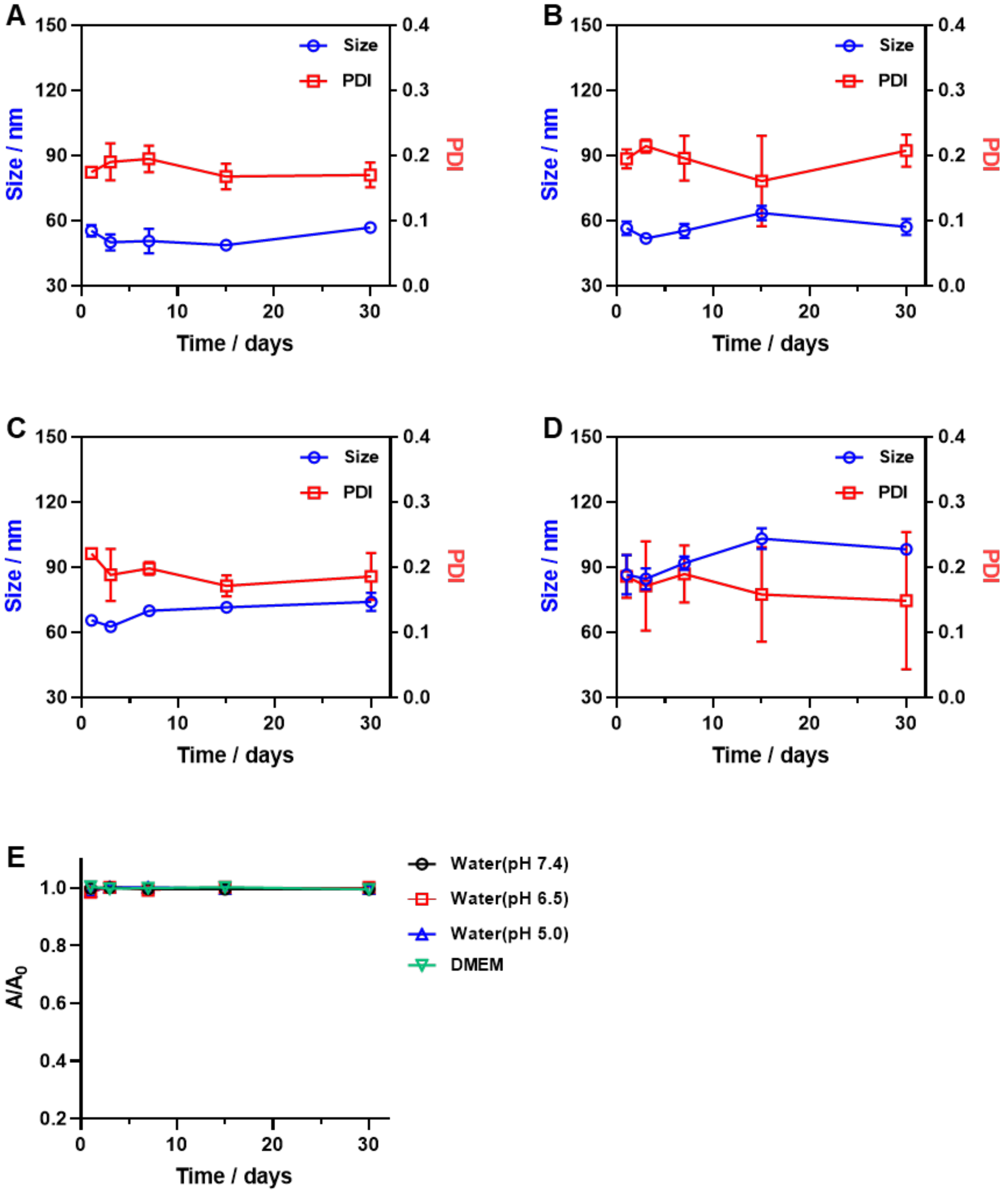

Figure S41. Size and PDI stability study of TNP1/ZnPc after exposure to water under different pH or DMEM culture medium, (A) pH 7.4, (B) pH 6.5, (C) pH 5.0, and (D) DMEM culture medium respectively. (E) The OFF-state stability of the photoactivity of TNP1/ZnPc after exposure to water under different pH or DMEM culture medium, respectively.

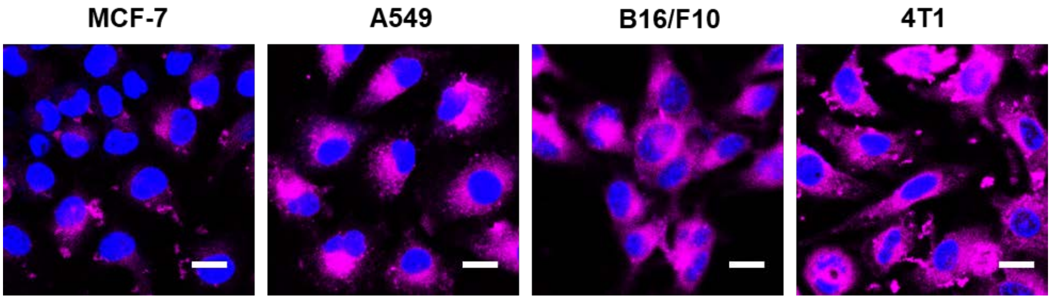

Figure S42. CLSM images of tumor cell lines with different expression levels of integrin receptor after incubation with TNP1/ZnPc for 2 h, respectively. MCF-7 was used as low integrin receptor-expressed cell line, A549, B16F10 and 4T1 were used as high integrin receptor-expressed cell lines. ZnPc concentration: 5 μM. Scale bar, 20 μm.

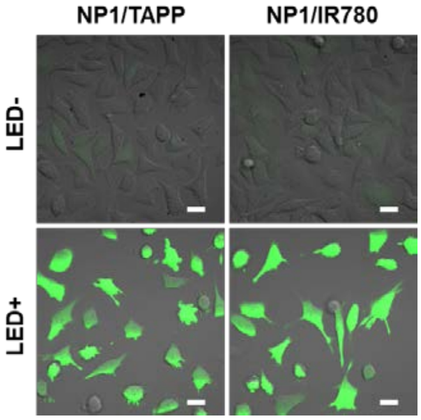

Figure S43. CLSM images of DCFH-DA incubated 4T1 cells after treatments with NP1/TAPP or NP1/IR780 for 2 h in dark or upon light irradiation (530 nm, 20 mW/cm^2, 5 min for NP1/TAPP; 808 nm, 20 mW/cm^2, 5 min for NP1/IR780). Scale bar, 20 μm.

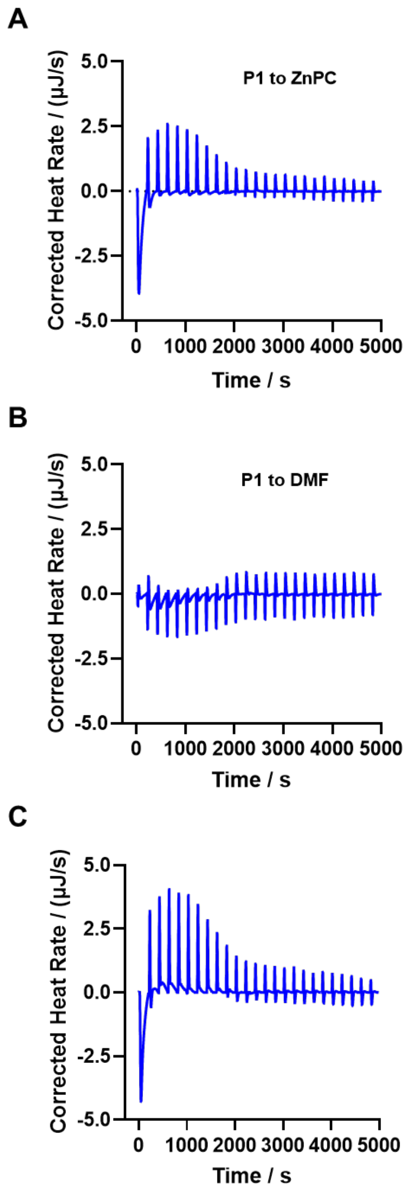

Figure S44. Cation-π interactions between ZnPc and P1 characterized by ITC. (A) Representative ITC data for the titration of P1 to ZnPc, and (B) the corresponding heats of dilution of P1. (C) Representative thermogram analysis.

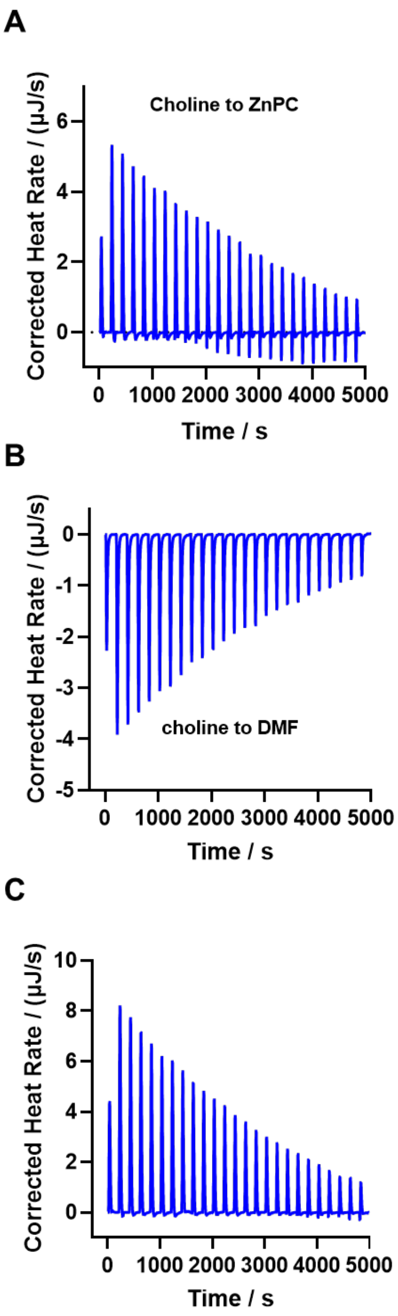

Figure S45. Cation-π interactions between ZnPc and choline characterized by ITC. (A) Representative ITC data for the titration of choline to ZnPc, and (B) the corresponding heats of dilution of choline. (C) Representative thermogram analysis.

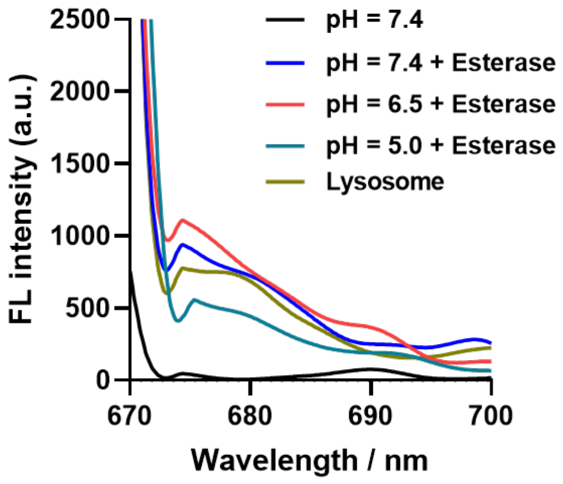

Figure S46. Changes of fluorescence emission intensities of ZnPc in TNP1/ZnPc before and after incubation with esterase at different pH, or extracted lysosome for 2 h at 37 °C. λ_{ex} = 660 nm.

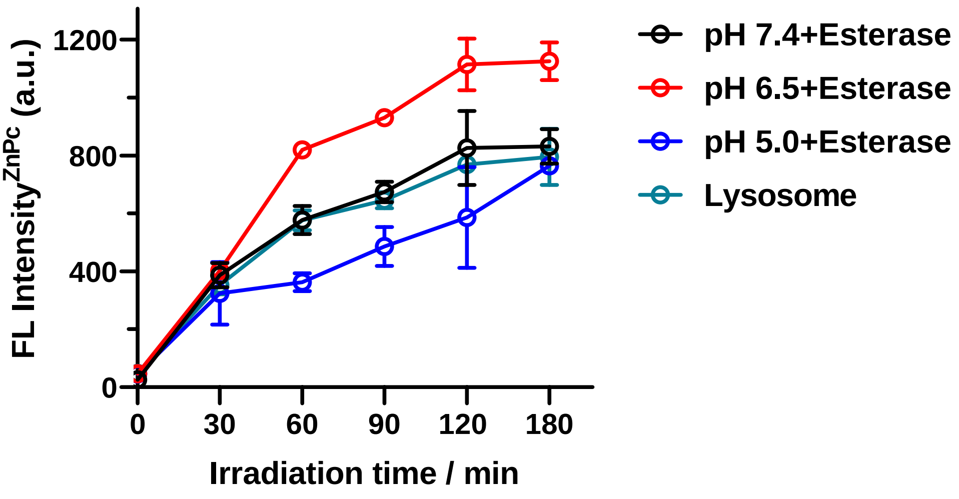

Figure S47. Change of fluorescence emission intensities of ZnPc in TNP1/ZnPc after incubation with esterase at different pH, or extracted lysosome for various incubation time at 37 °C. λ_{ex} = 660 nm.

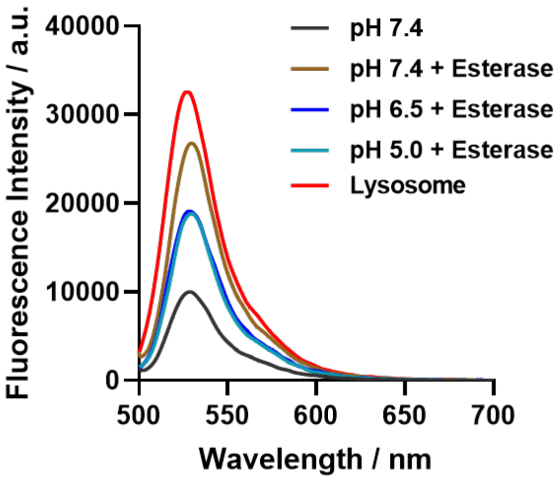

Figure S48. Fluorescence emission spectra of DCFH (ROS probe) recorded for TNP1/ZnPc before and after incubation with esterase at different pH, or extracted lysosome for 2 h at 37 °C. Samples were monitored after irradiation for 5 min using LED light (20 mW/cm^2). λ_{ex} = 488 nm.

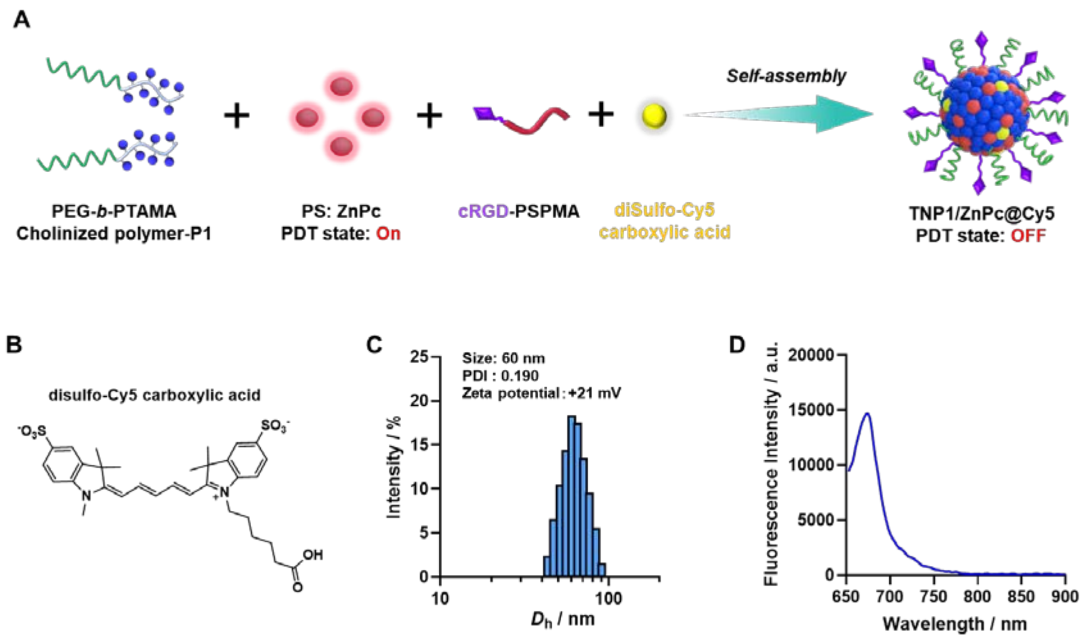

Figure S49. (A) Schematic illustration of the construction of Cy5-labeled TNP1/ZnPc (TNP1/ZnPc@Cy5). DiSulfo-Cy5 carboxylic acid was encapsulated based on electrostatic interactions. (B) chemical structure of diSulfo-Cy5 carboxylic acid. (C) Hydrodynamic diameters, D_h, distributions recorded for TNP1/ZnPc@Cy5. (D) Fluorescence emission spectrum recorded for TNP1/ZnPc@Cy5 (λ_{ex} = 650 nm).

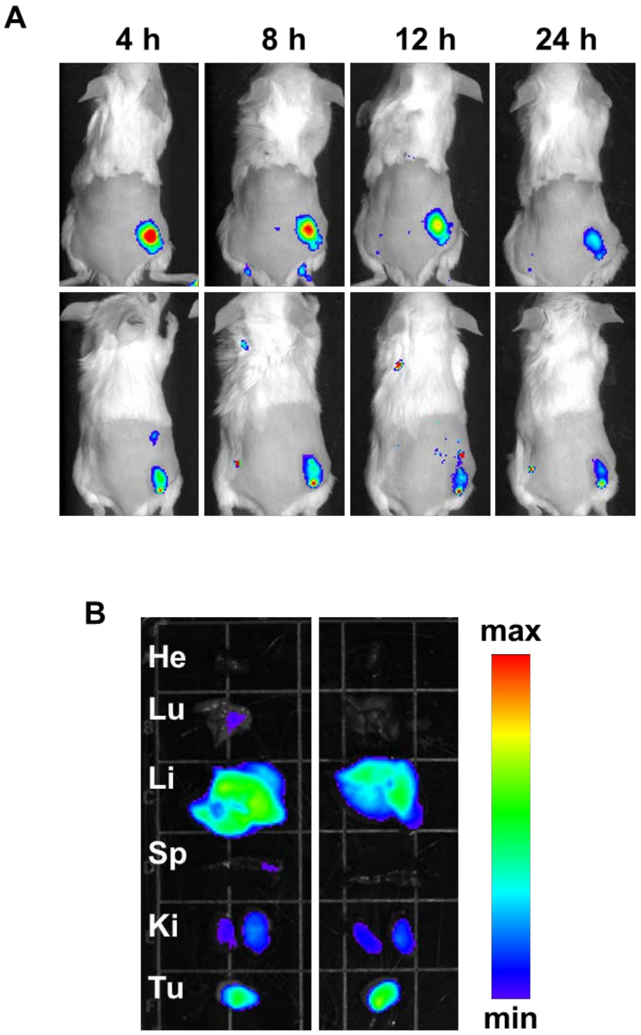

Figure S50. (A) In vivo imaging of 4T1 tumor-bearing BALB/c mice at 4, 8, 16, and 24 h after *intravenous* injection of TNP1/ZnPc@Cy5. (B) Ex vivo imaging of fluorescence signals in tumors and major organs at 24 h post-injection of TNP1/ZnPc@Cy5.

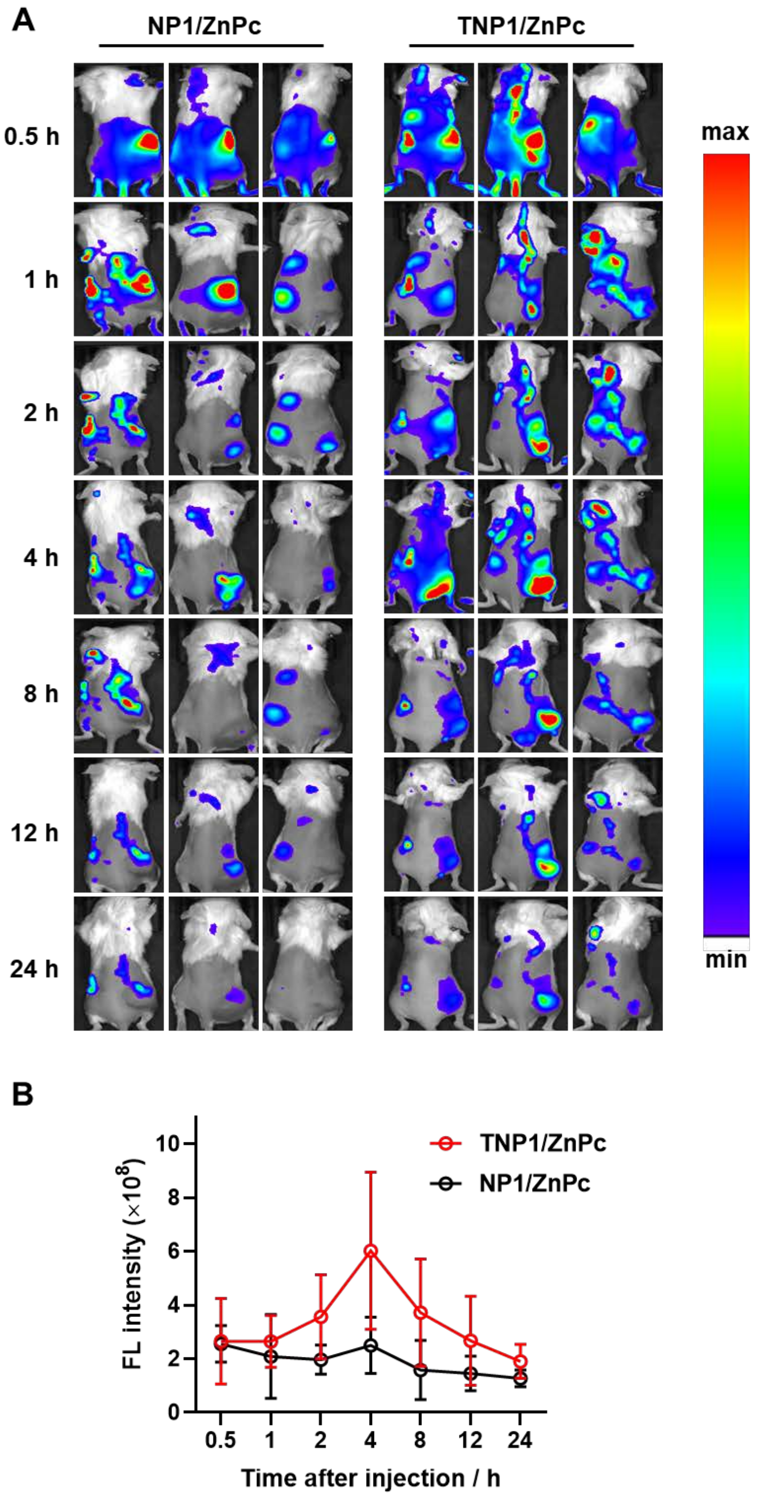

Figure S51. In vivo imaging of 4T1 tumor-bearing BALB/c mice at 0.5, 1, 2, 4, 8, 16, and 24 h after intravenous injection of NP1/ZnPc@Cy5 or TNP1/ZnPc@Cy5. (B) Statistical analysis of the quantitative fluorescence signals in the tumors after intravenous injection of NP1/ZnPc@Cy5 or TNP1/ZnPc@Cy5. Mean ± SD, *n* = 3.

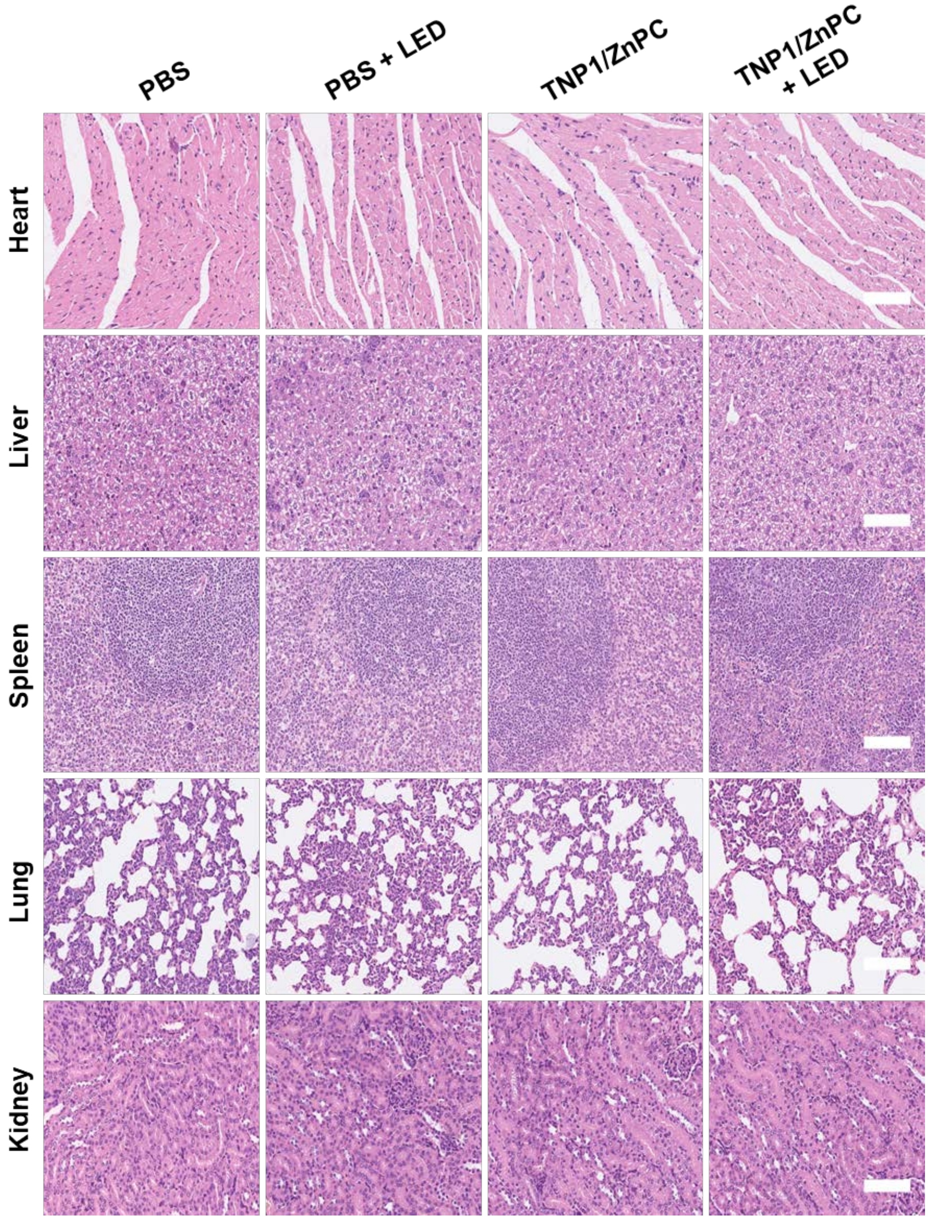

Figure S52. H&E staining images for histological analysis of major organs (heart, liver, lung, kidney, and spleen) collected from 4T1 tumor-bearing mice in different treatment groups. Scale bar, 100 μm.

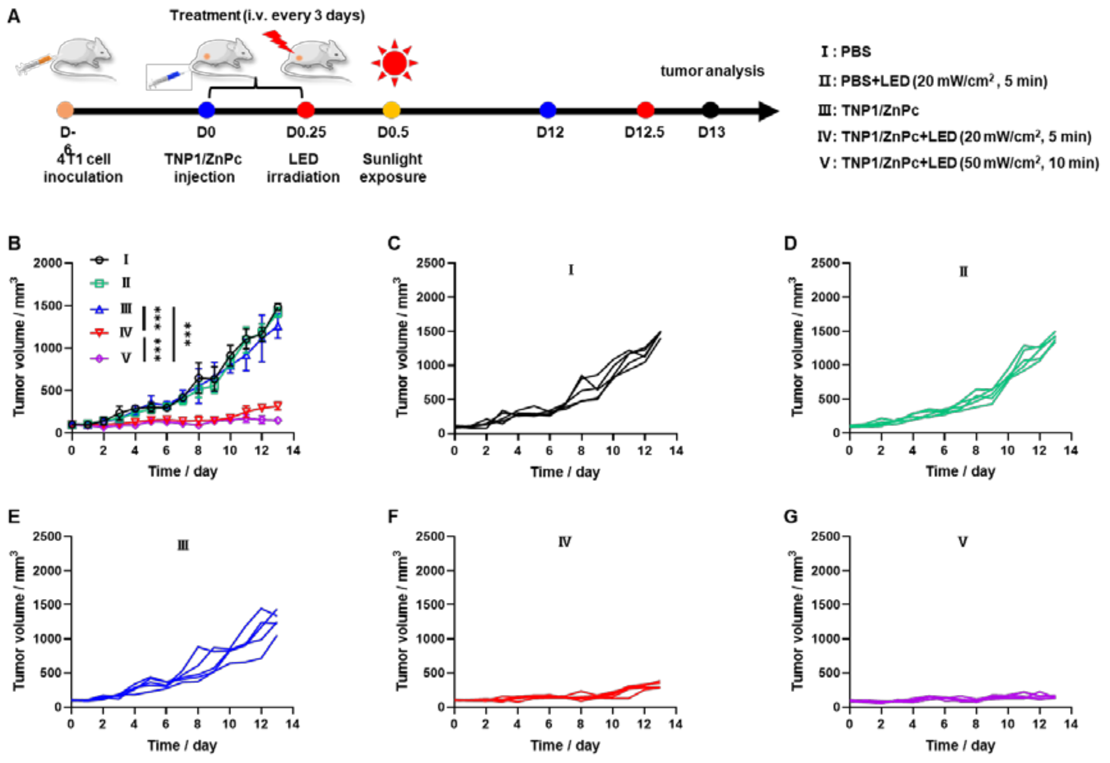

Figure S53. (A) Schematic illustration of the schedule for subcutaneous 4T1 tumor model inoculation and the therapeutic processes. (B-G) Tumor growth curves of mice after different treatments, including (I) PBS, (II) PBS+LED (20 mW/cm^2, 5 min), (III) TNP1/ZnPc, (IV) TNP1/ZnPc+LED (20 mW/cm^2, 5 min), and (V) TNP1/ZnPc+LED (50 mW/cm^2, 10 min), respectively. n = 5, ***P < 0.001.

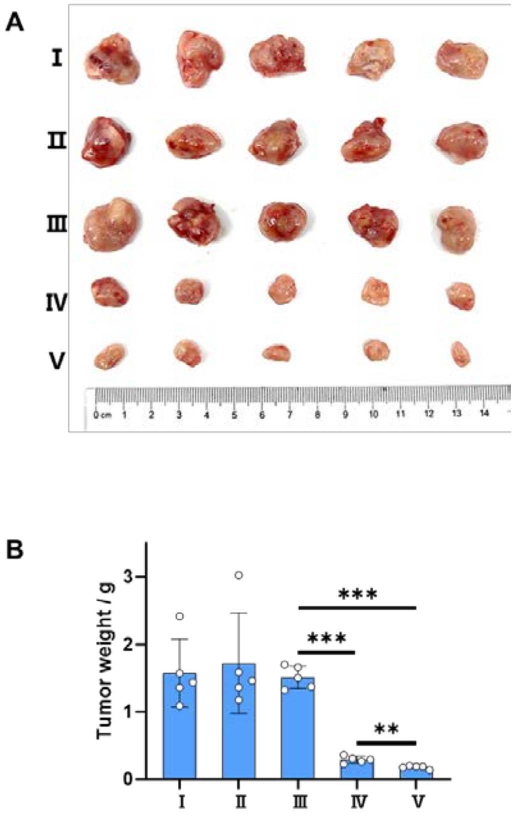

Figure S54. (A) Representative photograph of the excised tumors after 13 days various treatments. (B)Weights of 4T1 tumors excised at the termination of the experiments (n = 5). (I) PBS, (II) PBS+LED (20 mW/cm^2, 5 min), (III) TNP1/ZnPc, (IV) TNP1/ZnPc+LED (20 mW/cm^2, 5 min), and (V) TNP1/ZnPc+LED (50 mW/cm^2, 10 min), respectively. **$p < 0.01$. ***$p < 0.001$.

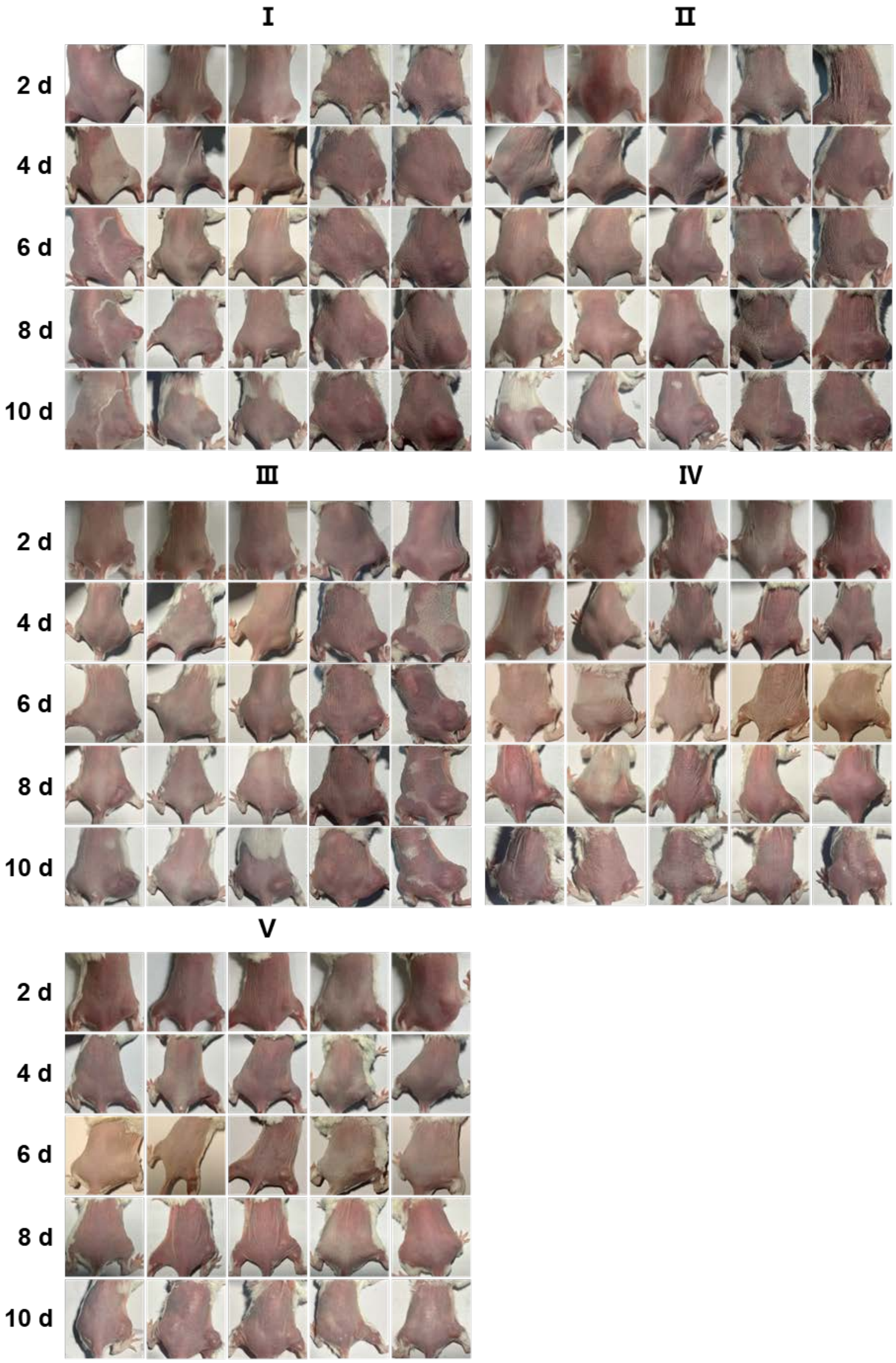

Figure S55. Skin phototoxicity monitoring of Tumor-bearing mice after simulated sunlight exposure during PDT treatments. (I) PBS, (II) PBS+LED (20 mW/cm^2, 5 min), (III) TNP1/ZnPc, (IV) TNP1/ZnPc+LED (20 mW/cm^2, 5 min), and (V) TNP1/ZnPc+LED (50 mW/cm^2, 10 min), respectively.

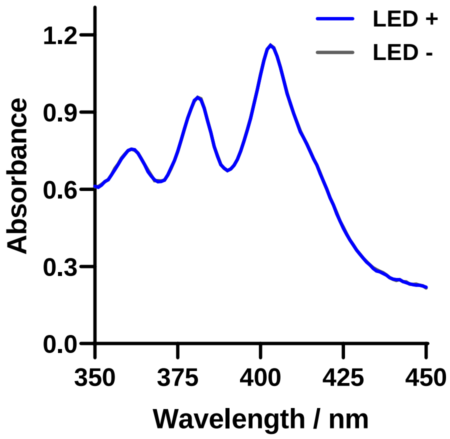

Figure S56. The stability of the PDT OFF in TNP1/ZnPc after 2 h exposure to fetal bovine serum were detected by ABDA (50 µM) under 660 nm LED light irradiation (20 mW/cm^2).

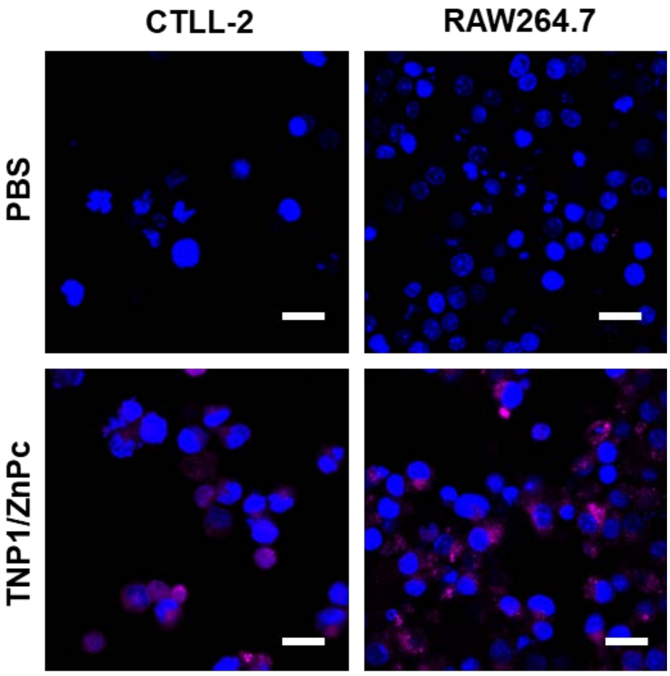

Figure S57. CLSM images of fluorescence intensity of CTLL-2 and RAW264.7 cells after incubation with TNP1/ZnPc for 2 h. ZnPc concentration: 5 μM. Scale bar, 20 μm.

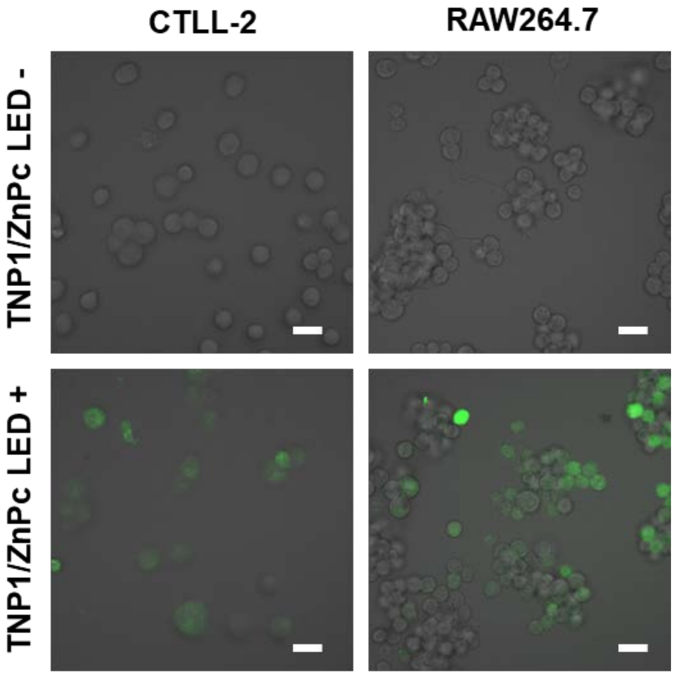

Figure S58. CLSM images of DCFH-DA incubated CTLL-2 and RAW264.7 cells after treatments with TNP1/ZnPc for 2 h in dark or upon LED light irradiation (660 nm, 20 mW/cm^2, 5 min). ZnPc concentration: 5 μM. Scale bar, 20 μm.

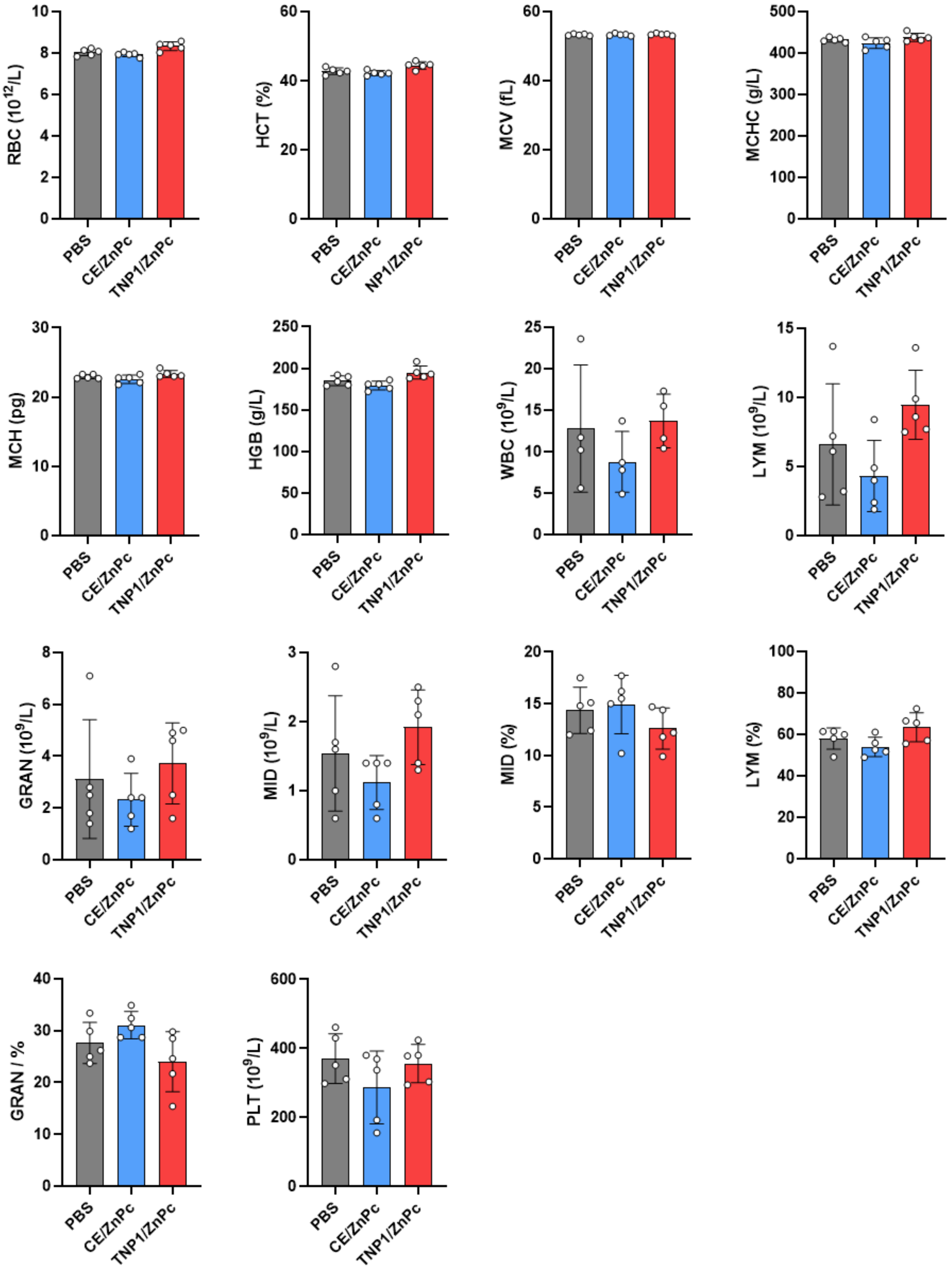

Figure S59. Hematological data of the mice intravenously injected with different samples at the 8th day post-injection.
